# Deep spatial-omics analysis of Head & Neck carcinomas provides alternative therapeutic targets and rationale for treatment failure

**DOI:** 10.1038/s41698-023-00444-2

**Published:** 2023-09-13

**Authors:** Andrew Causer, Xiao Tan, Xuehan Lu, Philip Moseley, Siok M. Teoh, Natalie Molotkov, Margaret McGrath, Taehyun Kim, Peter T. Simpson, Christopher Perry, Ian H. Frazer, Benedict Panizza, Rahul Ladwa, Quan Nguyen, Jazmina L. Gonzalez-Cruz

**Affiliations:** 1 Institute of Molecular Biology, The University of Queensland, Brisbane, QLD Australia; 2 Frazer Institute, The University of Queensland, Brisbane, QLD Australia; 3 Department of Medical Oncology, Princess Alexandra Hospital, Brisbane, QLD Australia; 4 Pathology Queensland, Royal Brisbane & Women’s Hospital, Brisbane, QLD Australia; 5 UQ Centre for Clinical Research, Faculty of Medicine, The University of Queensland, Brisbane, QLD Australia; 6 Faculty of Medicine, The University of Queensland, Brisbane, QLD Australia; 7 Department of Otolaryngology-Head & Neck surgery, Princess Alexandra Hospital, Brisbane, QLD Australia

**Keywords:** Oral cancer, Cancer screening, Tumour heterogeneity, Tumour immunology

## Abstract

Immune checkpoint inhibitor (ICI) therapy has had limited success (<30%) in treating metastatic recurrent Head and Neck Oropharyngeal Squamous Cell Carcinomas (OPSCCs). We postulate that spatial determinants in the tumor play a critical role in cancer therapy outcomes. Here, we describe the case of a male patient diagnosed with p16^+^ OPSCC and extensive lung metastatic disease who failed Nivolumab and Pembrolizumab/Lenvatinib therapies. Using advanced integrative spatial proteogenomic analysis on the patient’s recurrent OPSCC tumors we demonstrate that: (i) unbiased tissue clustering based on spatial transcriptomics (ST) successfully detected tumor cells and enabled the investigation of phenotypic traits such as proliferation or drug-resistance genes in the tumor’s leading-edge and core; (ii) spatial proteomic imagining used in conjunction with ST (*SpiCi*, Spatial Proteomics inferred Cell identification) can resolve the profiling of tumor infiltrating immune cells, (iii) ST data allows for the discovery and ranking of clinically relevant alternative medicines based on their interaction with their matching ligand-receptor. Importantly, when the spatial profiles of ICI pre- and post-failure OPSCC tumors were compared, they exhibited highly similar PD-1/PD-L1^low^ and VEGFA^high^ expression, suggesting that these new tumors were not the product of ICI resistance but rather of Lenvatinib dose reduction due to complications. Our work establishes a path for incorporating spatial-omics in clinical settings to facilitate treatment personalization.

## Introduction

Head and Neck Oropharyngeal Squamous cell carcinomas (OPSCCs) include cancers of the base of the tongue, soft palate, lateral and posterior pharyngeal wall, uvula, and tonsil^1^. Currently, OPSCC is one of the cancers with the fastest-rising incidences in high-income countries (225% prevalence increase in 20 years, USA)^2^, in both male and female populations^3^. This rise has occurred despite the reductions in alcohol and tobacco abuse over the past 20 years. In contrast, Human Papillomavirus (HPV) infections have emerged as the primary risk factor underlying the upward trend in OPSCC incidence^1^. In fact, HPV infection is responsible for 71%, 52%, and 63% of all OPSCCs in the United States, United Kingdom, and Australia, respectively^4–6^.

HPV^+^ OPSCCs are associated with a better outcome due to their higher susceptibility to chemoradiotherapy. Despite this favorable prognosis, 10–25% of HPV^+^ OPSCC patients will develop disease recurrence, mainly within the first 2 years after initial diagnosis^7^.

In 2016, the immune checkpoint inhibitors for programmed death receptor 1 (PD-1), Pembrolizumab and Nivolumab, received accelerated US Food and Drug Administration approval as a second-line treatment for recurrent/metastatic Head and Neck Squamous Cell Carcinomas (HNSCCs)^8,9^. Although HPV^+^ OPSCC patients have a better response rate to anti-PD-1 than HPV^-^ patients (25% vs 14%)^9^, overall, only a small percentage of HNSCCs (<20%) benefit from this approach, highlighting the important role of inter- and intra-tumor heterogeneity in treatment response and the need for patient-specific approaches.

Recent development in spatial-based high-throughput technologies, such as spatial transcriptomics (ST) and spatial proteomics (SP), has made it possible to assess cell subpopulations while maintaining the spatial architecture of the tissue. Thus providing an unprecedented level of knowledge about complex biological systems that involve multiple cell types including tumor development and response to treatment^10^. We reason that this new spatial information can assist in resolving tumor heterogeneity and can aid in getting the correct medication to the right patients, which is especially crucial following second-line Immune checkpoint inhibitor (ICI) failure.

**Fig. 1:**
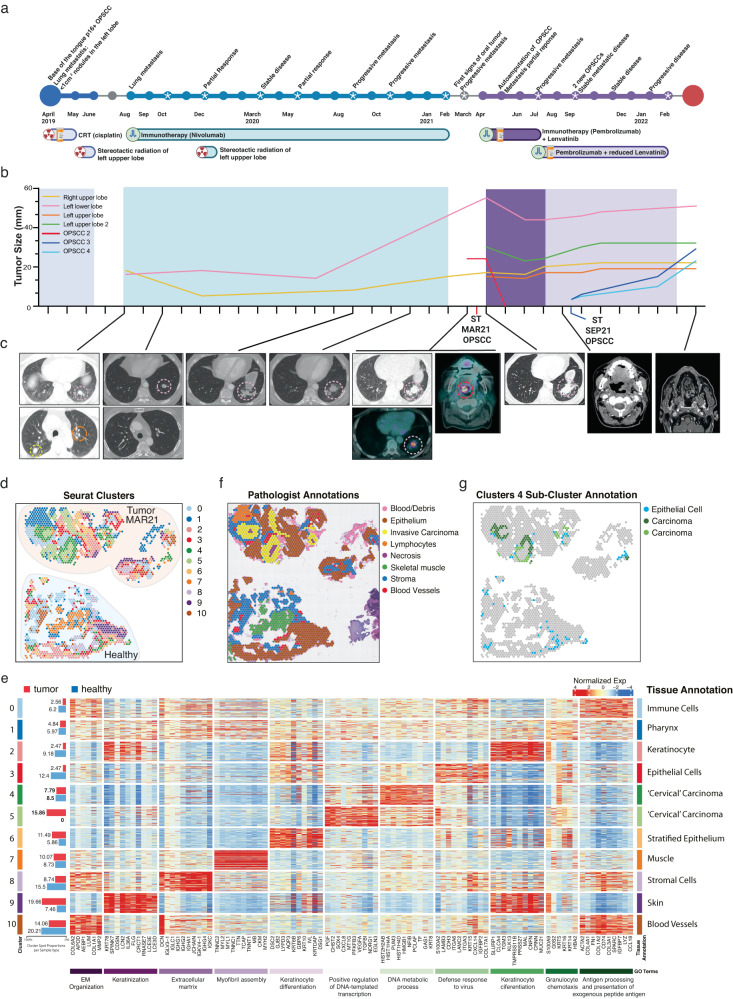
Case history and preliminary ST analysis of metastatic recurrent HPV^+^ OPSCCs. **a** Timeline indicating disease progression and treatment history. **b** Relative size and development of OPSCCs and lung metastases over time. Red line indicates the MAR21 soft palate OPSCC sample biopsied for Visium ST and PhenoCycler SP analysis. Dark blue line represents recurrent SEP21 OPSCC analyzed using Visium ST. **c** CT scans of lung metastases and OPSCCs over time. Circled regions highlight tumor tissue. **d** Spatial representation of unsupervised ST-generated clustering results. Colored spots represent different populations of spots that share similar transcriptional profiles. **e** Normalized expression values of the top 10 distinguishing markers for each cluster were displayed in a heatmap. JENSEN TISSUEs annotations and relative enriched Gene Ontology (GO) terms associated with differentially expressed genes (DEGs) for each cluster are also represented. Bar graph (left) represents the proportion of each cluster found within MAR21 tumor (red) and healthy (blue) samples. **f** Pathologist annotations of MAR21 and paired healthy sample, defining general tissue structures including stroma (blue), skeletal muscle (green), and invasive carcinoma (yellow). **g** Sub-clustering of cluster 4 identified distinct carcinoma sub-clusters, which were localized only within the tumor sample (green and dark green). Blue spots represent the third sub-cluster (cluster 4.Epi) which was annotated as epithelial tissue based on DEGs (using JENSEN TISSUEs database).

In this case study, we focus on a 60-year-old male patient diagnosed with HPV^+^ positive SCC primary tumor of the right tonsil and left upper lobe lung metastasis (Fig. 1a–c). After cisplatin chemotherapy and radiation targeting the primary tumor and bilateral neck nodes, plus stereotactic radiation to the left lung, the patient demonstrated multiple new bilateral pulmonary metastases (Fig. 1c). He then commenced Nivolumab treatment, which resulted in the resolution of two lung nodules and a significant reduction in a third (Fig. 1b, c, yellow line), but minor growth of a left lower lobe nodule (Fig. 1b, c, pink line). Unfortunately, after 13 months, the patient displayed progressive lung disease (Fig. 1b, c, pink line) and local recurrence with a new lesion of the left soft palate, which was biopsied for ST (MAR21) (Fig. 1b, c, red line). The patient was then enrolled in the LEAP-009 study where he received Pembrolizumab and Lenvatinib with an early partial response, including autoamputation of the oropharyngeal recurrence, which caused non-healing ulceration of the oropharynx. The patient resumed treatment with Pembrolizumab and dose-reduced Lenvatinib, but suspicious mucosal changes over the tonsillar fossa and soft palate were detected and biopsied for ST (SEP21) (Fig. 1b, blue lines). Shortly after, disease progression involving the oropharynx was confirmed, and the patient left the trial (Methods, “Case presentation (extended)”).

Here, using this case report exemplifying the need for tools to better select and tailor therapy, we demonstrate the clinical value of spatial proteogenomic data to rapidly and comprehensively resolve patient’s disease heterogeneity, identify tumor cells and generate quantitative data that inform about alternative druggable targets with the highest likelihood of delivering a personalized therapeutic response (Supplementary Figure 1).

## Results

### Spatial transcriptomic mapping of tumor and healthy tissue comprehensively distinguishes cell distribution and composition at a level not achievable by traditional methods

While methods like MRI and PET-CT scanning can capture general changes in the size and location of the tumor, deeper analysis of the cancer cells, expression of drug targets, and tumor microenvironment are required for a more accurate view of disease status. Due to its ability to produce whole-transcriptome resolution (>22,000 transcripts per tissue section), while maintaining spatial information and tissue morphology (a histopathological image accompanied by pathological annotation), spatial transcriptomic 10x Visium was chosen to analyze MAR21 OPSCC tumor and a healthy soft palate control sample (Supplementary Figure 2A). Unbiased clustering based on gene expression profile similarity identified 11 distinct clusters that closely recapitulated the tissue (Fig. 1d). Each spot cluster was manually curated using the JENSEN tissue-gene association database (Fig. 1e, Supplementary Figure 3A), with results closely matching those independently supplied by the pathologist (Fig. 1f), whereby clusters 4 (CL4) and 5 (CL5) overlayed the tumor sites (Fig. 1d, f). In addition to the main cancer clusters, other cell/tissue types were annotated, providing a comprehensive view of the entire tissue section, including epithelium (CL3), muscle (CL7), blood vessels (CL10), and pharynx (CL2) (Fig. 1e). Of note, carcinoma clusters were annotated with the cervical adjective due to the HPV^+^ OPSCC gene signature commonalities with cervical cancer and the overrepresentation of the former disease in the JENSEN database^11^. Thus, cancer clusters (annotated as “Cervical” carcinomas) orientated in a nest-like structure (keratin pearls corresponding to differentiated OPSCC), with CL4 being the edge and CL5 the core of the tumor. In contrast to CL5, which was exclusively found within the tumor biopsy (Fig. 1e, Supplementary Figure 3B), 30% of CL4 was located within the healthy tissue (Fig. 1d, Supplementary Figure 3). In-depth analysis of CL4, allowed us to re-annotate spots in CL4 into three categories, whereby the spots present in both the healthy tissue and the tumor were confirmed as epithelium, whereas spots that were annotated as carcinoma were only present in the tumor (Fig. 1g). These results highlight the heterogeneous nature of the tumor biopsy and the capability of ST to distinguish between tumor regions and healthy epithelial tissue based on transcriptional profiles. ST identified two different tumor clusters based on their transcriptional features and enriched in specific biological processes including DNA metabolism (tumor margin CL4) and transcription (tumor core and necrotic areas CL5) (Fig. 1e). Such an in-depth source of information is not achievable by standard pathological annotation and is crucial to accurately assessing the nature of the disease and the potential effects of drugs on various cell types across the tissue.

### Transcriptome-wide analysis of spatial gene expression identifies two distinct tumor microenvironments

**Fig. 2:**
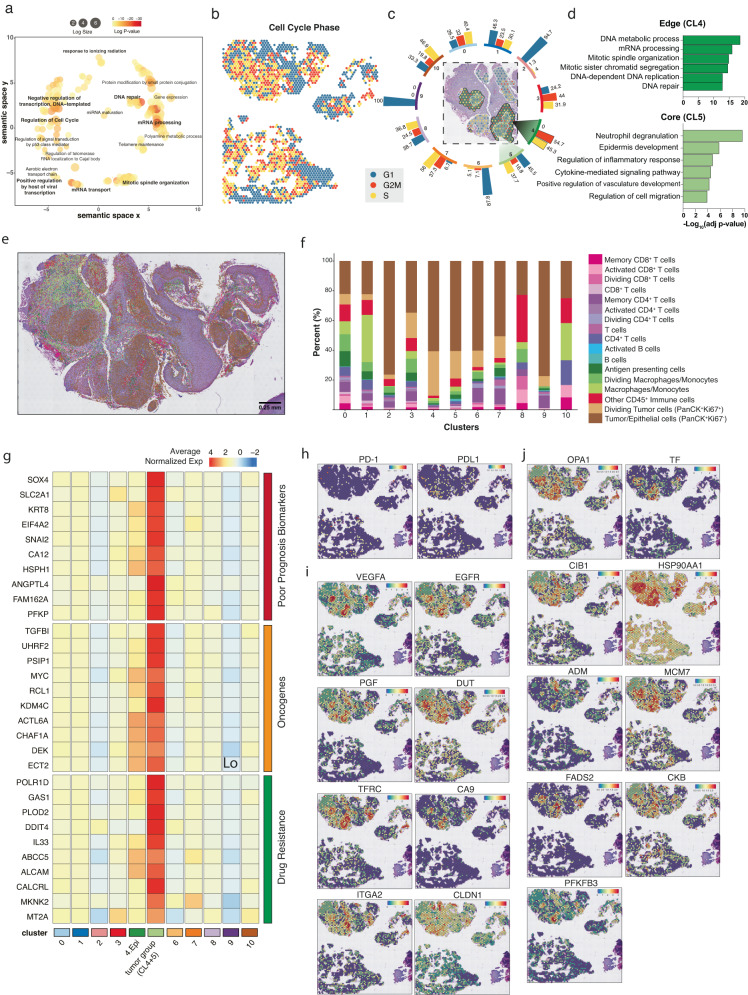
Transcriptional and functional profiles of the tumor microenvironment interface. **a** Common GO terms enriched in all tumor-annotated spots based on DEGs relative to all other clusters. **b** Cell cycle states of each spot within MAR21 OPSCC and healthy paired samples. Teal, red, and yellow represent spots in G1, G2/M, and S phase, respectively. **c** Displays the proportions of spots in each cell cycle phase for each Visium defined cluster. Teal, red, and yellow bars represent G1, G2/M, and S phase, respectively. Distinct tumor regions (cluster 4 and 5) are out-lighted in cluster-matched shades of green (dark green: cluster 4; light green: cluster 5). **d** Gene ontology terms specifically enriched in each tumor cluster. Significant GO terms were generated based on DEGs between distinct cancer clusters, newly defined Visium clusters 4 (dark green) and 5 (light green). Negative log transformed adjusted *p*-values were calculated using the Benjamini-Hochberg Procedure. **e** Single-cell resolution of cell types within the MAR21 sample based on integrated PhenoCycler data. Cell phenotype was based on co-expression of marker antibodies. **f** Stacked bar graphs highlight the percentage of different PhenoCycler cell subtypes observed within each Visium cluster. Colors are paired to indicate similar cell types (e.g., pink and purple are T cell subtypes, blue shades are B-cell subtypes). **g** Functional classification of DEGs (Wilcoxon test *p*-value < 0.001) of the combined tumor clusters relative to all other clusters and at least 2-fold overexpressed (CL4 and CL5). Relative expression of poor prognosis markers (red), oncogenes (orange), and drug resistance genes (green) across each cluster. The top 9 genes of each category were displayed. Cluster ‘4.Epi’ represents the sub-cluster 4 annotated as epithelial tissue, and ‘tumor group’ represents the carcinoma annotated spots from cluster 4 and 5 combined. Colors gradient represents average normalized expression values across all spots in each cluster, which were z-transformed by genes (rows of the heatmap). **h** Spatial expression of PD-1/PD-L1 encoding genes across each spot. **i** Spatial expression of genes targeted by current clinical therapies for various cancer types. **j** Spatial expression of experimental targets informed by preclinical studies. In all feature plots, the relative gene expression per spot is indicated by the colored scale bars. Both clinical and preclinical druggable targets were identified based on genes significantly overexpressed (Wilcoxon test *p*-value < 0.001 and >2-fold change) within the grouped tumor clusters.

Over 5000 out of >22,000 genes were significantly overexpressed in the CL4 and CL5, with signatures enriched for gene ontology terms associated with mRNA processing and transport, DNA regulation and repair, and cell cycle regulation (Fig. 2a). Sustained proliferation is a hallmark of cancer, and the number of cells in mitosis is used for diagnosis and to grade these malignancies^12^. Therefore, we investigated the spot’s proliferative status based on the expression of cell-cycle-related genes (Fig. 2b). Previously defined clusters showed different proportions of spots in each G1, S, and G2M phase. “Skin-related” (sharing epithelial origin) clusters such as CL2, 6 and 9 showed comparatively low proliferative profiles (Fig. 2c). Remarkably, of all 11 clusters, the CL4 was the only one that contained 100% spots in the proliferation phase (S with 45.3% and G2/M at 54.7%), indicating active cell division and expansion of this cancer cluster. As the cluster mapping and histopathological features suggested that CL4 and CL5 form two layers, with a less-proliferative core (CL5) and a rapidly dividing peripheral (CL4), we then sought to analyze the genes that were differentially defining tumor CL4 and CL5 (Fig. 2d). CL4’s differentially expressed genes (DEGs) confirmed the proliferative nature of the cluster with upregulation of *HIST1H* family genes, which participate in nucleosome assembly and chromatin organization, and *GABRP*, which promotes cell proliferation in oral SSC models (Supplementary Figure 4). Conversely, CL5’s DEGs were enriched with genes involved in innate immune response, inflammatory processes cell migration and angiogenesis, such as *CXCL8*, which attracts neutrophils, basophils, and T-cells or *S100A7*, involved in the activation of the innate immune response to viruses (Supplementary Figure 4). The spatial distribution of cell-cycle-related genes allowed us to distinguish 2 distinctive tumor phenotypes (high-CL4 and low-CL5 proliferation) with potential clinical implications especially when using replicative stress or DNA-damaging agents, as differences in replication can correlate with different responses to treatment^13,14^.

### Integration of spatial proteomics data enabled the mapping of 14 immune subtypes to spatial transcriptomics spots

As infiltration and localization of immune cells in the tumor microenvironment (TME) are biomarkers of disease progression and treatment outcome, we sought to estimate the cell composition of each cluster in the tumor biopsy at single-cell resolution. Although Visium data produces high-resolution transcriptional data, each spot is a mixture of on average 1–9 cells. Typically, spot deconvolution is needed to identify the cellular composition of each spot. Although computational models have been designed to optimize deconvolution performance, they are limited by the quality of the reference dataset, where incomplete or incorrectly annotated cell types will impact the deconvolution accuracy^15,16^.

First, we utilized a comprehensive publicly available single-cell OPSCC reference dataset^17^, to deconvolve our Visium data using five well-established RNA-based methods (STdeconvolve, CARD, Seurat label transfer, RCTD and Tangram)^15,16,18–20^. Comparatively, we found that each deconvolution method displayed very diverse cellular compositions (Supplementary Figure 5A). Especially, Tangram, over-estimated cell proportions (Supplementary Figure 5B), while the remaining four methods were relatively insensitive for detecting tumor infiltration cells. Therefore, to gain more sensitivity in the resolution of the proportions of immune cells, we devised a new method to integrate PhenoCycler spatial proteomics (SP) images with ST data that is independent of external reference datasets. By mapping consecutive tissue sections, we were able to detect the signal of multiple antibodies to map cell types of the same tissue and count the cells mapped to Visium spots (Fig. 2e, Supplementary Figure 6). Overall, our approach identified 14 different immune cell types from both the lymphoid (T cells, B cells) and myeloid (macrophages and antigen-presenting cells) lineages (Fig. 2f, Supplementary Figure 6). In our comparison of *SPiCi* (SP-inferred Cell identification) with the ground truth pathological annotation (Supplementary Figure 7), we found that our method exhibited high accuracy rates (0.943) (Supplementary Figure 7A). This indicates that *SPiCi* correctly resolved the tumor and immune composition of the Visium spots (Supplementary Figure 7B). Therefore, *SPiCi* offered an excellent solution to infer cell proportions when representative RNA data to use as a reference is not available (i.e., single-cell sequencing data from the same tissue). This integration of complementary data types is powerful as we can simultaneously use the highly confident cell-typing analyses based on SP and add transcriptomics-based information on cell states, pathway activities and signaling regulations as discussed in the next sections.

### High-resolution cellular composition of the tumor defined by *SPiCi* suggests tumor functional organization

*SPiCi* cell identification confirmed our cell-cycle prediction at the protein level by detecting high expression of Ki67 (S-G2/M phase surrogate) in the tumor nests (intra-tissue PanCK^high^) (Supplementary Figure 8). Once more, CL4 recorded the highest proportion of Ki67^+^ PanCK^+^ cells (29% of CL4) (Fig. 2f) forming the walls of the tumor as previously seen in the Visium ST data (Fig. 2c). Conversely, CL5, composed of the inner core of the tumor, displayed lower proportions of dividing tumor cells (18.4%) and higher levels of immune cells (21%) compared to CL4 (9.5% immune cells). This inner tumor core also contained high levels of macrophage/monocytes and CD4^+^ and CD8^+^ T-cells. Collectively, our spatial proteo-transcriptomics data confirmed the presence of two distinct tumor regions representing the proliferating leading edge and immune infiltrated inner core of the patient’s OPSCC, which phenotype and cell-type complexity could not be resolved by histopathological assessment (Fig. 1f, Supplementary Figure 6).

### The spatially defined tumor microenvironments informed the assessment of predictive biomarkers and druggable targets

Traditional methods such as bulk RNA sequencing suffer from dilution of cancer-specific signals within the pool of non-malignant tissue. We hypothesized that our ST data could overcome this by focusing on the most important part of the tumor, namely the cancer tissue depicted by only 2 clusters out of the 11 that composed the biopsy. This spatially-focused analysis strategy maximizes resolution while minimizing signal dilution as seen in bulk data analysis. By doing so, we identified 158 significantly upregulated (*p* < 0.001) tumor genes, which displayed at least a 2-fold increase compared to all other clusters (Supplementary Figure 9). Most top genes were categorized as poor prognosis biomarkers (38%), oncogenes (13%), and drug resistance genes (9%), in contrast to only a 11% and 5% considered tumor suppressor and positive prognosis biomarkers, respectively (Fig. 2g, Supplementary Table 3). Notably, the function of 6% of the >2-fold increased tumor genes remain unknown and may potentially be new markers. These results correlate with the observed recurrent and aggressive behavior of the patient’s tumor and highlight the potential of focused transcriptional profiles to predict disease progression.

The patient’s treatment history pinpoints two specific proteins targeted by immunotherapy and chemotherapy: PD-1 (Nivolumab, Pembrolizumab) and VEGFR (Lenvatinib). Interestingly, we found that both PD-1 and its ligand PD-L1 encoding genes (Fig. 2h) and proteins (Supplementary Figure 10) displayed low expression across the whole tissue. Indeed, these two targets were not expressed in the two cancer regions, potentially explaining why ICIs failed. Conversely, amongst the 158-overexpressed tumor genes, there were 8 targets with inhibitors either commercially available or in clinical trials, including *EGFR*/cetuximab-prochlorperazine (Fig. 2i)^21^ and 9 experimental targets supported by preclinical data (Fig. 2j, Supplementary Table 4)^22^. Consequently, the use of ST has the potential to prevent the use of treatments that are unlikely to be effective, while simultaneously identifying novel therapeutic targets.

### Tumor clusters and targets were shared by pre- and post-treatment tumors and revealed potential causes of treatment failure/response

**Fig. 3:**
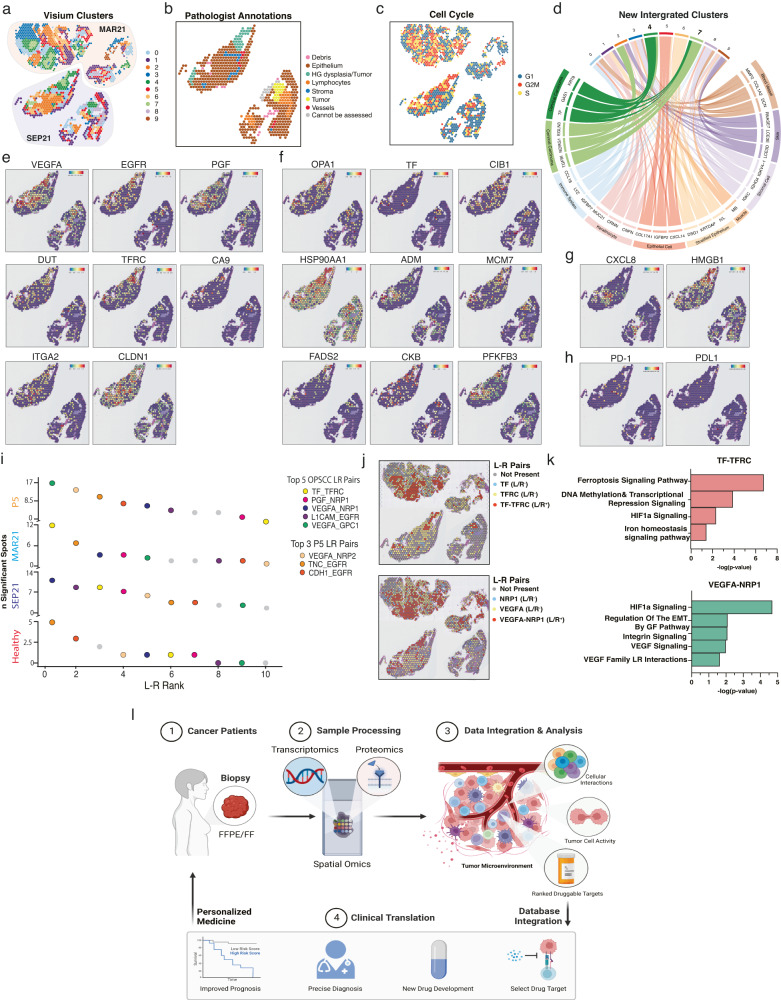
Transcriptional comparison between MAR21 and recurrent SEP21 and ligand-receptor interaction analysis for therapeutic target selection. **a** Spatial representation of unsupervised clusters identified between integrated tumor samples. MAR21 highlighted in red shades and recurrent tumor SEP21 highlighted in blue shades. **b** Annotated cell cycle phase of each spot based on relative expression of specific cell phase genes. **c** Pathologist annotations of SEP21, defining general tissue structures including epithelium (brown), dysplastic tissue (light green), and tumor (yellow). **d** Chord diagram displays genetic correlation between new clusters corresponding to integration of the MAR21 and SEP21. Comparisons were based on gene expression levels of the top 3 upregulated genes expressed by each original MAR21 cluster (only one muscle gene was found in the new cluster) within each new cluster. Connecting ribbons define the correlation between original and new clusters which significantly over-express each gene. **e** Spatial expression of genes across each spot targeted by current clinical therapies. **f** Spatial expression of genes targeted by select experimental drug therapies. **g** Spatial expression of genes targeted by select experimental drug therapies only seen in SEP21 samples. **h** Spatial representation of gene expression for Nivolumab and Pembrolizumab targeted *PD-1/PD-L1* pathway. In all feature plots, the relative gene expression per spot is indicated by the colored scale bars. **i** Ranking of top 35 ligand/receptor (L-R) pairs targeted by clinical and experimental therapies expressed by the healthy (red), MAR21 (light blue) recurrent SEP21 (dark blue), and additional patient (orange) samples. Rank was based on the number of significant spots expressing each L-R pair across each sample. Colors indicate the location of each specific LR pair. Orange spots represent the top 3 L-R pairs specific to the additional sample. **j** Spatial localization of ligand and receptor expression across tumor tissues (top: MAR21, bottom: SEP21). Red spots indicate co-expression of both ligand and receptor within the same spot. **k** Significantly active IPA pathways associated with expression of the top two ranked L-R pairs (TF/TFRC and VEGFA/NRP1). Bars indicate negative-log right-tailed Fisher’s Exact Test *p*-values of each pathway based on the proportion of genes within each canonical pathway that were also significantly up- or down-regulated within spots co-expressing both L-R pairs. **l** Infographic representing the proposed protocol to incorporate spatial multi-omics within a clinical setting. Image generated with Biorender.

In recurrent settings, it is imperative to investigate whether information obtained from a tumor can predict the phenotype of tumors to come. Thus, we spatially sequenced the SEP21 OPSCC tumor (failed combinational therapy) (Supplementary Figure 2C-B) and compared it to the previously sequenced MAR21 tumor (failed monotherapy) (Fig. 3a, Supplementary Figures 11, 12). Annotations based on transcriptional profiles were consistent with pathologists’ evaluation, showing that ST was capable of successfully identifying tumor cells (Fig. 3b). Furthermore, transcriptomic annotation aided the pathology team in resolving a conflictive area where low stroma abundance made it difficult to assess invasive cancer (tumor), highlighting the power of using unbiased molecular profiles to characterize tissue regions (Supplementary Figures 2B, C, 13). To compare cancer cells in both biopsies, we performed the same cell-cycle analysis (Fig. 3c) and unbiased clustering (Fig. 3d) as for MAR21 (Fig. 1d). The consistent results mapped the same carcinoma hubs in both biopsies (CL4 and CL7, Fig. 3a, d, Supplementary Figure 14). The new CL7 corresponded to CL5-MAR21 (Fig. 1d) plus the inner core of SEP21 and it was annotated as “cervical” carcinoma (Fig. 3d). Importantly, most of the identified MAR21 druggable and experimental targets were also overexpressed in the SEP21 tumor clusters (Fig. 3e–g, Supplementary Table 3). These druggable targets were identified independently in separate tissues and at different time points, suggesting the reproducibility of detecting potential targets, which colocalize to cancer regions and maintain a high expression level throughout time and space.

The initial lack of response to anti-PD-1 treatment and subsequent resurgence of a new locoregional tumor suggests evasion by a drug resistance mechanism. The deep analysis of spatial proteo-transcriptomics of the MAR21 tumor showed no expression of PD-1 in the tumor hubs, but rather scattered expression of this target across the tissue section in non-cancer cells, potentially being the cause for the lack of response. In contrast, the combinatorial therapies targeting PD-1 and VEGFR showed initial responses with tumor self-amputation, followed by tumor resurgence when VEGFR was reduced to aid with the patient’s coagulation/healing. Analysis of the SEP21 sample showed low PD-1/PDL-1 and high *VEGFA* levels at the tumor locations. Overall, our findings strongly suggest that the therapeutic response seen in the MAR21 OPSCC was not driven by Pembrolizumab but Lenvatinib, as its reduction correlated with the growth of recurrent VEGFA^high^ tumors previously suppressed by the chemotherapy.

### Novel strategy to prioritize targets based on ligand-receptor interactions

Treatment personalization is considered the future of many medical disciplines including oncology. As many targeted anti-cancer drugs act by blocking ligand/receptor interactions (L-R), we explored whether tailored treatment against the patient’s tumor-upregulated genes could be ranked based on the colocalization and activity of L-R pairings in the tumor microenvironment (Fig. 3i). Based on the expression levels of co-localized L-Rs in each sample (healthy, MAR21 and SEP21), we implemented the *stlearn* methods^23^ (Supplementary Figure 15) to rank L-R pairs known to be potential genetic targets (Fig. 3i, Supplementary Table S3–4). This classification allowed us to select the top-5 most active druggable L-Rs in the tumor *vs* the healthy tissue (Fig. 3i). Of these, *VEGFA/NRP1* and *TF/TFRC* were highly active in both the original and recurrent OPSCCs (Fig. 3i, Supplementary Figure 16). Spatial analysis confirmed the co-expression of these L-R pairs primarily within the malignant regions of both tumor samples, which would specifically focalize the treatment to the malignant tumor clusters (Fig. 3j). The selected targets were patient-specific, as the analysis of another individual’s HPV^+^ OPSCC, showed that although some interactions, such as *VEGFA/NRP1* and *VEGFA/GPC1* were present, the most active L-Rs in the tumor were *VEGFA/NRP2* and *EGFR*-related pathways (Fig. 3i, Supplementary Figure 17). The analysis of the additional patient sample showed that this patient had a high expression of PD-1 and PD-L1 in the cancer core region (Supplementary Figure 17C), a contrasting pattern compared to the case reported here, suggesting that this patient might be responsive to PD-1/PD-L1 drug (Supplemental Information, Case presentation of additional patient). Thus, spatial gene expression profiling can detect specific expression patterns of drug targets in each patient sample. Lastly, we confirmed that both *VEGFA/NRP1* and *TF/TFRC* interactions are likely to be biologically functional as DEGs from L-R-positive *vs* the L-R-negative spots were significantly enriched in canonical pathways associated with the downstream activation of these L-R pairs (Ingenuity Pathway Analysis, Fig. 3k)^24,25^. Overall, we identified that L-R co-expression and downstream pathway analysis can be used to prioritize clinical targets allowing clinicians to combine multiple strategies that will interfere with known tumor-activated pathways in a personalized manner.

## Discussion

The value of the global personalized medicine market is expected to reach USD 922.72 billion by 2030, a 6th of which will account for Oncology Precision Medicine alone^26^. However, tumor heterogeneity and constant disease evolution indicate that patients will only benefit long-term from tailored medicine if they are based on analytical pipelines that are as holistic and dynamic as the disease itself. Studies using spatial-omics techniques to understand cancer disease development and progression are becoming increasingly more common in oncology research^27^. Here we sought to explore the use of spatial multi-omics in a clinical context, to determine its appropriateness as a potential medical tool for recapitulating patient disease progression and aiding in the drug selection and combination processes.

Based on spatially defined, differentially expressed genes, here we resolve intra-tumor heterogeneity and confidently annotate diverse tissue types including malignant and healthy, stroma and infiltrating immune cell populations. Two clusters (CL4 and 5), which overlay the cancerous and necrotic regions defined by pathologist annotations, are highlighted by multiple lines of unbiased analyses as carcinoma tumor communities showing differential signatures: CL4, highly proliferative leading-edge *vs* CL5, core enriched in immune infiltrates interacting with tumor cells and necrotic parts (Fig. 1). Detection of two cancer clusters, with distinct molecular, but not morphological phenotypes, is an important result of our data-driven approach. Although, Ki67 protein detection confirms the proliferative nature of CL4, the expression of cell-cycle-phase associated genes predicts a higher degree of cells in S-G2/M phases (Fig. 2c). This suggests that the ST method is more sensitive at detecting dividing cells by assessing hundreds of cell-cycle-related genes in contrast to a sole marker as immunohistochemistry methods usually use^12^. Therefore, this method could aid the assessment of the tissue mitotic activity in an unbiased manner, pinpointing regions with heterogeneous metabolic profiles that could impact prognosis and response to treatment targeting replicative stress. Importantly, the cytotoxicity of certain chemotherapies such as Cisplatin and Paclitaxel, depends on the tumor proliferative state and cell cycle phase^13,14^. For instance, repeated dosages of Paclitaxel were more efficient when given in cell culture to cells preparing for G2/M phase^14^. ST data of core biopsies taken at different time-points during treatment could be an excellent tool to test whether timing optimization of sequential chemotherapeutics improves their cytotoxic capacity in a clinical setting or whether low proliferative tumor regions are responsible for chemotherapy failure.

To note, all analyses performed were conducted prior to receiving tissue annotations, emphasizing the true unbiased nature and discovery strengths of our approach and findings. For the second tumor (SEP21), the transcriptomic signature also informs pathologists of tissue areas with suspicious features, which facilitates the resolution of conflict zones resulting in a more assertive annotation of the dysplastic regions. Here we prove that unbiased annotation based on ST data recapitulates clinical annotations and can assist pathologists especially when the quality/size of the sample is not optimal for visual macroscopic characterization. Although, more data needs to be collected and included in reference databases to enhance the accuracy of the annotations (i.e., HPV^+^-cervical cancer *vs* HPV^+^-OPSCC), overall, the ability to precisely define these cancer regions and assess specific markers differentially expressed by them has a great potential for drug selection.

The composition, location, and interactions of immune infiltrates within the tumor play crucial roles in determining treatment outcomes^28–30^. Such interactions ideally require single-cell resolution data and detailed identification of immune cell types. However, the current Visium ST technology suffers from relatively low cell resolution as ST ‘spots’ encompass average gene expression across several cells (1–9 cells). To address this issue, we develop a novel spatial multi-omics cell identification approach (*SPiCi*) by integrating Visium ST and PhenoCycler SP images which identifies tumor cells and lymphocytes with high accuracy (Supplementary Figure 7). Importantly, our method has the additional advantage of considering both protein and transcriptional data when studying tumor and TME interactions, which increases the robustness of cell identification, especially of immune cell populations, by overcoming the problem of the non-linear relationship between RNA expression and protein levels^31–34^. In our study, highly proliferative epithelial cells are annotated as proliferating tumor (Fig. 2e, f). However, because *SPiCi* is based on multiplexing PhenoCycler technology, its accuracy can be improved by increasing the number of markers scanned, which is now greater than 100-plex. Using our protein/RNA-based method and a panel of 11 markers we identify 14 different immune subtypes, including activated CD4^+^ and CD8^+^ T-cells, macrophages and B cells present within the inner tumor. Our method enables the association of the cell type to its corresponding transcriptional profile. This information can correlate cell subsets with disease progression and response to treatment as previously seen for HNSCC, where certain CD4^+^ and CD8^+^ T-cell phenotypes correlated with longer progression-free survival^35,36^.

The spatial component of our analysis allows us to focus on the critical tumor sections (CL4-5), where we observe that most upregulated genes are either oncogenes, poor prognosis biomarkers or drug resistance genes, confirming the active and aggressive nature of our patient’s disease^37^. In the clinic, this information could help stratify patients and tailor surveillance plans based on expected disease behavior. The high-throughput nature of our approach allows answering questions such as, which suitable targets the tumor hubs are expressing. In spite PD-1/PD-L1 expression being very low, we identify 8 overexpressed druggable targets (i.e., *EGFR, TF, VEGF*) and 9 preclinical targets in CL4-5. Although, future work will focus on proving the robustness between the presence/distribution of a target and therapy response, being able to interrogate the patient’s tissue for the presence or absence of multiple targets will save time, resources and psychological burden to patients and their families. Surprisingly, targets are mostly shared by the recurrent tumors within the same tumor areas, indicating that in this case the information gained from the first patient’s OPSCC biopsy is still applicable to the subsequent malignancy. However, considering the time and health constraints of recurrent non-responsive cancer patients, a list of targets might not guarantee patient’s long-term clinical response, thus a way to rank the target candidates is equally vital. Here we reason that the likelihood of a drug having an impact on tumor growth and progression would be linked with its capacity to interfere with vital and active pathways in the malignancy. Subsequently, here we design a novel strategy to rank each drug’s potential success, based on the co-expression of each target ligand-receptor pair (L-R), assuming that co-expression would lead to interaction, and hence pathway activation. After our analysis, our patient’s list of 17 clinical/preclinical targets was refined to 3 top druggable pathways active in the patient’s tumor clusters: *TFRC*, *NRP1*, and *EGFR*. Of note, *NRP1* is a SARS-CoV2 receptor, for which dozens of new drugs have recently been developed^38,39^, and hence may justify further clinical applicability in cancer.

Overall, our work demonstrates the power of Spatial proteogenomic data to resolve tumor heterogeneity and enables the possibility for oncologists to personalize cancer management. Following validation in larger cohorts, this case study supports applying spatial proteogenomic in clinical settings. We envision that spatial RNA/protein analysis can be adopted in clinical settings, as whole genome sequencing is now a routine test requested by clinicians. Importantly, the cost of these technologies and high technical requirements can already be drastically reduced by implementing artificial intelligence (AI) models capable of predicting in situ gene expression inferred from fast/low-cost H&E images using curated disease Spatial-omics training datasets^40–42^. Thus, after an initial investment dedicated to creating standardized disease-specific AI training material, the spatial data of each patient’s tumor biopsy can be obtained (experimentally or AI-inferred) and contrasted against spatial databases of the disease to help with different steps along each patient’s journey (Fig. 3l, Supplementary Figure 1): (i) aid in the annotation of the tumor, (ii) stratify patients based on disease risk progression to personalize surveillance plans, and (iii) to generate a list based on a patient’s disease features which inform oncologists of targets with quantifiable likelihood to have an impact on the disease. This information can be used to implement combinatorial therapeutic programs to prevent drug resistance minimizing off-target effects.

## Methods

### Case presentation (extended)

A 60-year-old male ex-smoker with a 10 pack-year history presented with de novo oligometastatic disease, having been diagnosed with a p16 positive SCC primary tumor of the right tonsil and biopsy-proven left upper lobe lung metastasis (Fig. 1a–c). He received concurrent chemoradiotherapy comprising weekly cisplatin and 70 Gy in 35 fractions of radiation targeting the primary tumor and bilateral level II and III neck nodes (Fig. 1a). In addition, he received stereotactic radiation to the left lung nodule of 50 Gy in 5 fractions, completed in June 2019.

The patient underwent disease re-assessment three months later, with PET/CT demonstrating multiple new bilateral pulmonary metastases and no locoregional disease (Fig. 1b, c). He commenced treatment of 480 mg nivolumab every 4 weeks and a CT scan after three cycles revealed resolution of two nodules and a significant reduction in a third (Fig. 1b, c, yellow line), but minor growth of a left lower lobe nodule (Fig. 1b, c, pink line). This single nodule was then treated with stereotactic radiation of 48 Gy in four fractions and nivolumab was continued. Regular imaging confirmed stable intrathoracic disease for a further 13 months but a PET/CT scan in February 2021 demonstrated local recurrence of disease with a 22 mm lesion of the left soft palate (biopsy MAR21) and progression of metastatic lesions in the lungs bilaterally.

The patient was then enrolled in a clinical trial (LEAP-009) and randomized to the treatment of pembrolizumab 200 mg every 3 weeks and 20 mg Lenvatinib orally daily. After demonstrating an early partial response radiologically, including autoamputation of the oropharyngeal recurrence with absence of measurable disease and a reduction in lung metastases, a treatment delay was required due to oropharyngeal pain and severe non-healing ulceration of the oropharynx. This subsequently settled and the patient recommenced pembrolizumab and dose reduced Lenvatinib (14 mg orally daily) in July 2021. The measurable disease remained stable radiologically until January 2022, although mucosal changes over the tonsillar fossa and soft palate were suspicious clinically from September (biopsy SEP21). Pembrolizumab and Lenvatinib were ceased, and the patient came off trial in early February 2022 when MRI scan confirmed definite disease progression involving the oropharynx.

### Case presentation of additional patient

A 63-year-old male non-smoker presented with de novo keratinizing p16 positive SCC primary tumor of the left tonsil invading into the skeletal muscle with no nodal disease. He underwent transoral robotic-assisted resection (TORS) of tonsil and selective neck dissection of cervical lymph nodes in November 2020. No adjuvant radiotherapy was given. A two-year MRI follow-up indicates no tumor masses or recurrent disease.

### Pathologist annotations

High resolution H&E images of samples from two patients were provided to a pathologist for pathological and tissue annotation. Loupe Browser was used by the pathologist to outline and label various morphological features observed within the H&E tissue, which were then converted to a ‘Visium spot’ format. Annotations were blindly performed (the pathologist was not made aware of analysis results) succeeding all other analyses completed in this study.

### Spatial transcriptomics (ST)

Five μm sections were taken and multiplexed onto Visium Spatial Gene Expression Slides (10x Genomics). Following slide incubation (60 °C for 2 h) on a Thermocycle (Bio-Rad C1000 Thermal Cycler), H&E staining, imaging and sequencing library preparation was performed in accordance with the Visium Spatial Gene Expression User Guide (CG000407, CG000408, CG000409 – 10x Genomics). Trimmed FASTQ reads were mapped to the human reference genome (version GRCh38-3.0.0) using *SpaceRanger* (*v1.3.0*) and demultiplexed using 10X Loupe Browser (*v6.1.0*). Sequenced raw read and expression data were processed and overlaid with the H&E image (*Seurat*; *v4.0.5*). Samples were analyzed following a general pipeline that consisted of (1) data filtering and quality control, (2) normalization, (3) batch correction and integration, (4) unsupervised clustering, (5) differential expression analysis, and (6) other downstream analyses.

### ST data quality control

Raw read and expression count distributions were exploratorily analyzed to remove any poor-quality samples due to processing error (tissue dissociation, tissue folding, technical issues). An additional tumor section of sample MAR21 was removed due to poor-quality counts and tissue folding evident in the H&E image (Supplementary Figure 2). Each sample was analyzed for outlying spots containing low gene expression counts to mitigate technical error. Spots were filtered using a fixed threshold of <200 features per spot based on previous literature describing the required amount of genetic information necessary for a viable genome^43^.

### ST data normalization

Filtered data was normalized using *SCTransform* from Seurat to ensure differential gene expression patterns were due to biological variation rather than technical bias. This method implements a regularized negative binomial regression model based on unique molecular identifier (UMI) counts, to normalize expression data for each gene independent of total sequencing depth per cell^44^.

### ST batch correction and data integration

UMAP plots of normalized expression data were generated for each sample based on the first 30 principal components (PCs), which were selected using Elbow plots of variance. Batch correction and data integration between healthy and tumor MAR21 samples, and between MAR21 and SEP21 tumor samples, respectively, were performed using the *Seurat* canonical correlation analysis (CCA) data integration function (*IntegrateData*) (Supplementary Figure 11). Based on 3000 variable features calculated for each diverse dataset (using *FindVariableFeatures*), a list of ‘anchors’ encoding cellular relationships was generated through CCA and MNN analysis^18^. Following principal component analysis (PCA) informed dimensionality reduction, UMAP plots were generated for each integrated dataset (healthy/tumor and tumor/tumor) using the first 50 and 30 PCs, respectively. Other parameters for all functions were using default settings.

### ST unsupervised clustering

Unsupervised graph-based clustering was performed on each integrated dataset separately using the *Seurat FindClusters* function. This approach implements a shared nearest neighbors (SNN) modularity based on a resolution value set to 0.8 and 0.6, for healthy/tumor and tumor/tumor datasets, respectively. Cluster spot assignments were visualized on UMAP plots and spatially using the inbuilt *Seurat* method (*SpatialDimPlot*). Relative proportions of spots assigned to each cluster per tissue sample were also calculated and plotted using custom scripts.

Sub-clustering was performed on cluster 4 of the MAR21 integrated dataset to distinguish between spots within the healthy and tumorous tissue. This was achieved using *Seurat FindSubCluster* function which was set to implement Louvian clustering at a resolution of 0.5.

### ST differential gene expression analysis

Prior to analysis, cluster labels were mapped back to the original raw expression data, where spots were again filtered and normalized by *SCTransform* as described above. Differential gene expression analysis between clusters was performed on each dataset using the likelihood-ratio test implemented by *Seurat FindAllMarkers*^45^. Significance thresholds were set at *p* < 0.001 and average log2 fold change >0.5. Scaled expression values of the top 10 DEGs from each cluster (top six for clusters 1 and 10) were repressed in a heatmap using *ComplexHeatmap*^46^.

### ST cluster annotation

Clusters were annotated using *Enrichr*^11^, which makes use of fuzzy enrichment analysis to compare significant positive DEGs per cluster (i.e., most upregulated DEGs and likely markers for that cluster) to JENSEN TISSUEs ontology reference database (V2.0)^47^. Each cluster in both datasets was analyzed and assigned a tissue type based on the top-10 significant (adjusted *p*-value > 0.001) biologically relative hits (i.e., non-related tissue annotations were not considered).

### ST gene ontology enrichment analysis

DEG lists specific for each cluster were tested for GO term enrichment using *clusterProfiler*^48^. The *enrichGO* function and ‘org.Hs.eg.db’ human genome database was used to identify biological process terms significantly related to the DEGs (*p*-value > 0.01) of our datasets. The top significant term was assigned for each cluster and compared to the relative cluster annotation label. In addition, annotated tumor clusters within the healthy-tumor dataset were merged and a new list of DEGs and enriched GO terms was generated using the methods described above. All identified terms were grouped into common ontology tags and graphically represented using *Revigo*^49^.

### ST cell cycle analysis

*Seurat CellCycleScoring* function and the ‘cc.genes.updated.2019’ cell cycle gene references dataset^50^ were used to assigned cell state labels for each spot. This method determines cell phase state based on relative expression of G2/M and S phase maker genes. These genes are anticorrelated, meaning spots expressing neither gene set were annotated as G1 phase.

### ST ligand-receptor cell–cell interaction analysis for drug target prioritization

L-R analysis was performed using *stlearn*^23^. Briefly, the *p*-values are derived from independent testing of one L-R pair at a time and using the same set of genes for calculating the background signal (i.e., all detected genes). The test identifies spots (across all spots in the tissue section) with a significant co-expression of the L-R pair (considering neighboring spot information) that is higher than the background co-expression of any random combination of gene-gene pairs. As such, in the formulation of the background signal, this test does not only include all L-R pairs but all random gene-gene pairs in the dataset are included. The *p*-value is derived from the permutation count of events that the L-R has a higher score than the random scores of millions of random combinations. Because the background does not get affected by the subsetting of druggable L-R pairs, the raw *p*-value for each L-R pair does not change. The adjusted *p*-values after multiple testing correction change proportionally with the number of L-R pairs tested, resulting in fewer spots that are significant when testing for all L-Rs. Importantly, the rank for the L-R in terms of the number of significant spots or *p*-values/adjusted *p*-values remains unchanged. Below we explained further the rationale for our approach.

The analysis was done using the *connectomDB2020* L-R database^51,52^ (Supplementary Figure 16). Normalized gene expression values of only the druggable tumor overexpressed DEGs were used to calculate L-R pair expression between neighboring spots in integrated MAR21 and SEP21 tissues (Fig. 3i). Pairs were considered valid when both genes were DE by the tumor with respect to the non-tumor clusters. The top five significant results were reported as several significant spots found within each sample. DEGs between spots expressing and not-expressing each selected L-R pair were generated using previously described methods. Pathway analysis was performed using QIAGEN Ingenuity Pathway Analysis (IPA) to determine active canonical pathways based on proportions of pathway-specific genes also upregulated^53^. To generate Supplementary Figure 15, L-R pairs of all expressed genes from integrated MAR21 and SEP21 tissues were calculated.

### Spatial proteomics

A serial tissue section (4-μm thick) from the MAR21 FFPE block was taken and analyzed using PhenoCycler. Coverslip preparation, antibody conjugation, tissue staining, PhenoCycler rendering, and imaging were completed in accordance with PhenoCycler manufacturer instructions (Akoya Biosciences User Manual, Revision-C)^54^. Antibodies used for tissue staining and their respective targets are anti-CD20-BX007, anti-PANCK-BX019, anti-CD8*α*-BX026, anti-Ki67-BX047, anti-CD45RO-BX017, anti-CD3ε-BX045, anti-CD107a-BX006, anti-HLA-DR-BX033, anti-CD4-BX003, anti-CD68-BX015 and anti-CD45-BX021. Probe addition and washing/denaturing steps were performed using the PhenoCycler CIM software version 1.30.0.12. Images were collected with the 20x objective (0.8 NA) Zeiss Axio Observer and processed using the Zen Blue v3.2 software.

### Pre-processing of PhenoCycler data

The raw PhenoCycler data was processed via QuPath software (version 0.3.2)^55^. Cells were segmented by using the QuPath function cell detection on PhenoCycler DAPI channel with default parameters (minAreaMicrons=2.0, MaxAreaMicrons=500.0, watershedPostProcess=True, smoothBoundaries=True, and threshold=2.0). The protein expression intensity was then measured for each segmented cells and exported for further QC. The raw protein expression intensity matrices were filtered by quantile. Cells with total counts lower than 0.05 quantile or higher than 0.95 quantile were discarded to remove the outliers. Filtered matrices were then transformed into logarithmic scale for downstream analysis.

### PhenoCycler cell type annotation

PhenoCycler cells were annotated based on the expression of the protein markers. In total, 17 cell types were identified based on the combination of 11 key immune and cancer markers, including tumor (PANCK), tumor infiltrating cells (CD45), Dividing tumor (KI67, PANCK), Dividing macrophage (KI67, CD68), Dividing CD8 T cells (KI67, CD45, CD3ε, CD8), Dividing CD4 T cells (KI67, CD45, CD3ε, CD4), Antigen-presenting cells (CD45, HLA-DR), T-cell (CD45, CD3ε), B-cell (CD45, CD20), Activated B cells (CD45, CD20, HLA-DR), CD8 T cells (CD45, CD3ε, CD8), CD4 T cells (CD45, CD3ε, CD4), Activated CD8 T cells (CD45, CD3ε, CD8, CD107a), Activated CD4 T cells (CD45, CD3ε, CD4, CD107a), Memory CD8 T cells (CD45, CD3ε, CD8, CD45RO), Memory CD4 T cells (CD45, CD3ε, CD4, CD45RO) and Macrophage/monocyte (CD45, CD68). For a given cell *j*, the cell type *C*_*j*_ can be defined by the protein marker subset which gives the largest geometric mean value as:

1 $${C}_{j}={\arg }\mathop{\max }\limits_{C}\mathop{\prod}\limits_{i=1}^{n}{x}_{i}^{C}$$

where ${x}_{i}^{C}$ is the expression value of protein marker *i* that belongs to cell type *C*.

### Image-based integrative analysis of PhenoCycler and Visium data

The Python package SimpleITK was used to perform image registration^56^. PhenoCycler images were first downscaled to an appropriate resolution to match the resolution of the corresponding Visium histological image. The DAPI channel in the PhenoCycler image was cropped and rotated to have the same capture area and orientation as the Visium histological image and was used as the moving image (query image). Visium histological images were converted to grayscale images to transform the pixel data dimension consistent with the PhenoCycler DAPI channel image and were used as the fixed image (target/reference image). After centralizing the two images, the rigid affine transformation was applied for shearing, shifting, and scaling the moving image to align with the fixed image in lower resolution as the initial step. Finally, the non-rigid B-spline transformation was applied on affine initialization to refine the local alignment. The mutual information was used as the evaluation matrix to optimize the parameter for both affine and b-spline transformation.

### SPiCi—Spatial Proteomics informed ST spot Cell identification method (for PhenoCycler and Visium data)

After registering the PhenoCycler DAPI image to Visium histological image, the optimized transformation matrix was then used to map the cells from the original PhenoCycler spatial coordinates (x, y) to newly mapped spatial coordinates (x’, y’) that belong to the original Visium spatial coordinate reference. With this shared coordinate system, cells in PhenoCycler data can be grouped by the Visium spatial radius (d = 55 μm, a diameter equivalent to a Visium spot size) using the transferred spatial coordinates (x’, y’) and the coordinate of the Visium spot (x, y). The mapping is based on the assumption that cell type composition at the two corresponding spots in two adjacent tissue section remain mostly similar, even when the cells are not identical. In the integrated dataset, each Visium spot contains thousands of gene expression profiles and all the protein measured in the PhusionCycler data. As the result, the PhusionCycler-defined cells are mapped to Visium spots and the mapped cells were then used to approximate the cell type composition of Visium spots. The proportion *P* for each cell type *C* in a particular Visium spot *s* can be defined as:

2 $${P}_{s}^{C}=\frac{{N}_{C}}{{N}_{s}}$$

where the *N*_*s*_ denotes the total number of cells that fall into Visium spot *s* and the total number of cells annotated as cell type *C* is denoted as *N*_*C*_.

### Detection of tumor and lymphocyte spots by deconvolution approaches and SPiCi

Visium cell spot deconvolution was performed using five established methods selected to represent all three categories, including (1) referenced-based methods as in *Seurat* Label Transfer^18^ and *RCTD*^19^; (2) spatially informed referenced-based methods as *CARD*^15^, tangram^20^; and (3) reference-free method *STdeconvolve*^16^. Publicly available scRNA-seq count data from the National Center of Biotechnology Information’s Gene Expression Omnibus (GSE181919) was utilized for the reference-based deconvolution methods^17^. This scRNA-seq dataset was generated from 37 HNSCC specimens (mixture of HPV+ and HPV−), with well-annotated cell types. The reference scRNA-seq and the query ST datasets were both normalized using *SCTransform* from *Seurat*^18^ and then used as inputs for the four reference-based methods using their own default parameter setting. Reference-free method *STdeconvolve*^16^ only utilized normalized ST data as input to calculate cell-type proportions across each spot.

All deconvolution methods and *SPiCi* were applied to the MAR21 sample to identify within Visium spots the tumors and Lymphocytes (the primary focus of this study). The dominant cell type with the highest classification probability resulting from each deconvolution method was designated as the inferred cell type. The results of inferred cell types were subsequently compared with the ground truth pathological annotation. Evaluation of performance was performed utilizing accuracy metrics and confusion matrices. The spatial distributions were visualized and cross-compared using the STlearn software.

### Ethics statements

This study was approved by the Metro South Human Research Ethics Committee (approval #HREC/2019/QMS/49990) and The University of Queensland (approval #2019001021) and conducted in accordance with the Declaration of Helsinki. Participants provided written consent after receiving a Participant Information and Consent Form. Written informed consent was obtained from the individuals for the publication of any potentially identifiable images or data included in this article.

### Reporting summary

Further information on research design is available in the Nature Research Reporting Summary linked to this article.

### Supplementary information

SUPPLEMENTAL MATERIAL

Reporting Summary

## Data Availability

This study’s raw sequencing data (fastq and BAM files) is under controlled access and is available upon request. PhenoCycler images and processed Visium gene expression matrices are publicly available and can be freely downloaded from UQ e-space, at 10.48610/698bb9e.

## Code availability

All scripts used in this study are open-source and stored on our GitHub (https://github.com/BiomedicalMachineLearning/HeadAndNeck_DeepCaseStudy).
