# Supplementary material for: Deep spatial-omics analysis of Head & Neck carcinomas provides alternative therapeutic targets and rationale for treatment failure
Source: NPJ Precis Oncol. 2023 Sep 13;7:89. doi: 10.1038/s41698-023-00444-2 (PMC10499928; doi:10.1038/s41698-023-00444-2)
Supplement: Supplementary file 1 — SUPPLEMENTAL MATERIAL [file 41698_2023_444_MOESM1_ESM.pdf]

Supplementary Information

Supplement to: Causer A., Tan X., Lu X. *et al. Deep spatial-omics analysis of Head & Neck carcinomas provides alternative therapeutic targets and rationale for treatment failure*

Supplementary Information Table of Contents

Content			Page
1.	**Supplementary Table 1**	*Spatial proteomics: PhenoCycler antibody panel.*	4
2.	**Supplementary Table 2**	*Spatial proteomics: cell type annotation based on surface protein expression.*	4
3.	**Supplementary Figure 1**	*Flow-diagram representing the implemented workflow.*	5
4.	**Supplementary Figure 2**	*High-resolution H&E images of MAR21 and SEP21 samples.*	6
5.	**Supplementary Figure 3**	*UMAP representation of unbiased Visium clustering of MAR21 and healthy paired samples.*	7
6.	**Supplementary Figure 4**	Distinct transcriptional profiles of inner core and leading-edge clusters.	7
7.	**Supplementary Figure 5**	*Spatial transcriptomics (Phenocycler)-informed cell identification (SPiCi) and established transcription-based deconvolution methods.*	8
8.	**Supplementary Figure 6**	*Integrated Spatial-Omic characterization of tumor immune cell microenvironments.*	9
9.	**Supplementary Figure 7**	*Comparison of deconvolution methods and SPiCi results with pathologist annotation.*	10
10.	**Supplementary Figure 8**	*Localization of proliferating tumor cells.*	11
11.	**Supplementary Figure 9**	*Differential analysis of transcriptional profiles of tumor clusters within MAR21 OPSCC.*	12
12.	**Supplementary Figure 10**	*PD-L1 and PD-1 protein detection.*	13
13.	**Supplementary Figure 11**	*Visium data integration and batch correction of tumor tissues.*	13
14.	**Supplementary Figure 12**	*Tumor transcriptional profile recapitulated in recurrent SEP21 OPSCC.*	14
15.	**Supplementary Figure 13**	*Initial Pathologist annotation of SEP21 OPSCC sample.*	15
16.	**Supplementary Figure 14**	*Differential expression analysis of tumor clusters 4 and 7 in the MAR21-SEP21 OPSCC integrated dataset.*	15
17.	**Supplementary Figure 15**	*Ligand-receptor interaction analysis in MAR21 and SEP21 tissues.*	16

**18.**	**Supplementary Figure 16**	*Transcriptional comparison between MAR21 and recurrent SEP21 and ligand-receptor interaction analysis for therapeutic target selection.*	17
**19.**	**Supplementary Figure 17**	*Top druggable targets differ between additional OPSCC patients.*	18
**20.**	**Supplementary Table 3**	*Gene classification based on function reported in the literature in the cancer setting.*	19
**21.**	**Supplementary Table 4**	*Identified clinical and preclinical targets.*	26
**22.**	**Supplementary References**		46

1. Supplementary Table 1

AKOYA Antibody	AKOYA Tag number	AKOYA PIN	Target cell
CD20	BX007	4450018	B and T cell subsets
PanCK	BX019	4450020	Epithelial cells
CD8a	BX026	4250012	T cell subsets, NK subset
Ki67	BX047	4250019	Proliferating cells
CD45Ro	BX017	4250023	Activated and memory T cells, B cell subsets
CD3e	BX045	4450030	T cells, NK-T cells, thymocytes
CD107a	BX006	4350001	Degranulation marker
HLA-DR	BX033	4450029	Activated B cells, antigen presenting cells
CD4	BX003	4350018	T cell subsets, monocytes, macrophages
CD68	BX015	4350019	Monocytes, macrophages
CD45	CX021	4450042	Hematopoietic cells
PD-1	RX046	4550038	Activation/exhaustion
PD-L1	BX043	4550072	Inhibitory signal

Supplementary table 1. **Spatial proteomics: PhenoCycler antibody panel.**

2. Supplementary Table 2

Cell Type	Marker Proteins
Tumor	PANCK
Tumor infiltrating cell	CD45
Dividing Tumor	PANCK, Ki67
Antigen presenting cell	CD45, HLA-DR
T cell	CD45, Cd3ε
B cell	CD45, CD20
Activated B cell	CD45, CD20, CD8
CD8 T cell	CD45, Cd3ε, CD8
CD4 T cell	CD45, Cd3ε, CD4
Activated CD8 T cell	CD45, Cd3ε, CD8, CD107a
Activated CD4 T cell	CD45, Cd3ε, CD4, CD107a
Memory CD8 T cell	CD45, Cd3ε, CD8, CD45R0
Memory CD4 T cell	CD45, Cd3ε, CD4, CD45R0
Dividing CD8 T cell	CD45, Cd3ε, CD8, Ki67
Dividing CD4 T cell	CD45, Cd3ε, CD4, Ki67
Macrophage/Monocyte	CD45, CD68
Dividing Macrophage	CD45, CD68, Ki67

Supplementary table 2. **Spatial proteomics: cell type annotation based on surface protein expression.**

3. Supplementary Figure 1

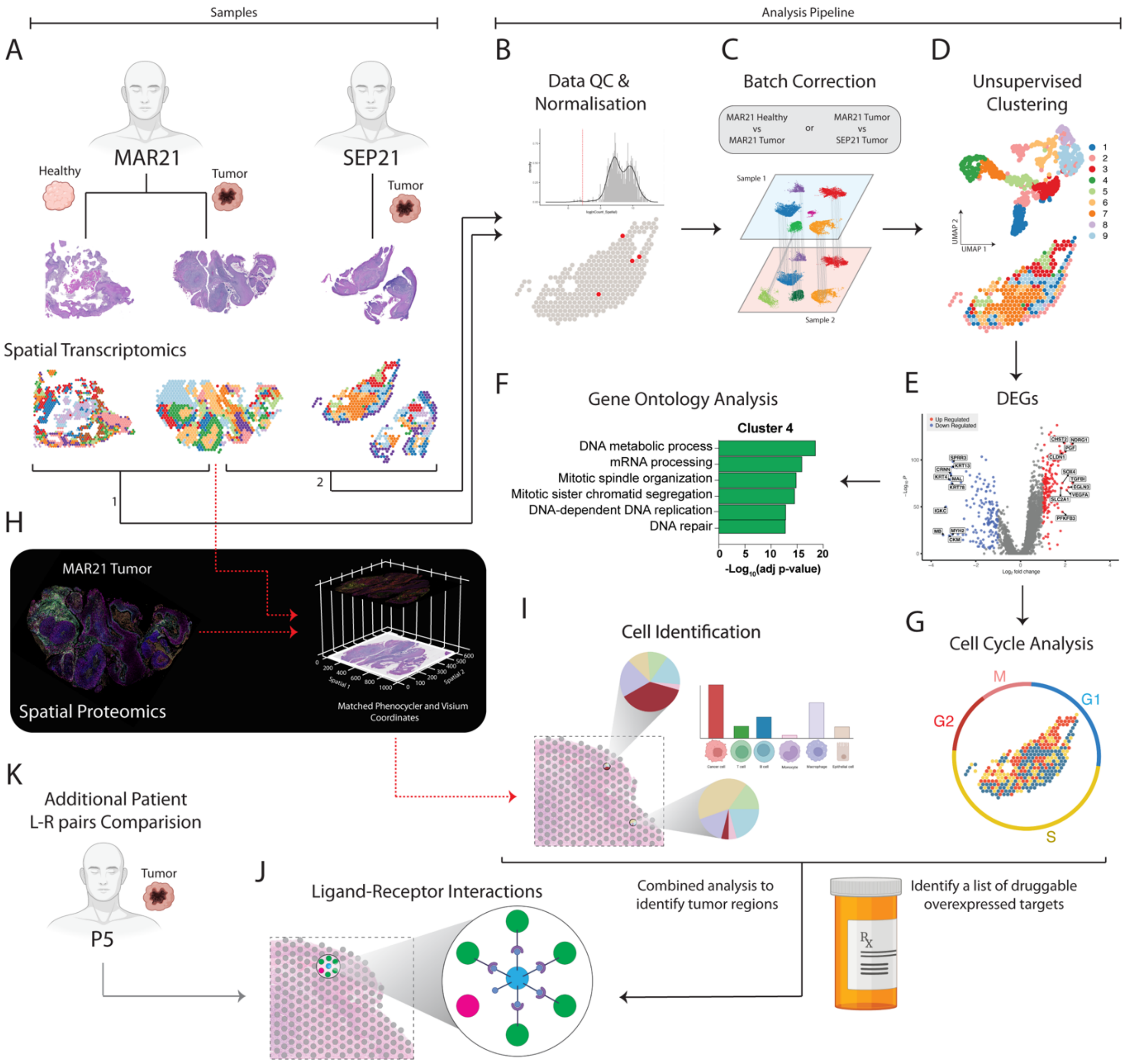

Supplementary Figure 1. **Flow-diagram representing the implemented workflow. A.** Displays the different samples being analyzed within the study. Two comparisons are performed: 1) between healthy and tumor samples of MAR21 sample. 2) between MAR21 and SEP21 tumor samples. Both comparisons undergo analysis through the same pipeline which includes: **B.** Data quality control and normalization, **C.** Batch correction between samples, **D.** Unsupervised clustering to identify spots of similar transcriptomic profiles. **E.** Differential gene expression analysis, **F.** Gene ontology term enrichment analysis, and **G.** Cell cycle state analysis. **H.** In addition to spatial transcriptomics, spatial proteomics was performed on the MAR21 tumor sample. Integration of spatial transcriptomics and proteomics was performed to inform **I.** Spot cell composition. The overall analysis was used to identify and analyze the tumor regions within the samples. **J.** Ligand-receptor (L-R) interaction analysis was then performed to identify the most active L-R pairs which are druggable targets. **K.** An additional patient was analyzed in combination to assess if the L-R pairs are patient-specific. **L.** L-R pair activity was further assessed through pathway analysis of differentially expressed genes. **M.** The collective findings were then used to determine the top druggable targets specific to the patient.

4. Supplementary Figure 2

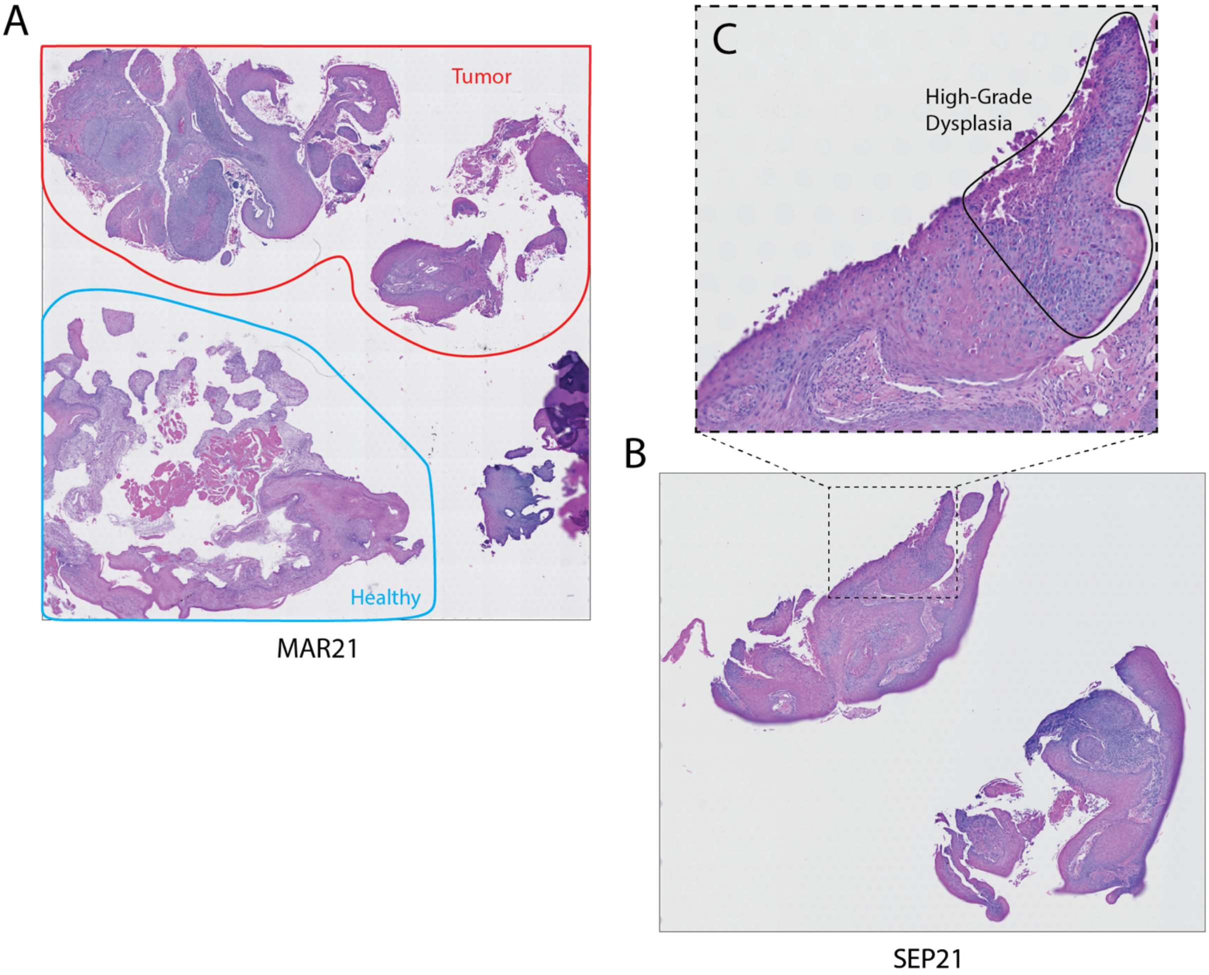

Supplementary Figure 2. **High-resolution H&E images of MAR21 and SEP21 samples. A.** Displays the high-resolution H&E image of both the healthy and tumor MAR21 sample. **B.** The high-resolution H&E image of SEP21 tumor samples. C. The highlighted box represents the tissue region which pathologists identified as high-grade dysplasia after re-analysis of the original H&E image. The raw images have been made publicly accessible at https://doi.org/10.48610/698bb9e.

5. Supplementary Figure 3

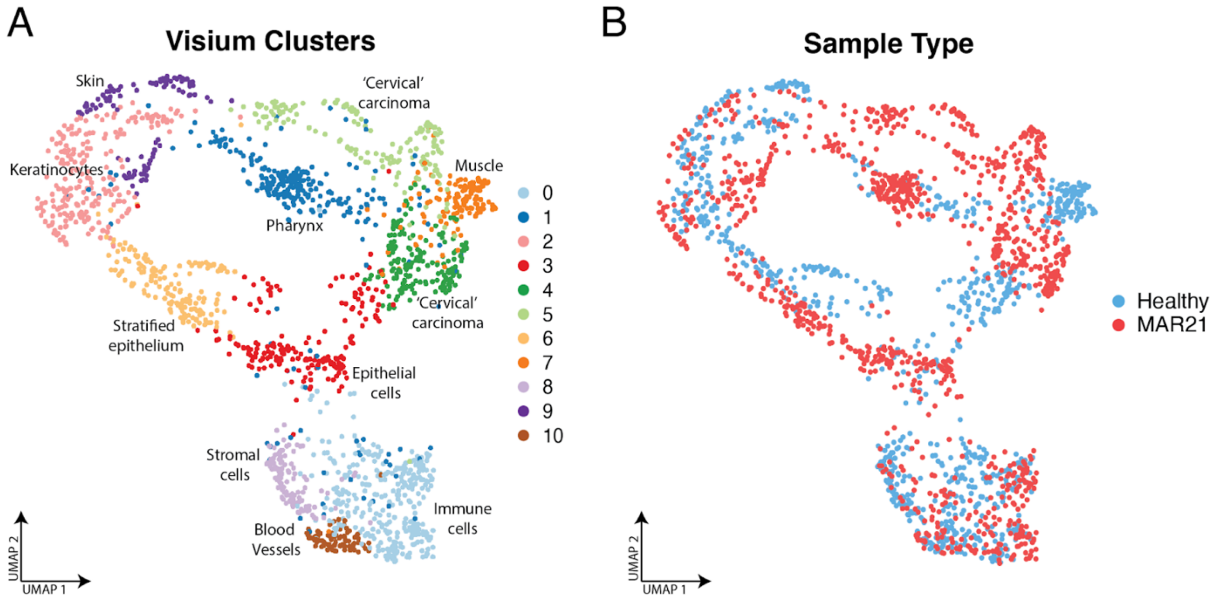

Supplementary Figure 3. **UMAP representation of unbiased Visium clustering of MAR21 and healthy paired samples**. UMAP of clusters highlights distinction and separation between populations. **A.** Cluster annotations were generated by comparing differentially overexpressed genes with a reference database (JENSENs Tissues; EnrichR). **B.** Sample type UMAP displays batch correction between healthy (blue) and MAR21 OPSCC (red) samples.

6. Supplementary Figure 4

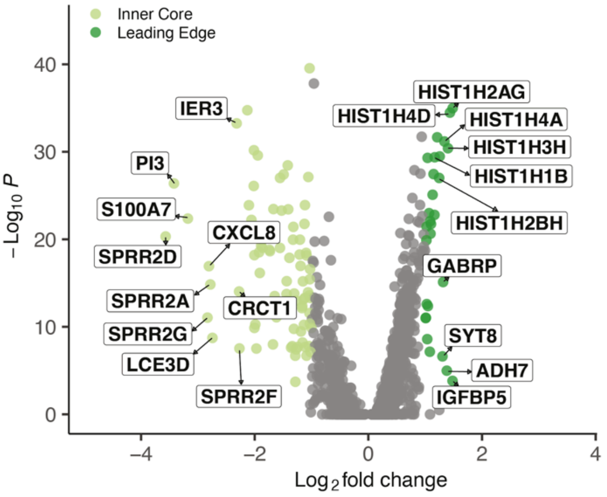

Supplementary Figure 4. **Distinct transcriptional profiles of inner core and leading-edge clusters.** DEGs between newly defined Visium clusters 4 (dark green) and 5 (light green). Significant DEGs with Wilcoxon test p-value < 0.001 and fold-change >1 are colored based on cluster.

7. **Supplementary Figure 5**

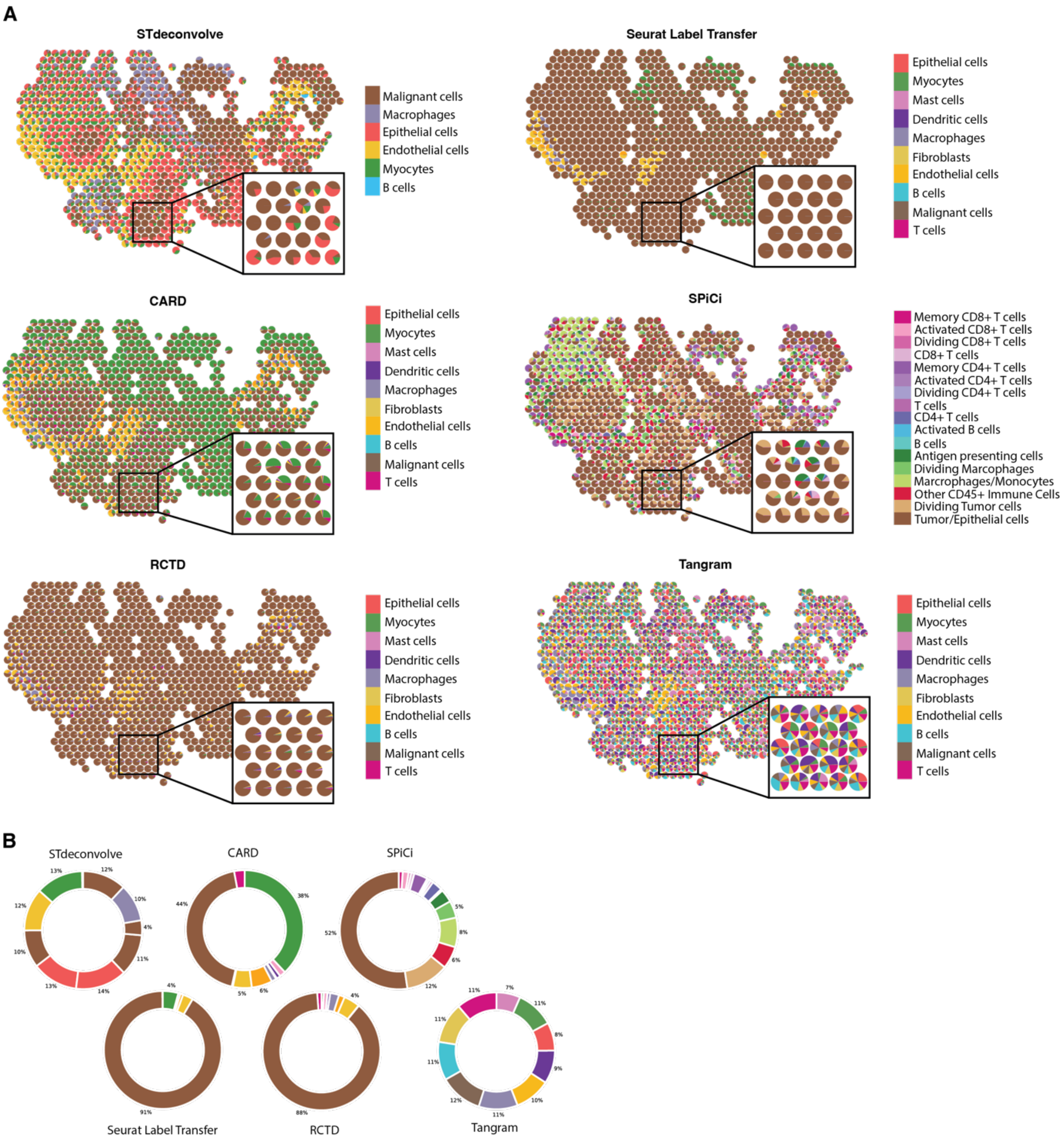

Supplementary Figure 5. **Spatial transcriptomics (Phenocycler)-informed cell identification (*SPiCi*) and established transcription-based deconvolution methods. A.** Displays spot cell annotation of tumor sample using each algorithm. Spots are represented as pie graphs denoting the proportions of each cell type identified within each spot capture region. Enlarged regions encapsulate the outer proliferating ring of the tumor and the immune cell infiltrated core. **B.** Overall percentages of different cell types identified by each approach (across all spots combined).

8. Supplementary Figure 6

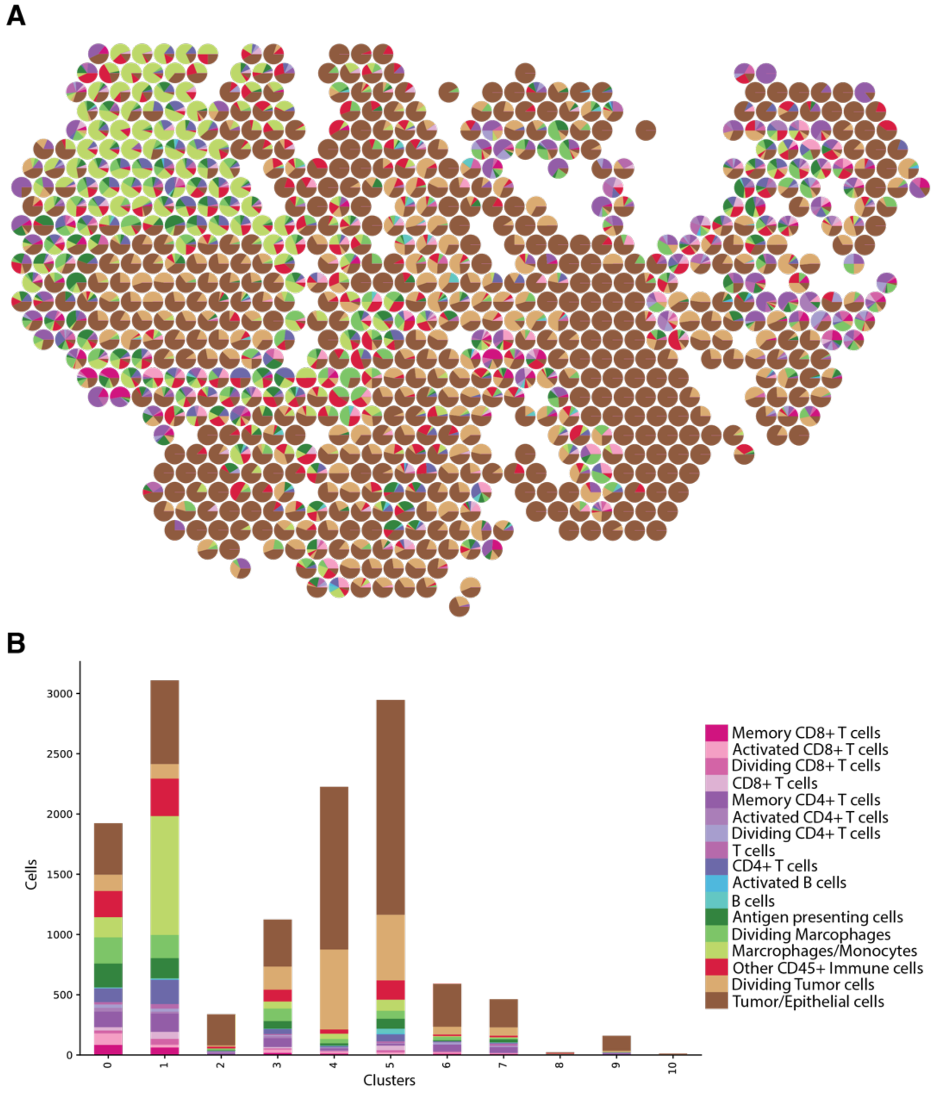

Supplementary Figure 6. **Integrated Spatial-Omic characterization of tumor immune cell microenvironments. A.** Visium spot cell identification of MAR21 OPSCC using *SPiCi*. Spots are presented as pie-charts representing the relative proportions of cell types located within each spot. **B.** Quantification of cell-types found within each Visium cluster.

9. Supplementary Figure 7

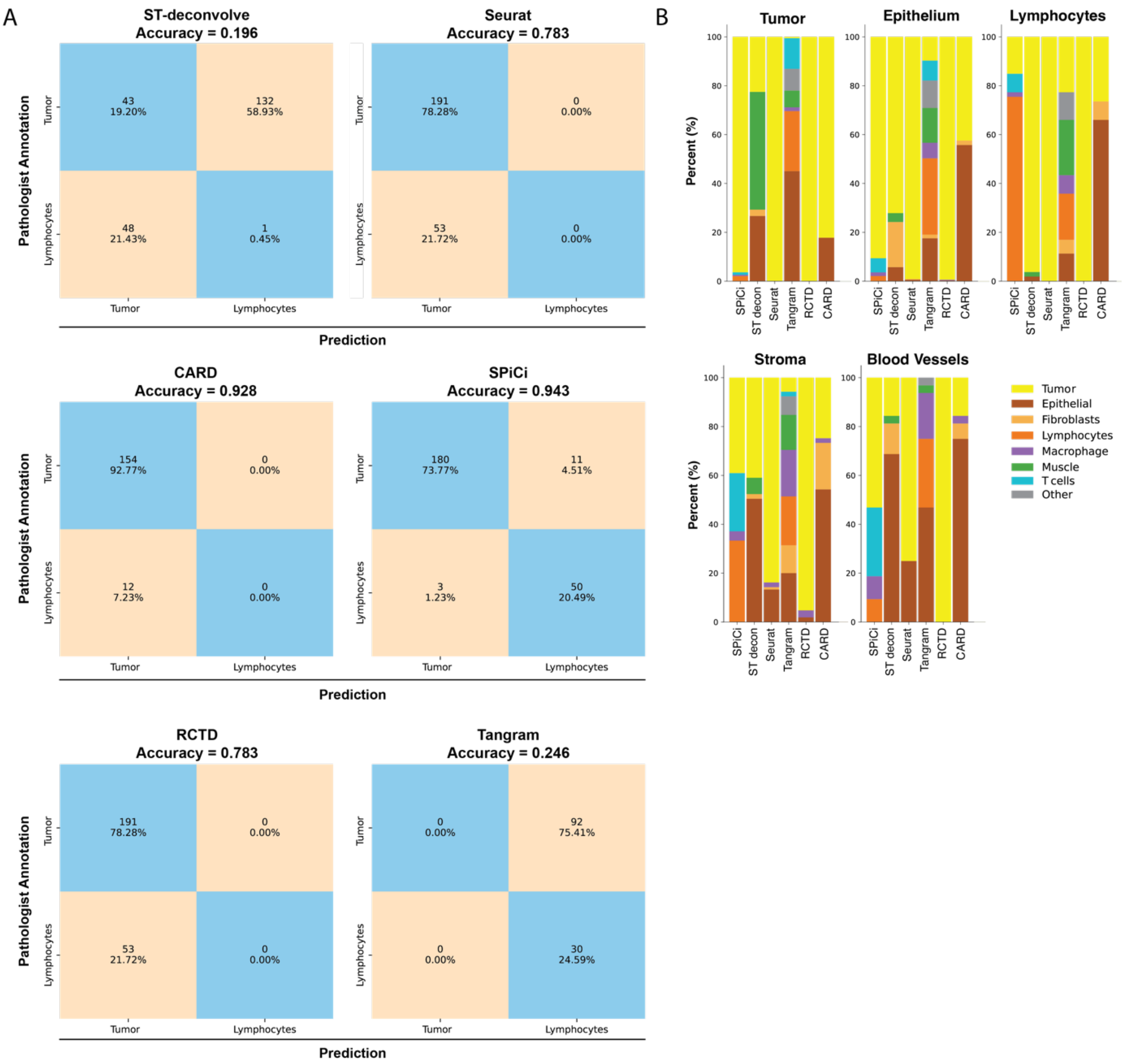

Supplementary Figure 7. **Comparison of deconvolution methods and *SPiCi* results with pathologist annotation. A.** Confusion matrices were calculated to evaluate the performance of each method at detecting tumor and lymphocyte cells compared to the ground truth pathological annotation. The cell type label for each spot is determined by the cell type with the highest proportion (i.e., classification probability) for that spot, calculated by each method. The inferred spot labels were then compared with the ground truth pathological annotations. Accuracy rates were calculated based on results generated from each confusion matrix. **B.** Bar graphs indicate the proportion of various cell types predicted by each method with respect to each cell type identified by pathological annotation.

10. Supplementary Figure 8

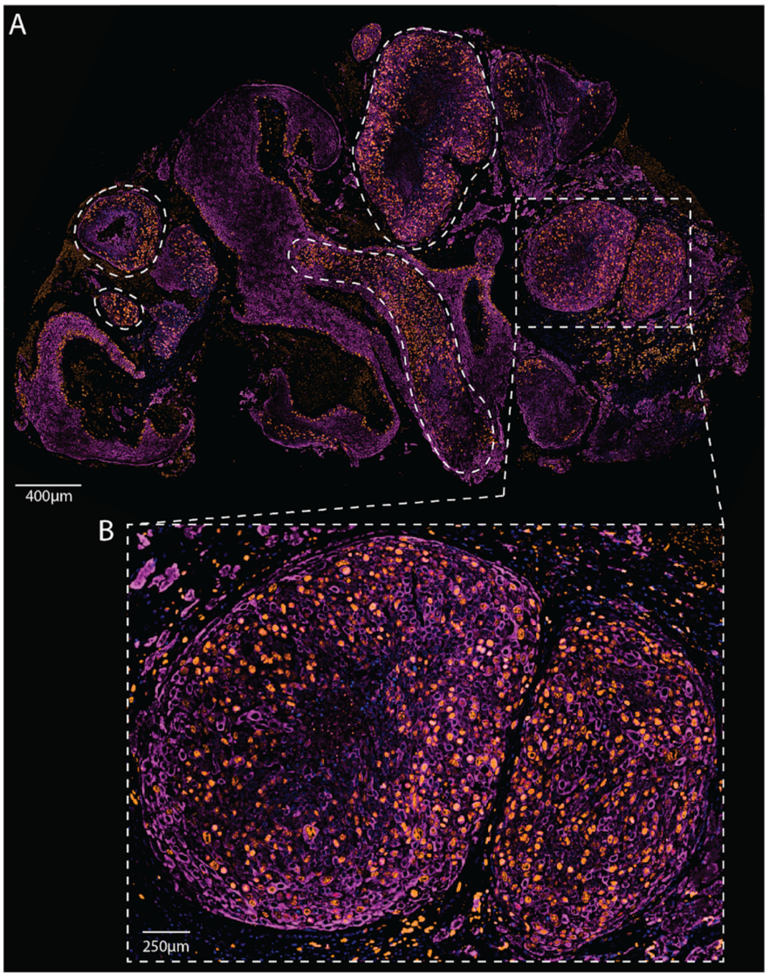

Supplementary Figure 8. **Localization of proliferating tumor cells. A.** PhenoCycler fluorescence images displaying PanCK+ (Purple) and Ki67+ (Orange) cells across the MAR21 OPSCC sample. **B.** Tumor region of interest presents with leading edge of active proliferating cells and inactive tumor core.

11. Supplementary Figure 9

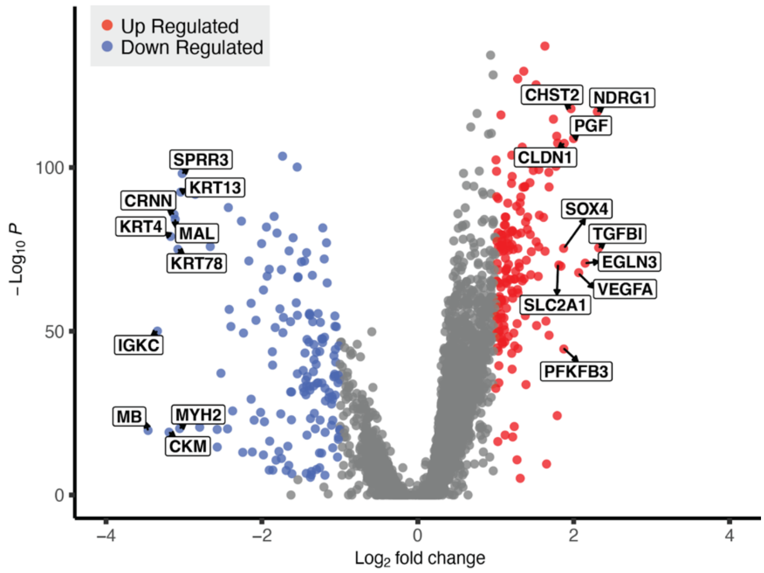

Supplementary Figure 9. **Differential analysis of transcriptional profiles of tumor clusters within MAR21 OPSCC.** DEGs of the combined tumor clusters relative to all other clusters. Significantly up-regulated and down-regulated genes are highlighted in red and blue respectively (Wilcoxon test p-value < 0.001 and an absolute fold- change > 1). The top 10 over- and under-expressed genes are annotated.

12. Supplementary Figure 10

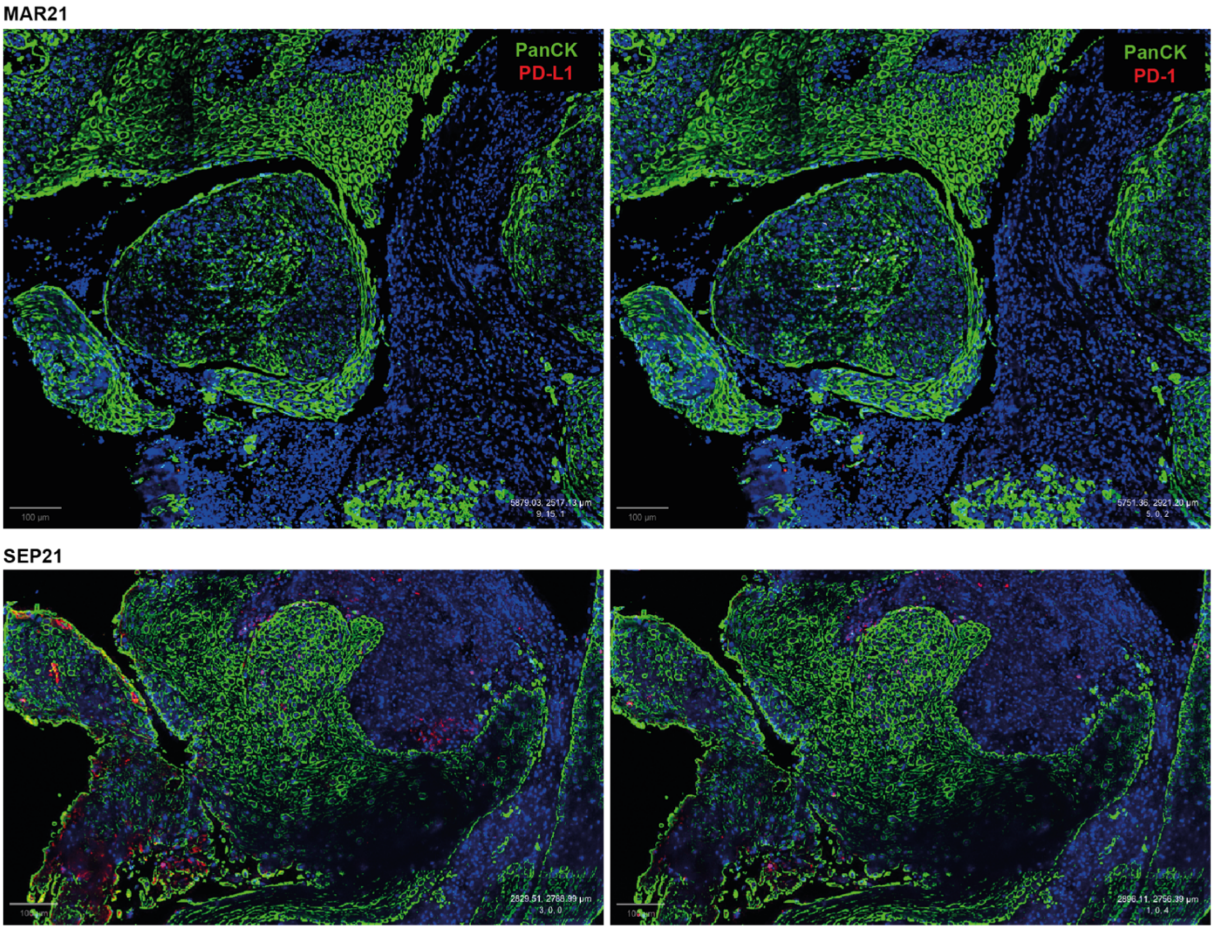

Supplementary Figure 10. **PD-L1 and PD-1 protein staining. A.** PHENOCYCLER fluorescence images displaying PanCK+ (green) and PD-L1 (red, *left*) or PD-1 (red, *right*) cells across the MAR21 and SEP21 OPSCC samples.

13. Supplementary Figure 11

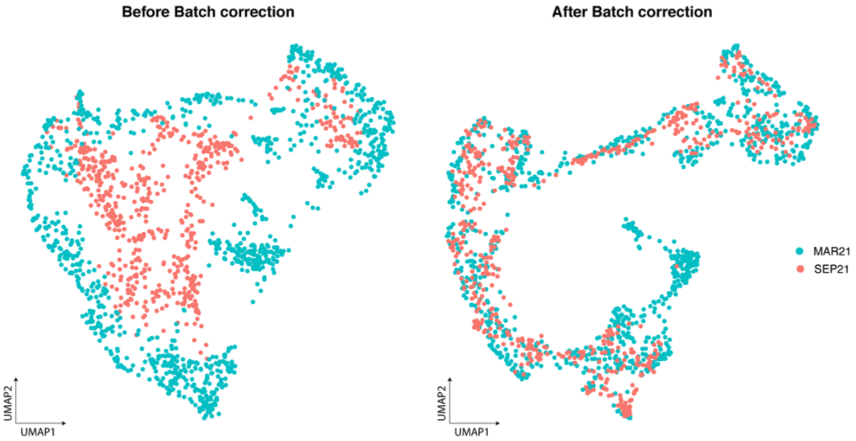

Supplementary Figure 11. **Visium data integration and batch correction of tumor tissues.** Spatial transcriptomic data generated from MAR21 and SEP21 tumors was integrated (*left)* and batch effect corrected (*right*) using the Seurat anchor integration pipeline.

14. Supplementary Figure 12

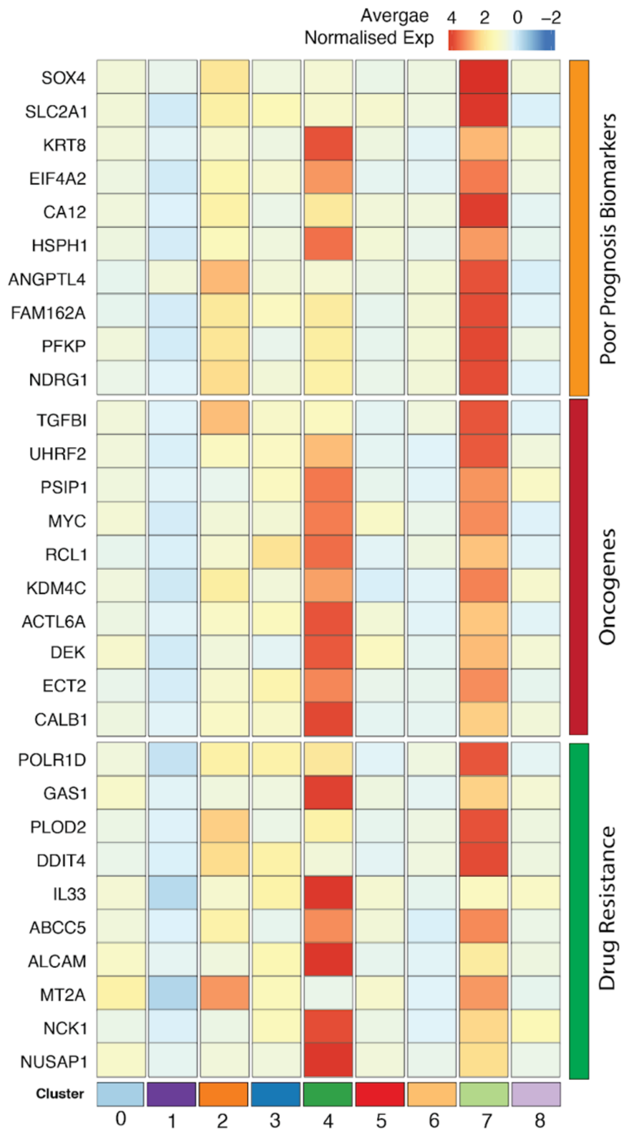

Supplementary Figure 12. **Tumor transcriptional profile recapitulated in recurrent SEP21 OPSCC.** Relative expression of poor prognosis markers (red), oncogenes (orange) and drug resistance genes (green), previously identified from the combined tumor cluster within MAR21, across each newly defined cluster generated through comparing OPSCC samples. The top 9 genes of each category were displayed. Colors gradient represents average normalized expression values across all spots in each cluster, which were z-transformed by genes (rows of the heatmap).

15. Supplementary Figure 13

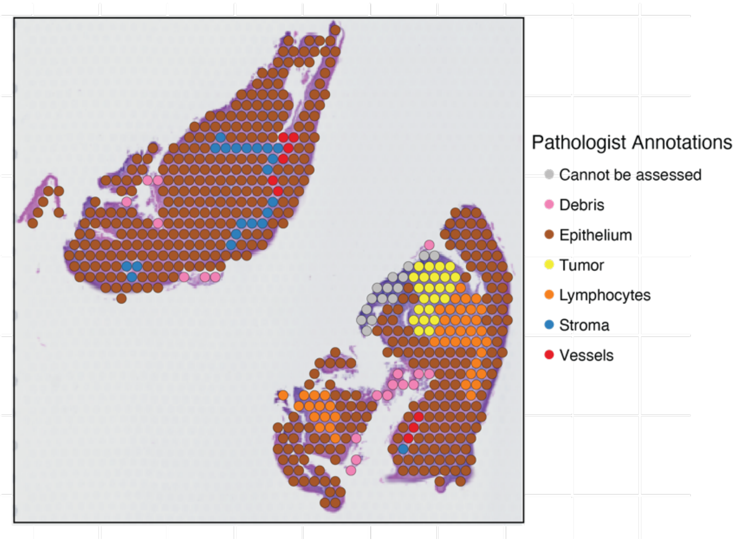

Supplementary Figure 13. **Initial Pathologist annotation of SEP21 OPSCC sample.** Colored spots identify different morphological tissue structure identified from the H&E image by a clinical pathologist.

16. Supplementary Figure 14

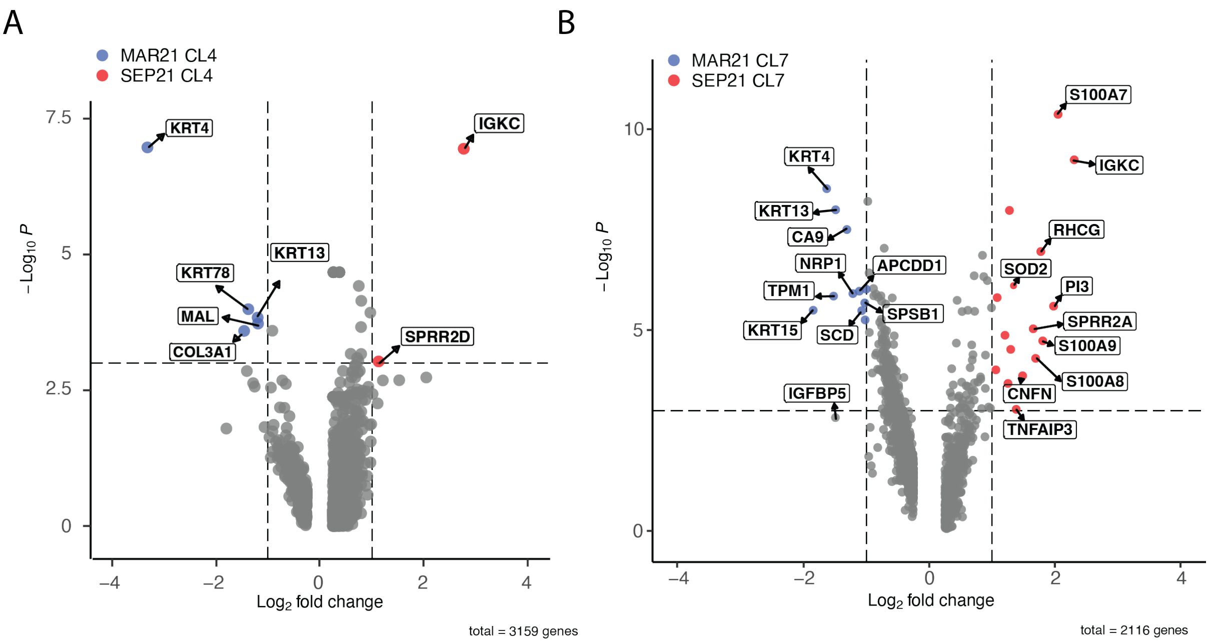

Supplementary Figure 14. **Differential expression analysis of tumor clusters 4 and 7 in the MAR21-SEP21 OPSCC integrated dataset.** DEGs of the tumor clusters CL4 (A) and CL7 (B) from MAR21 and SEP21 samples. Significantly up-regulated and down-regulated genes are highlighted in red and blue respectively (Wilcoxon test p-value < 0.001 and an absolute fold-change > 1). The top over- and under-expressed genes based on fold change and P-values are annotated.

17. Supplementary Figure 15

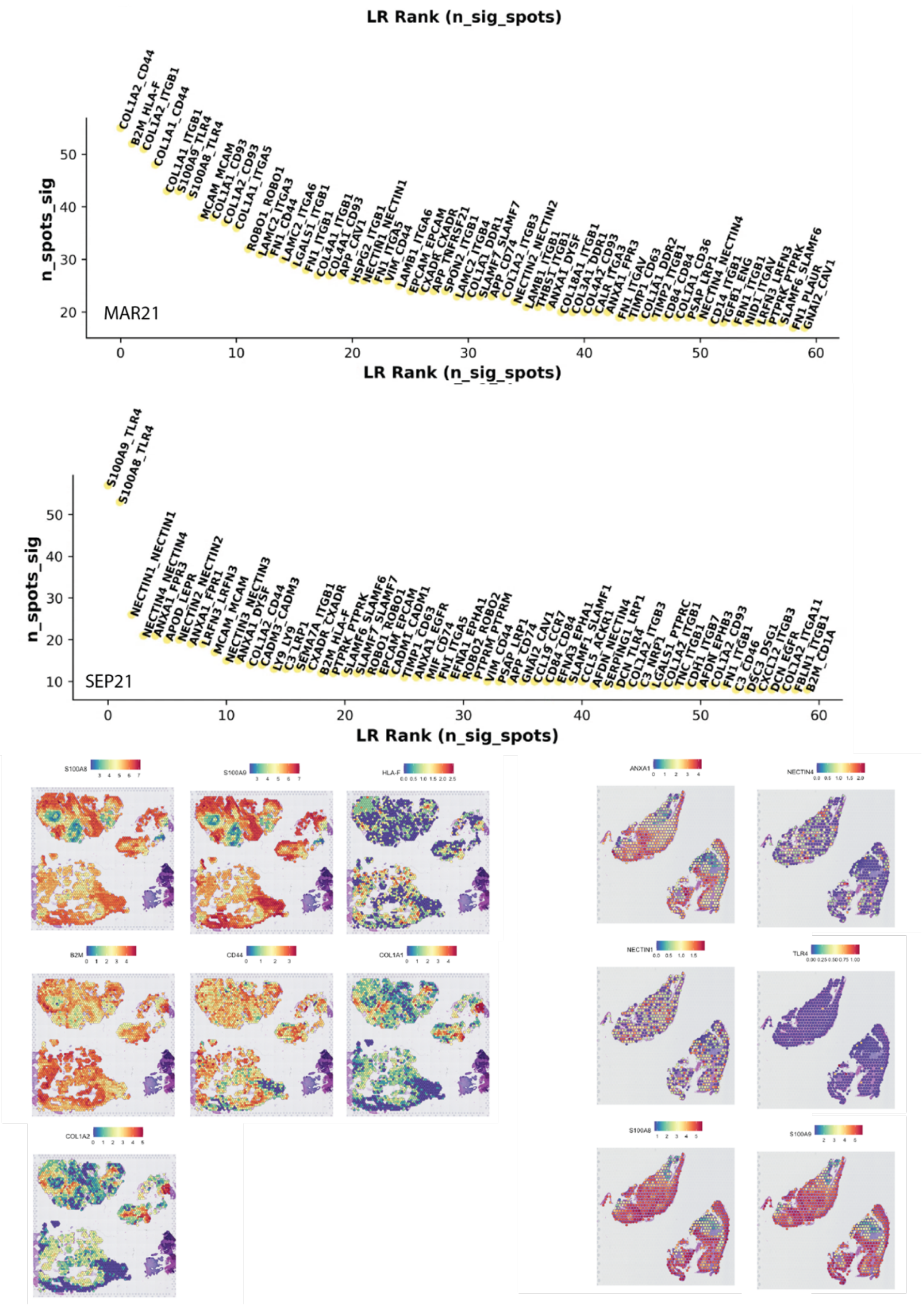

Supplementary Figure 15. **Ligand-receptor interaction analysis in MAR21 and SEP21 tissues. A.** Ranking of top 60 Ligand/Receptor (L/R) expressed by the MAR21 (*top*) and recurrent SEP21 (bottom). Rank was based on the number of spots with significant interactions of each L/R pair across each sample. **B.** Spatial expression of top L/R pairs expressed by MAR21(*left*) and SEP21(*right*). In all feature plots, the relative gene expression per spot is indicated by the coloured scale bars.

18. Supplementary Figure 16

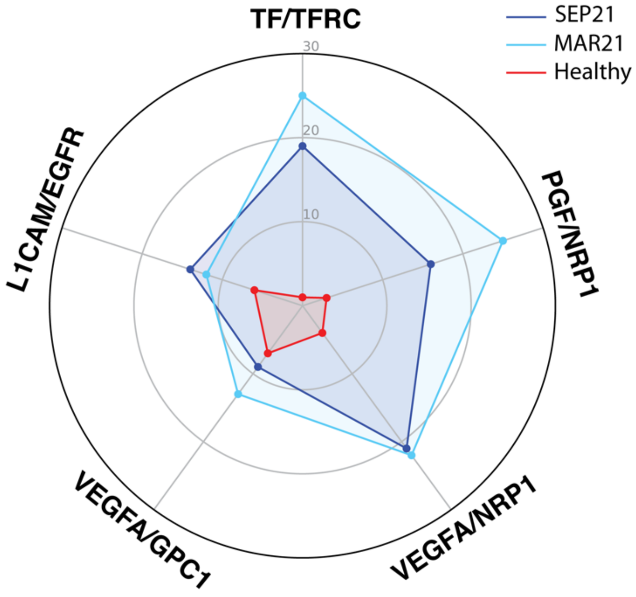

Supplementary Figure 16. **Transcriptional comparison between MAR21 and recurrent SEP21 and ligand-receptor interaction analysis for therapeutic target selection.** Spider-plot represents the top 5 most active L-R pairs found across tumor tissue based on the number of spots with significant interactions using the L-R pairs. Red line, healthy tissue; dark blue, SEP21; light blue, MAR21.

19. Supplementary Figure 17

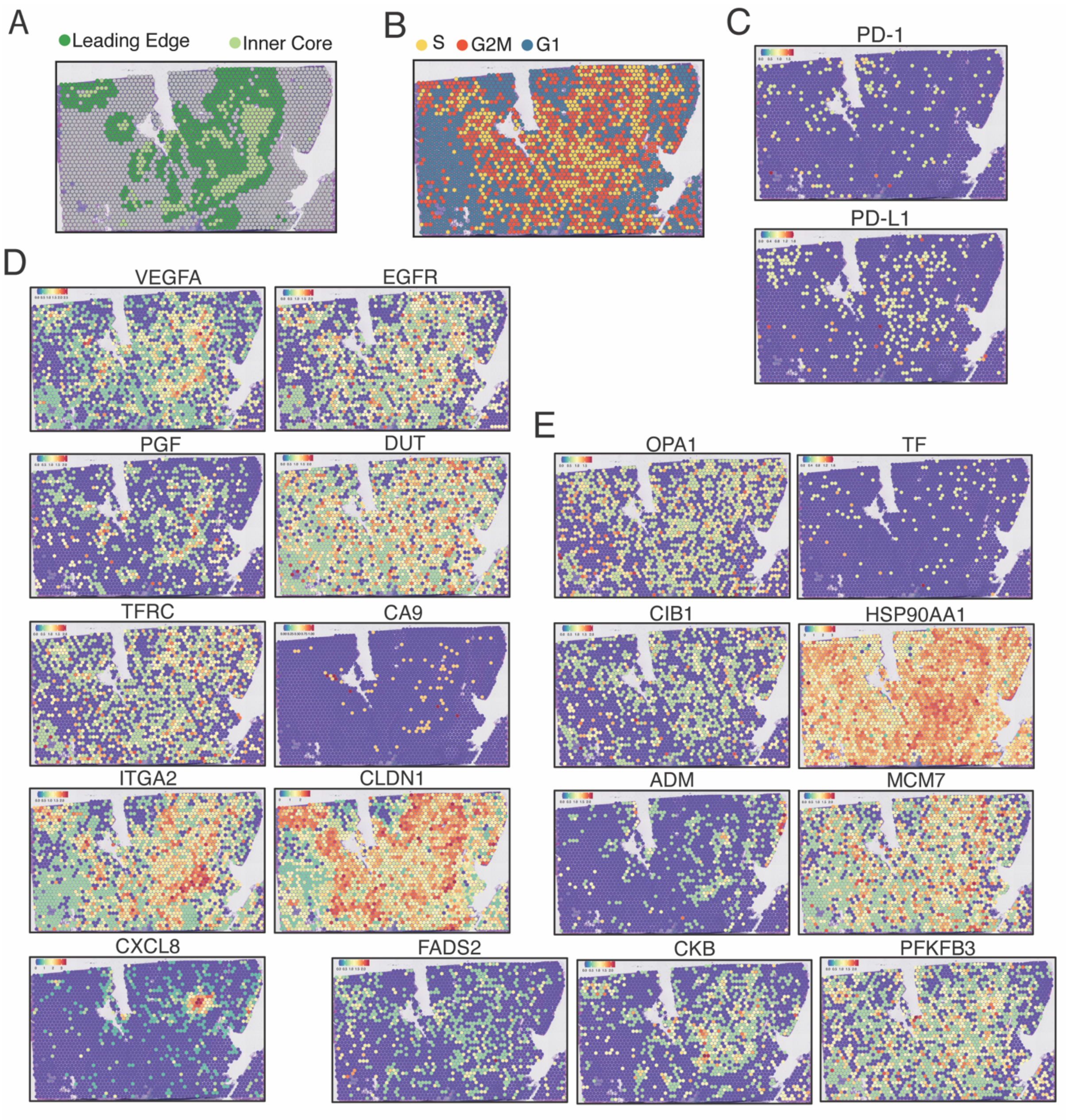

Supplementary Figure 17. **Top druggable targets differ between additional OPSCC patients. A.** Spatial representation of Visium clusters identified as tumor leading edge (dark green) and inner core (light green) within an additional OPSCC patient. **B.** Cell cycle phase of each spot based on relative expression of specific cell phase genes. **C.** Spatial expression of genes targeted by Nivolumab and Pembrolizumab targeted *PD-1/PD-L1* pathway. **D.** Spatial visualization of gene expression of genes targeted by previously identified clinical therapies. **E.** Spatial localization of gene expression levels for select experimental drug therapies. In all feature plots, the relative gene expression per spot is indicated by the coloured scale bars.

Supplementary table 3. **Gene classification based on function reported in the literature in the cancer setting.**

Gene	Categories	Ref
HAUS6	Oncogenes	[1]
DBN1	Oncogenes	[2-4]
CCNL1	Oncogenes	[5]
ACTL6A	Oncogenes	[6]
CHAF1A	Oncogenes	[7,8]
DEK	Oncogenes	[9]
ECT2	Oncogenes	[10,11]
EIF4EBP1	Oncogenes	[12]
FXR1	Oncogenes	[13]
JUN	Oncogenes	[14]
KDM4C	Oncogenes	[15]
MYBL2	Oncogenes	[16]
MYC	Oncogenes	[17]
NSD2	Oncogenes	[18]
PFDN2	Oncogenes	[19]
PSIP1	Oncogenes	[20]
RNF19A	Oncogenes	[21]
TAGLN2	Oncogenes	[22,23]
TGFBI	Oncogenes	[24]
UHRF2	Oncogenes	[25]
VAV3	Oncogenes	[26,27]
JAG1	Oncogenes	[28,29]
CENPW	Oncogenes	[30,31]
SMC4	Good and Poor prognosis biomarker	[32,33]
COL7A1	Good prognosis biomarker	[34]

CXADR	Good prognosis biomarker	[35]
FOXRED2	Good prognosis biomarker	[36]
LPAR3	Good prognosis biomarker	[37]
MEI1	Good prognosis biomarker	[38]
NEFH	Good prognosis biomarker	[39]
VLDLR	Good prognosis biomarker	[40]
PGK1	Poor prognosis biomarker	[41]
ANGPTL4	Poor prognosis biomarker	[42]
ANP32E	Poor prognosis biomarker	[43]
ATP13A3	Poor prognosis biomarker	[44]
BHLHE40	Poor prognosis biomarker	[45,46]
CA12	Poor prognosis biomarker	[47]
CALB1	Poor prognosis biomarker	[48]
CCT3	Poor prognosis biomarker	[49-51]
CENPJ	Poor prognosis biomarker	[52]
CPA4	Poor prognosis biomarker	[53]
CSE1L	Poor prognosis biomarker	[54]
DSC3	Poor prognosis biomarker	[55]
DVL3	Poor prognosis biomarker	[56]
EIF4A2	Poor prognosis biomarker	[57]
FAM162A	Poor prognosis biomarker	[58]
FAT2	Poor prognosis biomarker	[59]
GTF3A	Poor prognosis biomarker	[60]
HILPDA	Poor prognosis biomarker	[61]
HSPA1A	Poor prognosis biomarker	[62]
HSPA1B	Poor prognosis biomarker	[62]
HSPE1	Poor prognosis biomarker	[63]

HSPH1	Poor prognosis biomarker	[64]
IER3	Poor prognosis biomarker	[65]
KRT8	Poor prognosis biomarker	[66]
LAMA5	Poor prognosis biomarker	[67,68]
LPCAT1	Poor prognosis biomarker	[69,70]
NFIL3	Poor prognosis biomarker	[71]
NRARP	Poor prognosis biomarker	[72,73]
NUP58	Poor prognosis biomarker	[74]
NXPH4	Poor prognosis biomarker	[75]
PCLAF	Poor prognosis biomarker	[76]
PFKP	Poor prognosis biomarker	[77]
PLAUR	Poor prognosis biomarker	[78]
PLPP2	Poor prognosis biomarker	[79]
PNCK	Poor prognosis biomarker	[80]
PODXL2	Poor prognosis biomarker	[81]
PSMD2	Poor prognosis biomarker	[82]
PTHLH	Poor prognosis biomarker	[83]
PUM3	Poor prognosis biomarker	[84]
RFC3	Poor prognosis biomarker	[85]
RFC4	Poor prognosis biomarker	[86]
RHOV	Poor prognosis biomarker	[21]
RUVBL1	Poor prognosis biomarker	[87]
SAT1	Poor prognosis biomarker	[88]
SLC2A1	Poor prognosis biomarker	[89]
SNAI2	Poor prognosis biomarker	[90]
SOD2	Poor prognosis biomarker	[91,92]
SOX4	Poor prognosis biomarker	[93]

STC2	Poor prognosis biomarker	[94,95]
TBL1XR1	Poor prognosis biomarker	[96]
TCN1	Poor prognosis biomarker	[97]
TCP1	Poor prognosis biomarker	[98]
TFDP1	Poor prognosis biomarker	[99]
TMEM189	Poor prognosis biomarker	[100,101]
U2SURP	Poor prognosis biomarker	[102]
UBE2C	Poor prognosis biomarker	[26]
YEATS2	Poor prognosis biomarker	[103]
ABCC5	Drug Resistance	[104]
ALCAM	Drug Resistance	[105]
CALCRL	Drug Resistance	[106,107]
FBXO42	Drug Resistance	[108]
GAS1	Drug Resistance	[109]
IL33	Drug Resistance	[110]
MKNK2	Drug Resistance	[111]
MT2A	Drug Resistance	[112,113]
NCK1	Drug Resistance	[114]
NUSAP1	Drug Resistance	[115]
PGF	Drug Resistance	[116]
PLOD2	Drug Resistance	[117]
POLR1D	Drug Resistance	[118]
PGK1	Drug Resistance	[41]
NES	Drug Resistance	[119]
CERS2	Tumor suppressor	[120]
DLG1	Tumor suppressor	[121]
FLNB	Tumor suppressor	[122]

GADD45A	Tumor suppressor	[123]
HLTF	Tumor suppressor	[124]
HMG20B	Tumor suppressor	[125]
IL12RB2	Tumor suppressor	[126,127]
ILF3	Tumor suppressor	[128]
IRF6	Tumor suppressor	[129,130]
MT1G	Tumor suppressor	[131]
MT1X	Tumor suppressor	[132]
NDRG1	Tumor suppressor	[133]
RBM38	Tumor suppressor	[134]
RCL1	Tumor suppressor	[135]
RSRC1	Tumor suppressor	[136]
SEMA4B	Tumor suppressor	[137]
DDIT4	Tumor suppressor	[138,139]
EFNA1	Tumor suppressor	[140]
NRG1	Tumor suppressor	[141]
ALDH1A3	Drug Resistance (chemoresistance in colorectal cancer); Experimental target	[142,143]
HMGB1	Experimental target	[144]
CNFN	Good prognosis marker	[145]
CYSRT1	Good prognosis marker	[146,147]
IGKC	Good prognosis marker	[148]
IGKV4-1	Good prognosis marker	[149]
IGLC1	Good prognosis marker	[150]
JCHAIN	Good prognosis marker	[151]
IGHA1	Good prognosis marker	[152]
IGHG1	Good prognosis marker	[152]
IGHG2	Good prognosis marker	[152]

IGHG4	Good prognosis marker	[152]
CALB1	Oncogene	[48,153]
LCN2	Oncogene	[154]
TGFBI	Oncogene	[24]
XBP1	Oncogene	[155]
COL1A1	Poor prognosis marker	[156]
COL1A2	Poor prognosis marker	[157]
COL3A1	Poor prognosis marker	[158]
CRNN	Poor prognosis marker	[159]
ECM1	Poor prognosis marker	[160,161]
EMP1	Poor prognosis marker	[162]
ERO1A	Poor prognosis marker	[163]
GLUL	Poor prognosis marker	[164,165]
KLK13	Poor prognosis marker	[166]
KLK6	Poor prognosis marker	[167,168]
KLK7	Poor prognosis marker	[168]
KRT13	Poor prognosis marker	[169]
KRT4	Poor prognosis marker	[170]
KRT78	Poor prognosis marker	[171]
KRT8	Poor prognosis marker	[171]
LCE3D	Poor prognosis marker	[172,173]
NDRG1	Poor prognosis marker	[174,175]
PRSS27	Poor prognosis marker	[176]
SOD2	Poor prognosis marker	[91,92]
SPRR2A	Poor prognosis marker	[177,178]
SPRR2D	Poor prognosis marker	[178]
SPRR3	Poor prognosis marker	[179]

HOPX	Tumor suppressor	[180,181]
MAL	Tumor suppressor	[182]
SLURP1	Tumor suppressor	[183,184]
SLURP2	Tumor suppressor	[183]
SPINK5	Tumor suppressor	[185]
SPINK7	Tumor suppressor	[186]
TGM3	Tumor suppressor	[187,188]
TM4SF1	Tumor suppressor (gastric carcinoma); poor prognosis marker (epithelial cancers)	[189,190]

Supplementary table 4. **Identified clinical and preclinical targets.**

Gene	Drug name	Company name	Indication	Development Stage
CA9 [47,191]	SLC-0111	SignalChem Lifesciences Corp	Pancreatic Ductal Adenocarcinoma	Phase II
DUT [192]	TAS-114	Taiho Pharmaceutical Co Ltd	Adenocarcinoma Of The Gastroesophageal Junction; Gastric Cancer; Non-Small Cell Lung Cancer	Phase II
			Metastatic Breast Cancer; Metastatic Colorectal Cancer; Pancreatic Cancer	Phase I
EGFR [193]	futuximab + modotuximab	Symphogen A/S	Metastatic Colorectal Cancer	Phase III
	abivertinib maleate	Sorrento Therapeutics Inc	Metastatic Hormone Refractory (Castration Resistant, Androgen-Independent) Prostate Cancer; Burkitt Lymphoma; Diffuse Large B-Cell Lymphoma; Follicular Lymphoma; Mantle Cell Lymphoma; Marginal Zone B-cell Lymphoma; Refractory Chronic Lymphocytic Leukemia (CLL); Relapsed Chronic Lymphocytic Leukemia (CLL); Waldenstrom Macroglobulinemia (Lymphoplasmacytic Lymphoma)	Phase I
	abivertinib maleate	Sorrento Therapeutics Inc	Hairy Cell Leukemia; Non-Small Cell Lung Cancer; Prostate Cancer	Phase II

	abivertinib maleate	Sorrento Therapeutics Inc	Non-Small Cell Lung Cancer	Phase III
	afatinib dimaleate	More than 10 companies	Non-Small Cell Lung Cancer; Squamous Non-Small Cell Lung Cancer	Marketed
		Boehringer Ingelheim International GmbH	Anaplastic Astrocytoma; Anaplastic Oligoastrocytoma; Chordoma; Leptomeningeal Disease (Neoplastic Meningitis, Leptomeningeal Carcinomatosis); Low-Grade Glioma; Medulloblastoma; Meningioma; Oligodendroglioma; Pituitary Tumor; Recurrent Glioblastoma Multiforme (GBM)	Phase I
			Chordoma; Esophageal Cancer; Gastric Cancer; Metastatic Transitional (Urothelial) Tract Cancer; Squamous Cell Carcinoma; Uterine Cancer; Lymphoma; Refractory Multiple Myeloma	Phase II
	alflutinib mesylate	Allist Shanghai Pharmaceutical Technology Co Ltd	Non-Small Cell Lung Cancer	Marketed
			Lung Adenocarcinoma	Phase I
		Arrivent Biopharma Inc	Non-Small Cell Lung Cancer	Phase II
	amelimumab	Shanghai Sailun Biotechnology Co Ltd	Colorectal Cancer	Phase II
		Janssen Inc	Non-Small Cell Lung Cancer	Marketed
		Janssen-Cilag Pharma GmbH	Non-Small Cell Lung Cancer	Marketed

		Johnson & Johnson	Breast Cancer; Colorectal Cancer; Gastroesophageal (GE) Junction Carcinomas; Head and Neck Cancer Squamous Cell Carcinoma; Hepatocellular Carcinoma; Kidney Cancer (Renal Cell Cancer); Malignant Mesothelioma; Medullary Thyroid Cancer; Non-Small Cell Lung Cancer; Ovarian Cancer; Solid Tumor	Phase I
			Esophageal Cancer; Gastric Cancer; Gastroesophageal (GE) Junction Carcinomas; Metastatic Colorectal Cancer	Phase II
			Non-Small Cell Lung Cancer	Phase III
	AMX-3009	Arromax Pharmatech Co Ltd	Solid Tumor	Phase I
	ASK-120067	Jiangsu Aosaikang Pharmaceutical Co Ltd	Non-Small Cell Lung Cancer	Phase III
	aumolertinib mesylate	Jiangsu Hansoh Pharmaceutical Group Co Ltd	Non-Small Cell Lung Cancer	Marketed
		EQRx Inc	Non-Small Cell Lung Cancer	Phase III
		Jiangsu Hansoh Pharmaceutical Group Co Ltd	Non-Small Cell Lung Cancer	Phase III
	BAY-2927088	Bayer AG	Non-Small Cell Lung Cancer	Phase I

	BBT-176	Bridge Biotherapeutics Inc	Non-Small Cell Lung Cancer	Phase II
	BC-001	Dragonboat Biopharmaceutical (Shanghai) Co Ltd	Unspecified Cancer	Phase III
	BCA-101	Bicara Therapeutics Inc	Anal Cancer; Anaplastic Thyroid Cancer; Colorectal Cancer; Epithelial Ovarian Cancer; Gastric Cancer; Glioblastoma Multiforme (GBM); Head and Neck Cancer Squamous Cell Carcinoma; Hepatocellular Carcinoma; Lung Cancer; Pancreatic Cancer; Solid Tumor; Squamous Cell Carcinoma	Phase II
	BDTX-1535	Black Diamond Therapeutics Inc	Glioblastoma Multiforme (GBM); Non-Small Cell Lung Cancer	Phase I
	BEBT-109	Guangzhou BeBetter Medicine Technology Co Ltd	Non-Small Cell Lung Cancer	Phase II
	befortinib mesylate	InventisBio Co Ltd	Non-Small Cell Lung Cancer	Phase III
	BLU-451	Blueprint Medicines Corp	Non-Small Cell Lung Cancer	Phase II
	BLU-701	Zai Lab Ltd	Non-Small Cell Lung Cancer	Phase II
	BLU-945	Blueprint Medicines Corp	Non-Small Cell Lung Cancer	Phase II

	BPI-361175	Betta Pharmaceuticals Co Ltd	Non-Small Cell Lung Cancer; Solid Tumor	Phase II
	BPI-7711	Beta Pharma Inc	Non-Small Cell Lung Cancer	Phase II
	C-005	Wuxi Shuangliang Biotechnology Co Ltd	Non-Small Cell Lung Cancer	Phase I
	cetuximab	Eli Lilly Canada Inc; Merck; Bristol-Myers Squibb KK; PT. Merck Tbk;	Head And Neck Cancer Squamous Cell Carcinoma; Metastatic Colorectal Cancer	Marketed
		Merck	Metastatic Colorectal Cancer	Phase I
		TheraOp gGmbH	Metastatic Colorectal Cancer	Phase II
		Eli Lilly and Co	Anal Cancer	Phase II
		Merck	Recurrent Head and Neck Cancer Squamous Cell Carcinoma; Squamous Non-Small Cell Lung Cancer	Phase II
	cetuximab + cobimetinib + palbociclib	Cothera Bioscience Pty Ltd	Metastatic Colorectal Cancer	Phase II
		Mabpharm Ltd	Metastatic Colorectal Cancer	Phase III

	cetuximab biobetter	Dragonboat Biopharmaceutical (Shanghai) Co Ltd; Shanghai Jing Ze Biotechnology Co Ltd	Cervical Cancer; Colorectal Cancer; Endometrial Cancer; Esophageal Squamous Cell Carcinoma (ESCC); Head and Neck Cancer Squamous Cell Carcinoma; Metastatic Colorectal Cancer; Ovarian Cancer; Penile Cancer	Phase I
		Enzene Biosciences Ltd	Head And Neck Cancer Squamous Cell Carcinoma; Lip Cancer; Locally Recurrent or Locoregional Solid Malignancies; Oral Cavity (Mouth) Cancer; Pharyngeal Neoplasm	Phase III
		Ampo Biotechnology Inc; Cinnagen Co; Sichuan Kelun Pharmaceutical Co Ltd; 3SBio Inc	Metastatic Colorectal Cancer	Phase III
		R-Pharm	Recurrent Head and Neck Cancer Squamous Cell Carcinoma	Phase III
	dabrafenib mesylate + panitumumab + trametinib dimethyl sulfoxide	Novartis AG	Metastatic Colorectal Cancer	Phase II
	dacomitinib	Pfizer	Non-Small Cell Lung Cancer	Marketed
			Human Epidermal Growth Factor Receptor 2 Positive Breast Cancer (HER2+ Breast Cancer); Ovarian Cancer; Triple-Negative Breast Cancer (TNBC)	Phase I

	DBPR-112	AnBogen Therapeutics	Non-Small Cell Lung Cancer; Solid Tumor	Phase II
	DF-203	Suzhou Dingfu Target Biotechnology Co Ltd	Colorectal Cancer; Head and Neck Cancer Squamous Cell Carcinoma; Non-Small Cell Lung Cancer; Pancreatic Cancer; Renal Cell Carcinoma; Solid Tumor; Triple-Negative Breast Cancer (TNBC)	Phase I
	doxitinib mesylate	Henan Genuine Biotech Co Ltd	Non-Small Cell Lung Cancer	Phase II
	DZD-9008	Dizal (Jiangsu) Pharmaceutical Co Ltd	Solid Tumor; Non-Small Cell Lung Cancer	Phase II
	EO-1001	Senz Oncology Pty Ltd	Unspecified Cancer	Phase II
		Edison Oncology Holding Corp	Breast Cancer; Central Nervous System (CNS) Cancer; Non-Small Cell Lung Cancer	Phase II
	epertinib	Shionogi & Co Ltd	Breast Cancer; Malignant Neoplasms	Phase II
		EOC Pharma Ltd	Breast Cancer	Phase II
		Hutchison MediPharma Ltd	Glioblastoma Multiforme (GBM)	Phase II
	ERAS-801	Erasca Inc	Recurrent Glioblastoma Multiforme (GBM)	Phase I
	erlotinib	More than 15 companies	Metastatic Pancreatic Cancer; Non-Small Cell Lung Cancer	Marketed
	erlotinib hydrochloride	More than 50 companies	Metastatic Pancreatic Cancer; Non-Small Cell Lung Cancer	Marketed

	ES-072	Zhejiang Bossan Pharmaceutical Co Ltd	Non-Small Cell Lung Cancer	Phase I
	FCN-411	Fochon Pharmaceutical Ltd	Head And Neck Cancer Squamous Cell Carcinoma	Phase I
			Non-Small Cell Lung Cancer	Phase II
	FHND-9041	Jiangsu Zhengda Fenghai Pharmaceutical Co Ltd	Non-Small Cell Lung Cancer	Phase III
	Fusion Protein to Antagonize EGFR for Glioblastoma Multiforme and Malignant Glioma	Istari Oncology Inc	Glioblastoma Multiforme (GBM); Malignant Glioma	Phase II
	FWD-1509	Shenzhen Forward Pharmaceutical Co Ltd	Non-Small Cell Lung Cancer	Phase II
	GB-263	Genor BioPharma Co Ltd	Non-Small Cell Lung Cancer; Solid Tumor; Esophageal Cancer; Gastric Cancer; Head and Neck Cancer Squamous Cell Carcinoma; Metastatic Colorectal Cancer	Phase II
	GC-1118A	GC Biopharma Corp	Adenocarcinoma Of the Gastroesophageal Junction; Colon Cancer; Gastric Cancer; Metastatic Colorectal Cancer; Solid Tumor	Phase II
	gefitinib	AstraZeneca; Daiichi Sankyo Espha Co Ltd	Non-Small Cell Lung Cancer	Marketed

	H-002	RedCloud Bio Inc	Non-Small Cell Lung Cancer	Phase II
	HL-07	Hualan Biological Engineering Inc	Metastatic Colorectal Cancer	Phase II
	HS-627	Zhejiang Hisun Pharmaceutical Co Ltd	Triple-Negative Breast Cancer (TNBC)	Phase I
			Human Epidermal Growth Factor Receptor 2 Positive Breast Cancer (HER2+ Breast Cancer)	Phase III
	icotinib hydrochloride	Betta Pharmaceuticals Co Ltd	Non-Small Cell Lung Cancer	Marketed
	JMT-101	CSPC Pharmaceutical Group Ltd	Esophageal Squamous Cell Carcinoma (ESCC); Metastatic Colorectal Cancer; Non-Small Cell Lung Cancer; Solid Tumor	Phase I
			Non-Small Cell Lung Cancer	Phase II
	JS-111	Shanghai Junshi Bioscience Co Ltd	Non-Small Cell Lung Cancer	Phase II
	KBP-5209	XuanZhu Biological Technology Co Ltd	Breast Cancer; Carcinoma of Unknown Primary (Occult Primary Tumor/Cancer of Unknown Primary); Colorectal Cancer; Gallbladder Cancer; Gastric Cancer; Head and Neck Cancer; Non-Small Cell Lung Cancer; Ovarian Cancer; Pancreatic Cancer; Paranasal Sinus And Nasal Cavity Cancer; Sarcomas	Phase II
	lapatinib	Lupin Pharmaceuticals Inc; Sayre Therapeutics	Human Epidermal Growth Factor Receptor 2 Positive Breast Cancer (HER2+ Breast Cancer)	Marketed

	lapatinib ditosylate	Zhuhai Rundu Pharmaceutical Co Ltd; Hetero Healthcare Ltd; Cipla Ltd	Human Epidermal Growth Factor Receptor 2 Positive Breast Cancer (HER2+ Breast Cancer)	Marketed
	larotinib	HEC Pharma Co Ltd	Esophageal Squamous Cell Carcinoma (ESCC)	Phase III
		Yuhan Corp	Non-Small Cell Lung Cancer	Marketed
		Genosco Inc	Metastatic Brain Tumor	Phase II
		Johnson & Johnson; Genosco Inc	Non-Small Cell Lung Cancer	Phase III
	lifirafenib maleate	BeiGene Ltd	Colorectal Cancer; Endometrial Cancer; Non-Small Cell Lung Cancer; Ovarian Cancer; Pancreatic Cancer; Cholangiocarcinoma; Melanoma; Papillary Thyroid Cancer;	Phase II
	LL-191	Nalo Therapeutics Inc	Non-Small Cell Lung Cancer	Phase I
	MCLA-129	Merus NV; Betta Pharmaceuticals Co Ltd	Adenocarcinoma Of the Gastroesophageal Junction; Esophageal Squamous Cell Carcinoma (ESCC); Gastric Cancer; Head And Neck Cancer Squamous Cell Carcinoma; Non-Small Cell Lung Cancer	Phase II
	MET-306	Huadong Medicine Co Ltd	Non-Small Cell Lung Cancer	Phase III
	mobocertinib		Non-Small Cell Lung Cancer	Marketed

		Takeda Pharmaceuticals Australia Pty Ltd	Bladder Cancer; Breast Cancer; Esophageal Cancer; Gastric Cancer; Head and Neck Cancer; Metastatic Biliary Tract Cancer; Urinary Tract Cancer	Phase II
			Non-Small Cell Lung Cancer	Phase III
	MVC-101	Maverick Therapeutics Inc	Colorectal Cancer; Head and Neck Cancer Squamous Cell Carcinoma; Non-Small Cell Lung Cancer; Pancreatic Cancer; Solid Tumor	Phase II
	naquotinib mesylate	Novartis AG	Non-Small Cell Lung Cancer	Phase II
	necitumumab	Nippon Kayaku Co Ltd; Eli Lilly Canada Inc<br>Biotech Pharmaceutical Co Ltd<br>PT Kalbe Farma Tbk	Squamous Non-Small Cell Lung Cancer	Marketed
			Nasopharyngeal Cancer	Marketed
			High-Grade Glioma	Marketed
		Biocon Ltd	Head And Neck Cancer Squamous Cell Carcinoma	Marketed
		Eurofarma Laboratorios SA	Glioma; High-Grade Glioma; Pediatric Diffuse Intrinsic Pontine Glioma	Marketed
		Biotech Pharmaceutical Co Ltd	Recurrent Head and Neck Cancer Squamous Cell Carcinoma	Phase I
		Biotech Pharmaceutical Co Ltd	Head And Neck Cancer Squamous Cell Carcinoma	Phase II

		InnoMab Pte Ltd	Cervical Cancer	Phase III
		Biotech Pharmaceutical Co Ltd	Pediatric Diffuse Intrinsic Pontine Glioma	Phase III
	NRC-2694	Natco Pharma Ltd	Hypopharyngeal Cancer; Laryngeal Cancer; Oral Cavity (Mouth) Cancer; Oropharyngeal Cancer; Recurrent Head and Neck Cancer Squamous Cell Carcinoma	Phase II
	olafertinib	Checkpoint Therapeutics Inc; Suzhou Neupharma Co Ltd	Non-Small Cell Lung Cancer	Phase II
	olmutinib hydrochloride	Hanmi Pharmaceuticals Co Ltd	Non-Small Cell Lung Cancer	Marketed
	osimertinib mesylate	AstraZeneca	Non-Small Cell Lung Cancer	Marketed
	panitumumab	Amgen; PT Glaxo Wellcome; Takeda Pharmaceutical Co Ltd; Amgen Inc; Dr. Reddy's Laboratories Ltd	Metastatic Colorectal Cancer	Marketed

	PB-357	Puma Biotechnology Inc	Breast Cancer	Phase I
	petosemtamab	Merus NV	Metastatic Colorectal Cancer; Solid Tumor	Phase II
	poziotinib hydrochloride	Hanmi Pharmaceuticals Co Ltd	Esophageal Cancer	Phase I
			Human Epidermal Growth Factor Receptor 2 Positive Breast Cancer (HER2+ Breast Cancer); Lung Adenocarcinoma	Phase II
			Non-Small Cell Lung Cancer	Phase III
	pyrotinib	Jiangsu Hengrui Medicine Co Ltd	Human Epidermal Growth Factor Receptor 2 Positive Breast Cancer (HER2+ Breast Cancer)	Marketed
	QL-1105	Qilu Pharmaceutical Co Ltd	Colorectal Cancer; Head and Neck Cancer	Phase I
	QL-1203	Qilu Pharmaceutical Co Ltd	Metastatic Colorectal Cancer	Phase III
	RXDX-105	F. Hoffmann-La Roche Ltd	Leptomeningeal Disease (Neoplastic Meningitis, Leptomeningeal Carcinomatosis); Lung Adenocarcinoma; Medullary Thyroid Cancer; Ovarian Cancer; Solid Tumor; Squamous Non-Small Cell Lung Cancer	Phase I
	sapitinib	AstraZeneca Plc	Metastatic Colorectal Cancer	Phase III
	SCT-200	SinoCelltech Group Ltd	Colorectal Cancer	Phase I
			Recurrent Head and Neck Cancer Squamous Cell Carcinoma	Phase II

			Esophageal Squamous Cell Carcinoma (ESCC); Metastatic Colorectal Cancer; Triple-Negative Breast Cancer (TNBC)	Phase II
	selatinib ditosilate	Qilu Pharmaceutical Co Ltd	Breast Cancer	Phase I
	sirotinib	XuanZhu Biological Technology Co Ltd	Esophageal Squamous Cell Carcinoma (ESCC); Gastric Cancer; Lung Cancer	Phase I
	SKLB-1028	CSPC Pharmaceutical Group Ltd	Acute Myelocytic Leukemia (AML, Acute Myeloblastic Leukemia)	Phase II
			Refractory Acute Myeloid Leukemia; Relapsed Acute Myeloid Leukemia	Phase III
	SPH-118811	Shanghai Pharmaceutical Group Co Ltd	Non-Small Cell Lung Cancer	Phase I
	Sutetinib maleate	Suzhou Teligene Ltd	Non-Small Cell Lung Cancer	Phase II
	SYHA-12128	CSPC Pharmaceutical Group Ltd	Adenocarcinoma Of the Gastroesophageal Junction; Colorectal Cancer; Gastric Cancer	Phase I
			Bile Duct Cancer (Cholangiocarcinoma); Extrahepatic Bile Duct Cancer; Gallbladder Cancer; Metastatic Biliary Tract Cancer; Medullary Thyroid Cancer; Non-Small Cell Lung Cancer	Phase II
	SYN-004	Synermore Biologics Co Ltd	Head And Neck Cancer Squamous Cell Carcinoma; Metastatic Colorectal Cancer; Non-Small Cell Lung Cancer; Solid Tumor	Phase I

	TAS-2940	Taiho Oncology Inc	Glioblastoma Multiforme (GBM); Human Epidermal Growth Factor Receptor 2 Positive Breast Cancer (HER2+ Breast Cancer); Non-Small Cell Lung Cancer	Phase I
	tesevatinib tosylate	Kadmon Holdings Inc	Leptomeningeal Disease (Neoplastic Meningitis, Leptomeningeal Carcinomatosis); Metastatic Brain Tumor; Recurrent Glioblastoma Multiforme (GBM); Non-Small Cell Lung Cancer	Phase II
	tomuzotuximab	Glycotope GmbH	Breast Cancer; Esophageal Cancer; Gastric Cancer; Gynecological Cancer; Kidney Cancer (Renal Cell Cancer); Metastatic Colorectal Cancer; Non-Small Cell Lung Cancer	Phase I
			Recurrent Head and Neck Cancer Squamous Cell Carcinoma	Phase II
	TQB-3804	Chia Tai Tianqing Pharmaceutical Group Co Ltd	Malignant Neoplasms	Phase I
	Vaccine	Center of Molecular Immunology	Hormone Refractory (Castration Resistant, Androgen-Independent) Prostate Cancer	Phase I
	vandetanib	Sanofi-Aventis (Suisse); Medley Industria Farmaceutica Ltda; Genzyme Europe BV	Medullary Thyroid Cancer	Marketed
	varlitinib	Aslan Pharmaceuticals Ltd	Bladder Cancer; Breast Cancer; Colorectal Cancer; Gastrointestinal Tumor; Glioblastoma Multiforme (GBM); Head and Neck Cancer Squamous Cell Carcinoma; Hepatobiliary System Tumor; Metastatic Hepatocellular	Phase I

			Carcinoma (HCC); Non-Small Cell Lung Cancer; Ovarian Cancer; Pancreatic Cancer; Prostate Cancer	
			Gastric Cancer; Human Epidermal Growth Factor Receptor 2 Positive Breast Cancer (HER2+ Breast Cancer); Gallbladder Cancer	Phase II
			Gastroesophageal (GE) Junction Carcinomas; Cholangiocarcinoma	Phase III
			Papillary Thyroid Cancer; Solid Tumor	Phase III
	VRN-07	Voronoi Group	Solid Tumor	Phase I
	WJ-13404	Wigen Biomedicine Technology (Shanghai) Co Ltd	Non-Small Cell Lung Cancer	Phase II
	WSD-0922	Wayshine Biopharma Inc	Non-Small Cell Lung Cancer; Anaplastic Astrocytoma; Glioblastoma Multiforme (GBM)	Phase I
	XZP-5809	Sihuan Pharmaceutical Holdings Group Ltd	Non-Small Cell Lung Cancer	Phase I
	yinlitinib	HEC Pharma Co Ltd	Breast Cancer	Phase I
	YZJ-0318	Yangtze River Pharmaceutical Group	Non-Small Cell Lung Cancer	Phase I
	zipalertinib	Taiho Pharmaceutical Co Ltd; Zai Lab Ltd	Non-Small Cell Lung Cancer	Phase II

	ZNE-4	Zentalis Pharmaceuticals Inc	Non-Small Cell Lung Cancer	Phase II
	zorifertinib	Alpha Biopharma Ltd	Non-Small Cell Lung Cancer	Phase III
PGF [116]	conbercept	Chengdu Kanghong Pharmaceuticals Group Co Ltd	Retinoblastoma	Phase II
	ziv-aflibercept	Sanofi-Aventis	Metastatic Colorectal Cancer	Marketed
	ziv-aflibercept	Regeneron Pharmaceuticals Inc	Uveal Melanoma	Phase II
	THR-317	Oncurious NV	Medulloblastoma	Phase II
	ziv-aflibercept biosimilar	Luye Pharma Group Ltd	Metastatic Colorectal Cancer	Phase II
CXCL8 [194]	BMS-986253	Bristol-Myers Squibb Co	Head And Neck Cancer Squamous Cell Carcinoma; Hepatocellular Carcinoma; Hormone-Sensitive Prostate Cancer; Melanoma; Non-Small Cell Lung Cancer; Solid Tumor	Phase II
			Melanoma; Renal Cell Carcinoma	Phase I
ITGA2 [195]	E-7820	Eisai Co Ltd	Chronic Myelomonocytic Leukemia (CMML); Myelodysplastic Syndrome; Refractory Acute Myeloid Leukemia; Relapsed Acute Myeloid Leukemia	Phase II
HK2 [196]	tuvatexib	Vidac Pharma	Cutaneous T-Cell Lymphoma	Phase II

HMGB 1 [197]	dociparstat sodium	Chimerix Inc	Acute Myelocytic Leukemia (AML, Acute Myeloblastic Leukemia)	Phase III
CLDN1 [198]	ALEF-02	Alentis Therapeutics AG	Solid Tumor	Phase I
VEGFA [199]	ABL-001	Compass Therapeutics Inc	Extrahepatic Bile Duct Cancer; Gallbladder Cancer; Metastatic Biliary Tract Cancer; Metastatic Colorectal Cancer	Phase I
		Handok Inc; Elpiscience Biopharma Ltd	Bile Duct Cancer (Cholangiocarcinoma); Extrahepatic Bile Duct Cancer; Gallbladder Cancer; Gastric Cancer; Gastrointestinal Stromal Tumor (GIST); Metastatic Colorectal Cancer; Non-Small Cell Lung Cancer; Ovarian Cancer; Pancreatic Cancer; Solid Tumor	Phase II
	bevacizumab	Roche; Chugai Pharmaceutical Co Ltd; Genentech; Cipla; PT Boehringer Ingelheim	Cervical Cancer; Epithelial Ovarian Cancer; Fallopian Tube Cancer; Metastatic Breast Cancer; Metastatic Colorectal Cancer; Metastatic Renal Cell Carcinoma; Non-Small Cell Lung Cancer; Peritoneal Cancer	Marketed
	bevacizumab	Chugai Pharmaceutical Co Ltd; F. Hoffmann-La Roche Ltd	Hepatocellular Carcinoma; Human Epidermal Growth Factor Receptor 2 Negative Breast Cancer (HER2- Breast Cancer)	Phase II
	bevacizumab	Chugai Pharmaceutical Co Ltd; F. Hoffmann-La Roche Ltd	Cervical Cancer; Malignant Pleural Mesothelioma; Melanoma; Hepatocellular Carcinoma; Metastatic Colorectal Cancer; Lung Cancer; Human Epidermal Growth Factor Receptor 2 Negative Breast Cancer (HER2- Breast Cancer)	Phase III

	bevacizumab + paclitaxel	Sorrento Therapeutics Inc	Adenocarcinoma; Cervical Cancer; Clear Cell Squamous Cell Carcinoma; Endometrial Cancer; Fallopian Tube Cancer; Melanoma; Ovarian Cancer; Peritoneal Cancer; Squamous Cell Carcinoma	Phase I
	bevacizumab biosimilar	Pfizer; Amgen Inc; Daiichi Sankyo Co Ltd; PT Pyridam Farma Tbk; BeiGene Ltd; Cipla; Celltrion Inc; Eris Lifesciences Ltd; Biocad; Qilu Pharmaceutical Co Ltd; Merck	Epithelial Ovarian Cancer; Fallopian Tube Cancer; Glioblastoma Multiforme (GBM); Metastatic Colorectal Cancer; Non-Small Cell Lung Cancer; Peritoneal Cancer; Cervical Cancer; Fallopian Tube Cancer; Metastatic Breast Cancer; Metastatic Renal Cell Carcinoma; Peritoneal Cancer	Marketed
	ziv-aflibercept	Sanofi-Aventis	Metastatic Colorectal Cancer	Marketed
		Regeneron Pharmaceuticals Inc	Uveal Melanoma	Phase II
	ziv-aflibercept biosimilar	Luye Pharma Group Ltd	Metastatic Colorectal Cancer	Phase II
TFRC	CX-2029	CytomX Therapeutics Inc	Diffuse Large B-Cell Lymphoma; Squamous Non-Small Cell Lung Cancer.<br>Adenoid Cystic Carcinoma (ACC); Bladder Cancer; Colorectal Cancer; Esophageal Cancer; Gastroesophageal (GE) Junction Carcinomas; Head and Neck Cancer Squamous Cell Carcinoma; Hepatocellular Carcinoma; Kidney Cancer (Renal Cell Cancer); Malignant Pleural Mesothelioma; Ocular Melanoma; Ovarian Cancer; Pancreatic Cancer; Prostate Cancer; Soft Tissue	Phase II

			Sarcoma; Solid Tumor; Thymic Carcinoma; Thymoma (Thymic Epithelial Tumor); Thyroid Cancer	
	INA-03	Inatherys	Acute Lymphocytic Leukemia (ALL, Acute Lymphoblastic Leukemia); Acute Myelocytic Leukemia (AML, Acute Myeloblastic Leukemia); Leukemia; Refractory Acute Myeloid Leukemia; Relapsed Acute Myeloid Leukemia	Phase I
	JSTTFR-09	Fujifilm Holdings Corp	Polycythemia Vera	Phase I
PFKFB3 [200]	KAN-0438757	Kancera AB	Triple-Negative Breast Cancer (TNBC)	Preclinical
CIB1 [201-203]	Small Molecules	Reveris Therapeutics LLC	Breast Cancer	Preclinical
HSP90AA1 [204]	Small Molecule	China Medical University	Unspecified Cancer	Preclinical

Supplementary References

1. Shen A, Liu L, Huang Y, et al. Targeting Spindle-Associated Protein HAUS6 Prevents Cell Growth of Colorectal Cancer Cells by Activating the P53/P21 Pathway. 2020.
2. Hernandez P, Sole X, Valls J, et al. Integrative analysis of a cancer somatic mutome. Mol Cancer 2007;6:13.
3. Dart AE, Worth DC, Muir G, et al. The drebrin/EB3 pathway drives invasive activity in prostate cancer. Oncogene 2017;36:4111-23.
4. Yu Q, Tang F, Cao F, Tan X, Weng L, Sun L. Drebrin promotes lung adenocarcinoma cell migration through inducing integrin beta1 endocytosis. Biochem Biophys Res Commun 2022;630:175-82.
5. Muller D, Millon R, Theobald S, et al. Cyclin L1 (CCNL1) gene alterations in human head and neck squamous cell carcinoma. Br J Cancer 2006;94:1041-4.
6. Chuu C-P, Lin C-Y, Huang S-H, Tsai KK-C. ACTL6A is a novel oncogene and prognostic biomarker for prostate cancer. Annals of Oncology 2019;30:vi106.
7. Sykaras AG, Pergaris A, Theocharis S. Challenging, Accurate and Feasible: CAF-1 as a Tumor Proliferation Marker of Diagnostic and Prognostic Value. Cancers (Basel) 2021;13.
8. Camilo V, Henrique R. Oncogenic potential of CHAF1A in gastric cancer: A novel link with Helicobacter pylori-driven carcinogenesis? EBioMedicine 2018;38:3-4.
9. Ishida K, Nakashima T, Shibata T, Hara A, Tomita H. Role of the DEK oncogene in the development of squamous cell carcinoma. Int J Clin Oncol 2020;25:1563-9.
10. Fields AP, Justilien V. The guanine nucleotide exchange factor (GEF) Ect2 is an oncogene in human cancer. Adv Enzyme Regul 2010;50:190-200.
11. Qin A, Liu Q, Wang J. Ropivacaine inhibits proliferation, invasion, migration and promotes apoptosis of papillary thyroid cancer cells via regulating ITGA2 expression. Drug Dev Res 2020;81:700-7.
12. Rutkovsky AC, Yeh ES, Guest ST, et al. Eukaryotic initiation factor 4E-binding protein as an oncogene in breast cancer. BMC Cancer 2019;19:491.
13. Cao S, Zheng J, Liu X, et al. FXR1 promotes the malignant biological behavior of glioma cells via stabilizing MIR17HG. J Exp Clin Cancer Res 2019;38:37.
14. Lukey MJ, Greene KS, Erickson JW, Wilson KF, Cerione RA. The oncogenic transcription factor c-Jun regulates glutaminase expression and sensitizes cells to glutaminase-targeted therapy. Nat Commun 2016;7:11321.
15. Lee DH, Kim GW, Yoo J, et al. Histone demethylase KDM4C controls tumorigenesis of glioblastoma by epigenetically regulating p53 and c-Myc. Cell Death Dis 2021;12:89.
16. Qin H, Li Y, Zhang H, et al. Prognostic implications and oncogenic roles of MYBL2 protein expression in esophageal squamous-cell carcinoma. Onco Targets Ther 2019;12:1917-27.
17. Ahmadi SE, Rahimi S, Zarandi B, Chegeni R, Safa M. MYC: a multipurpose oncogene with prognostic and therapeutic implications in blood malignancies. J Hematol Oncol 2021;14:121.
18. Wang JJ, Zou JX, Wang H, et al. Histone methyltransferase NSD2 mediates the survival and invasion of triple-negative breast cancer cells via stimulating ADAM9-EGFR-AKT signaling. Acta Pharmacol Sin 2019;40:1067-75.
19. Liang J, Xia L, Oyang L, et al. The functions and mechanisms of prefoldin complex and prefoldin-subunits. Cell Biosci 2020;10:87.
20. Singh DK, Gholamalamdari O, Jadaliha M, et al. PSIP1/p75 promotes tumorigenicity in breast cancer cells by promoting the transcription of cell cycle genes. Carcinogenesis 2017;38:966-75.

21. Zhang D, Jiang Q, Ge X, et al. RHOV promotes lung adenocarcinoma cell growth and metastasis through JNK/c-Jun pathway. Int J Biol Sci 2021;17:2622-32.
22. Zhao Z, Lu L, Li W. TAGLN2 promotes the proliferation, invasion, migration and epithelial-mesenchymal transition of colorectal cancer cells by activating STAT3 signaling through ANXA2. Oncol Lett 2021;22:737.
23. Meng T, Liu L, Hao R, Chen S, Dong Y. Transgelin-2: A potential oncogenic factor. Tumor Biol 2017;39:1010428317702650.
24. Chen Y, Zhao H, Feng Y, et al. Pan-Cancer Analysis of the Associations of TGFBI Expression With Prognosis and Immune Characteristics. Front Mol Biosci 2021;8:745649.
25. Li L, Duan Q, Zeng Z, et al. UHRF2 promotes intestinal tumorigenesis through stabilization of TCF4 mediated Wnt/beta-catenin signaling. Int J Cancer 2020;147:2239-52.
26. Chen X, Chen SI, Liu XA, Zhou WB, Ma RR, Chen L. Vav3 oncogene is upregulated and a poor prognostic factor in breast cancer patients. Oncol Lett 2015;9:2143-8.
27. Aguilar H, Urruticoechea A, Halonen P, et al. VAV3 mediates resistance to breast cancer endocrine therapy. Breast Cancer Res 2014;16:R53.
28. Li D, Masiero M, Banham AH, Harris AL. The notch ligand JAGGED1 as a target for anti-tumor therapy. Front Oncol 2014;4:254.
29. Xiu MX, Liu YM, Kuang BH. The oncogenic role of Jagged1/Notch signaling in cancer. Biomed Pharmacother 2020;129:110416.
30. Wang L, Wang H, Yang C, et al. Investigating CENPW as a Novel Biomarker Correlated With the Development and Poor Prognosis of Breast Carcinoma. Front Genet 2022;13:900111.
31. Zhou Y, Chai H, Guo L, et al. Knockdown of CENPW Inhibits Hepatocellular Carcinoma Progression by Inactivating E2F Signaling. Technol Cancer Res Treat 2021;20:15330338211007253.
32. Ma RM, Yang F, Huang DP, Zheng M, Wang YL. The Prognostic Value of the Expression of SMC4 mRNA in Breast Cancer. Dis Markers 2019;2019:2183057.
33. Jiang L, Zhou J, Zhong D, et al. Overexpression of SMC4 activates TGFbeta/Smad signaling and promotes aggressive phenotype in glioma cells. Oncogenesis 2017;6:e301.
34. Martins VL, Vyas JJ, Chen M, et al. Increased invasive behaviour in cutaneous squamous cell carcinoma with loss of basement-membrane type VII collagen. J Cell Sci 2009;122:1788-99.
35. Nilchian A, Johansson J, Ghalali A, et al. CXADR-Mediated Formation of an AKT Inhibitory Signalosome at Tight Junctions Controls Epithelial-Mesenchymal Plasticity in Breast Cancer. Cancer Res 2019;79:47-60.
36. Nagy A, Pongor LS, Szabo A, Santarpia M, Gyorffy B. KRAS driven expression signature has prognostic power superior to mutation status in non-small cell lung cancer. Int J Cancer 2017;140:930-7.
37. Hayashi M, Okabe K, Yamawaki Y, et al. Loss of lysophosphatidic acid receptor-3 enhances cell migration in rat lung tumor cells. Biochem Biophys Res Commun 2011;405:450-4.
38. Liu X, Liu P, Chernock RD, et al. A prognostic gene expression signature for oropharyngeal squamous cell carcinoma. EBioMedicine 2020;61:102805.
39. Li D, Zhao W, Zhang X, Lv H, Li C, Sun L. NEFM DNA methylation correlates with immune infiltration and survival in breast cancer. Clin Epigenetics 2021;13:112.
40. Kim BK, Yoo HI, Lee AR, Choi K, Yoon SK. Decreased expression of VLDLR is inversely correlated with miR-200c in human colorectal cancer. Mol Carcinog 2017;56:1620-9.

41. He Y, Luo Y, Zhang D, et al. PGK1-mediated cancer progression and drug resistance. Am J Cancer Res 2019;9:2280-302.
42. Zhao J, Liu J, Wu N, et al. ANGPTL4 overexpression is associated with progression and poor prognosis in breast cancer. Oncol Lett 2020;20:2499-505.
43. Zhang J, Lan Z, Qiu G, et al. Over-expression of ANP32E is associated with poor prognosis of pancreatic cancer and promotes cell proliferation and migration through regulating beta-catenin. BMC Cancer 2020;20:1065.
44. Lek SM, Li K, Tan QX, et al. Pairing a prognostic target with potential therapeutic strategy for head and neck cancer. Oral Oncol 2020;111:105035.
45. Kiss Z, Mudryj M, Ghosh PM. Non-circadian aspects of BHLHE40 cellular function in cancer. Genes Cancer 2020;11:1-19.
46. Sethuraman A, Brown M, Krutilina R, et al. BHLHE40 confers a pro-survival and pro-metastatic phenotype to breast cancer cells by modulating HBEGF secretion. Breast Cancer Res 2018;20:117.
47. Mboge MY, McKenna R, Frost SC. Advances in Anti-Cancer Drug Development Targeting Carbonic Anhydrase IX and XII. Top Anticancer Res 2015;5:3-42.
48. Cao LQ, Wang YN, Liang M, Pan MZ. CALB1 enhances the interaction between p53 and MDM2, and inhibits the senescence of ovarian cancer cells. Mol Med Rep 2019;19:5097-104.
49. Wang Y, Liu P, Zhang Z, Wang J, Cheng Z, Fan C. Identification of CCT3 as a prognostic factor and correlates with cell survival and invasion of head and neck squamous cell carcinoma. Biosci Rep 2021;41.
50. Xu G, Bu S, Wang X, Zhang H, Ge H. Suppression of CCT3 inhibits the proliferation and migration in breast cancer cells. Cancer Cell Int 2020;20:218.
51. Dou L, Zhang X. Upregulation of CCT3 promotes cervical cancer progression through FN1. Mol Med Rep 2021;24.
52. de Freitas GPA, Geraldo LHM, Faria BM, et al. Centromere protein J is overexpressed in human glioblastoma and promotes cell proliferation and migration. J Neurochem 2022;162:501-13.
53. Sun L, Wang Y, Yuan H, et al. CPA4 is a Novel Diagnostic and Prognostic Marker for Human Non-Small-Cell Lung Cancer. J Cancer 2016;7:1197-204.
54. Zhang X, Zhang X, Mao T, et al. CSE1L, as a novel prognostic marker, promotes pancreatic cancer proliferation by regulating the AKT/mTOR signaling pathway. J Cancer 2021;12:2797-806.
55. Yang X, Wang J, Li WP, Jin ZJ, Liu XJ. Desmocollin 3 mediates follicle stimulating hormone-induced ovarian epithelial cancer cell proliferation by activating the EGFR/Akt signaling pathway. Int J Clin Exp Pathol 2015;8:6716-23.
56. Jung YS, Park JI. Wnt signaling in cancer: therapeutic targeting of Wnt signaling beyond beta-catenin and the destruction complex. Exp Mol Med 2020;52:183-91.
57. Lyu S, Lu J, Chen W, et al. High expression of eIF4A2 is associated with a poor prognosis in esophageal squamous cell carcinoma. Oncol Lett 2020;20:177.
58. Liu L, Cai L, Liu C, et al. Construction and Validation of a Novel Glycometabolism-Related Gene Signature Predicting Survival in Patients With Ovarian Cancer. Front Genet 2020;11:585259.
59. Li L, Fu LQ, Wang HJ, Yan ZL, Yu XC, Wang YY. FAT2 is a novel independent prognostic factor for the poor prognosis of gastric carcinoma. Int J Clin Exp Pathol 2017;10:11603-9.

60. Anuraga G, Tang WC, Phan NN, et al. Comprehensive Analysis of Prognostic and Genetic Signatures for General Transcription Factor III (GTF3) in Clinical Colorectal Cancer Patients Using Bioinformatics Approaches. Curr Issues Mol Biol 2021;43.
61. Liu C, Zhou X, Zeng H, Wu D, Liu L. HILPDA Is a Prognostic Biomarker and Correlates With Macrophage Infiltration in Pan-Cancer. Front Oncol 2021;11:597860.
62. Guan Y, Zhu X, Liang J, Wei M, Huang S, Pan X. Upregulation of HSPA1A/HSPA1B/HSPA7 and Downregulation of HSPA9 Were Related to Poor Survival in Colon Cancer. Front Oncol 2021;11:749673.
63. Chu S, Wen Q, Qing Z, et al. High expression of heat shock protein 10 correlates negatively with estrogen/progesterone receptor status and predicts poor prognosis in invasive ductal breast carcinoma. Hum Pathol 2017;61:173-80.
64. Fan G, Tu Y, Wu N, Xiao H. The expression profiles and prognostic values of HSPs family members in Head and neck cancer. Cancer Cell Int 2020;20:220.
65. Xiao F, Dai Y, Hu Y, Lu M, Dai Q. Expression profile analysis identifies IER3 to predict overall survival and promote lymph node metastasis in tongue cancer. Cancer Cell Int 2019;19:307.
66. Fang J, Wang H, Liu Y, Ding F, Ni Y, Shao S. High KRT8 expression promotes tumor progression and metastasis of gastric cancer. Cancer Sci 2017;108:178-86.
67. Gordon-Weeks A, Lim SY, Yuzhalin A, et al. Tumor-Derived Laminin alpha5 (LAMA5) Promotes Colorectal Liver Metastasis Growth, Branching Angiogenesis and Notch Pathway Inhibition. Cancers (Basel) 2019;11.
68. Galatenko VV, Maltseva DV, Galatenko AV, Rodin S, Tonevitsky AG. Cumulative prognostic power of laminin genes in colorectal cancer. BMC Med Genomics 2018;11:9.
69. Zhao T, Zhang Y, Ma X, et al. Elevated expression of LPCAT1 predicts a poor prognosis and is correlated with the tumor microenvironment in endometrial cancer. Cancer Cell Int 2021;21:269.
70. Tao M, Luo J, Gu T, et al. LPCAT1 reprogramming cholesterol metabolism promotes the progression of esophageal squamous cell carcinoma. Cell Death Dis 2021;12:845.
71. Yang W, Li J, Zhang M, et al. Elevated expression of the rhythm gene NFIL3 promotes the progression of TNBC by activating NF-kappaB signaling through suppression of NFKBIA transcription. J Exp Clin Cancer Res 2022;41:67.
72. Poodineh J, Sirati-Sabet M, Rajabibazl M, Ghasemian M, Mohammadi-Yeganeh S. Downregulation of NRARP exerts anti-tumor activities in the breast tumor cells depending on Wnt/beta-catenin-mediated signals: The role of miR-130a-3p. Chem Biol Drug Des 2022;100:334-45.
73. Zhang M, Qin Y, Zuo B, et al. Overexpression of NOTCH-regulated Ankyrin Repeat Protein is associated with papillary thyroid carcinoma progression. PLoS One 2017;12:e0167782.
74. Shi J, Li C, Wang H, Xiao B, Qiu W. NUP58 facilitates metastasis and epithelial-mesenchymal transition of lung adenocarcinoma via the GSK-3beta/Snail signaling pathway. Am J Transl Res 2019;11:393-405.
75. Yang Z, Wei B, Qiao A, et al. A novel EZH2/NXPH4/CDKN2A axis is involved in regulating the proliferation and migration of non-small cell lung cancer cells. Biosci Biotechnol Biochem 2022;86:340-50.
76. Tantiwetrueangdet A, Panvichian R, Sornmayura P, Leelaudomlipi S, Macoska JA. PCNA-associated factor (KIAA0101/PCLAF) overexpression and gene copy number alterations in hepatocellular carcinoma tissues. BMC Cancer 2021;21:295.
77. Shen J, Jin Z, Lv H, et al. PFKP is highly expressed in lung cancer and regulates glucose metabolism. Cell Oncol (Dordr) 2020;43:617-29.

78. Liu M, Chen S, Zhang A, Zheng Q, Fu J. PLAUR as a Potential Biomarker Associated with Immune Infiltration in Bladder Urothelial Carcinoma. J Inflamm Res 2021;14:4629-41.
79. Wang Z, Qi H, Zhang Y, Sun H, Dong J, Wang H. PLPP2: Potential therapeutic target of breast cancer in PLPP family. Immunobiology 2022;227:152298.
80. Xu Y, Wang J, Cai S, et al. PNCK depletion inhibits proliferation and induces apoptosis of human nasopharyngeal carcinoma cells in vitro and in vivo. J Cancer 2019;10:6925-32.
81. Lin YY, Wang CY, Phan NN, et al. PODXL2 maintains cellular stemness and promotes breast cancer development through the Rac1/Akt pathway. Int J Med Sci 2020;17:1639-51.
82. Zhao H, Lu G. Prognostic Implication and Immunological Role of PSMD2 in Lung Adenocarcinoma. Front Genet 2022;13:905581.
83. Chang WM, Lin YF, Su CY, et al. Parathyroid Hormone-Like Hormone is a Poor Prognosis Marker of Head and Neck Cancer and Promotes Cell Growth via RUNX2 Regulation. Sci Rep 2017;7:41131.
84. Weekes D, Noel E, Walker C, et al. PUM3 is a triple-negative breast cancer dependency gene that functions in replication fork restart and repair. Cancer Research 2018;78:3363-.
85. He ZY, Wu SG, Peng F, et al. Up-Regulation of RFC3 Promotes Triple Negative Breast Cancer Metastasis and is Associated With Poor Prognosis Via EMT. Transl Oncol 2017;10:1-9.
86. Zhang J, Wang L, Xie X. RFC4 promotes the progression and growth of Oral Tongue squamous cell carcinoma in vivo and vitro. J Clin Lab Anal 2021;35:e23761.
87. Li H, Zhou T, Zhang Y, Jiang H, Zhang J, Hua Z. RuvBL1 Maintains Resistance to TRAIL-Induced Apoptosis by Suppressing c-Jun/AP-1 Activity in Non-Small Cell Lung Cancer. Front Oncol 2021;11:679243.
88. Mou Y, Zhang L, Liu Z, Song X. Abundant expression of ferroptosis-related SAT1 is related to unfavorable outcome and immune cell infiltration in low-grade glioma. BMC Cancer 2022;22:215.
89. Min KW, Kim DH, Son BK, et al. High SLC2A1 expression associated with suppressing CD8 T cells and B cells promoted cancer survival in gastric cancer. PLoS One 2021;16:e0245075.
90. Alves CL, Elias D, Lyng MB, Bak M, Ditzel HJ. SNAI2 upregulation is associated with an aggressive phenotype in fulvestrant-resistant breast cancer cells and is an indicator of poor response to endocrine therapy in estrogen receptor-positive metastatic breast cancer. Breast Cancer Res 2018;20:60.
91. Jung CH, Kim EM, Song JY, Park JK, Um HD. Mitochondrial superoxide dismutase 2 mediates gamma-irradiation-induced cancer cell invasion. Exp Mol Med 2019;51:1-10.
92. Amano T, Chano T, Isono T, Kimura F, Kushima R, Murakami T. Abundance of mitochondrial superoxide dismutase is a negative predictive biomarker for endometriosis-associated ovarian cancers. World J Surg Oncol 2019;17:24.
93. Sun R, Jiang B, Qi H, et al. SOX4 contributes to the progression of cervical cancer and the resistance to the chemotherapeutic drug through ABCG2. Cell Death Dis 2015;6:e1990.
94. Lin C, Sun L, Huang S, Weng X, Wu Z. STC2 Is a Potential Prognostic Biomarker for Pancreatic Cancer and Promotes Migration and Invasion by Inducing Epithelial-Mesenchymal Transition. Biomed Res Int 2019;2019:8042489.
95. Zhang C, Chen S, Ma X, et al. Upregulation of STC2 in colorectal cancer and its clinicopathological significance. Onco Targets Ther 2019;12:1249-58.
96. Wang X, Xu X, Cai W, et al. TBL1XR1 mutation predicts poor outcome in primary testicular diffuse large B-cell lymphoma patients. Biomark Res 2020;8:10.

97. Lee YY, Wei YC, Tian YF, et al. Overexpression of Transcobalamin 1 is an Independent Negative Prognosticator in Rectal Cancers Receiving Concurrent Chemoradiotherapy. J Cancer 2017;8:1330-7.
98. Tang N, Cai X, Peng L, Liu H, Chen Y. TCP1 regulates Wnt7b/beta-catenin pathway through P53 to influence the proliferation and migration of hepatocellular carcinoma cells. Signal Transduct Target Ther 2020;5:169.
99. Yasui K, Okamoto H, Arii S, Inazawa J. Association of over-expressed TFDP1 with progression of hepatocellular carcinomas. J Hum Genet 2003;48:609-13.
100. Liu J, Sun M, Sun Y, Li H. TMEM189 promotes breast cancer through inhibition of autophagy-regulated ferroptosis. Biochem Biophys Res Commun 2022;622:37-44.
101. Yu J, Qu L, Xia Y, et al. TMEM189 negatively regulates the stability of ULK1 protein and cell autophagy. Cell Death Dis 2022;13:316.
102. An J, Luo Z, An W, Cao D, Ma J, Liu Z. Identification of spliceosome components pivotal to breast cancer survival. RNA Biol 2021;18:833-42.
103. Zeng Z, Lei S, He Z, Chen T, Jiang J. YEATS2 is a target of HIF1alpha and promotes pancreatic cancer cell proliferation and migration. J Cell Physiol 2021;236:2087-98.
104. Chen J, Wang Z, Gao S, et al. Human drug efflux transporter ABCC5 confers acquired resistance to pemetrexed in breast cancer. Cancer Cell Int 2021;21:136.
105. Hong X, Michalski CW, Kong B, et al. ALCAM is associated with chemoresistance and tumor cell adhesion in pancreatic cancer. J Surg Oncol 2010;101:564-9.
106. Larrue C, Guiraud N, Mouchel PL, et al. Adrenomedullin-CALCRL axis controls relapse-initiating drug tolerant acute myeloid leukemia cells. Nat Commun 2021;12:422.
107. Huang Z, Zhang H, Xing C, et al. Identification and validation of CALCRL-associated prognostic genes in acute myeloid leukemia. Gene 2022;809:146009.
108. Nagler A, Vredevoogd DW, Alon M, et al. A genome-wide CRISPR screen identifies FBXO42 involvement in resistance toward MEK inhibition in NRAS-mutant melanoma. Pigment Cell Melanoma Res 2020;33:334-44.
109. Zhao L, Pan Y, Gang Y, et al. Identification of GAS1 as an epirubicin resistance-related gene in human gastric cancer cells with a partially randomized small interfering RNA library. J Biol Chem 2009;284:26273-85.
110. Kudo-Saito C, Miyamoto T, Imazeki H, Shoji H, Aoki K, Boku N. IL33 Is a Key Driver of Treatment Resistance of Cancer. Cancer Res 2020;80:1981-90.
111. Wang J, Da C, Su Y, Song R, Bai Z. MKNK2 enhances chemoresistance of ovarian cancer by suppressing autophagy via miR-125b. Biochem Biophys Res Commun 2021;556:31-8.
112. Zhao Z, Zhang G, Li W. MT2A Promotes Oxaliplatin Resistance in Colorectal Cancer Cells. Cell Biochem Biophys 2020;78:475-82.
113. Li WM, Ke HL, Kuo YH, et al. High MT2A Expression Predicts Worse Prognosis in Patients with Urothelial Carcinoma. Oncology 2022;100:485-97.
114. Chang H, Li B, Zhang X, Meng X. NCK1-AS1 promotes NCK1 expression to facilitate tumorigenesis and chemo-resistance in ovarian cancer. Biochem Biophys Res Commun 2020;522:292-9.
115. Zhao Y, He J, Li Y, Lv S, Cui H. NUSAP1 potentiates chemoresistance in glioblastoma through its SAP domain to stabilize ATR. Signal Transduct Target Ther 2020;5:44.
116. Aoki S, Inoue K, Klein S, et al. Placental growth factor promotes tumor desmoplasia and treatment resistance in intrahepatic cholangiocarcinoma. Gut 2022;71:185-93.
117. Sheng X, Li Y, Li Y, et al. PLOD2 contributes to drug resistance in laryngeal cancer by promoting cancer stem cell-like characteristics. BMC Cancer 2019;19:840.

118. Zhou Q, Perakis SO, Ulz P, et al. Cell-free DNA analysis reveals POLR1D-mediated resistance to bevacizumab in colorectal cancer. Genome Med 2020;12:20.
119. Narita K, Matsuda Y, Seike M, Naito Z, Gemma A, Ishiwata T. Nestin regulates proliferation, migration, invasion and stemness of lung adenocarcinoma. Int J Oncol 2014;44:1118-30.
120. Fan SH, Wang YY, Lu J, et al. CERS2 suppresses tumor cell invasion and is associated with decreased V-ATPase and MMP-2/MMP-9 activities in breast cancer. J Cell Biochem 2015;116:502-13.
121. Lulic L, Jakovcevic A, Manojlovic L, Dediol E, Banks L, Tomaic V. Human DLG1 and SCRIB Are Distinctly Regulated Independently of HPV-16 during the Progression of Oropharyngeal Squamous Cell Carcinomas: A Preliminary Analysis. Cancers (Basel) 2021;13.
122. Bandaru S, Zhou AX, Rouhi P, et al. Targeting filamin B induces tumor growth and metastasis via enhanced activity of matrix metalloproteinase-9 and secretion of VEGF-A. Oncogenesis 2014;3:e119.
123. Tamura RE, de Vasconcellos JF, Sarkar D, Libermann TA, Fisher PB, Zerbini LF. GADD45 proteins: central players in tumorigenesis. Curr Mol Med 2012;12:634-51.
124. Liu L, Liu H, Zhou Y, et al. HLTF suppresses the migration and invasion of colorectal cancer cells via TGFbeta/SMAD signaling in vitro. Int J Oncol 2018;53:2780-8.
125. Lee M, Venkitaraman AR. A cancer-associated mutation inactivates a region of the high-mobility group protein HMG20b essential for cytokinesis. Cell Cycle 2014;13:2554-63.
126. Lee CC, Lin JC, Hwang WL, et al. Macrophage-secreted interleukin-35 regulates cancer cell plasticity to facilitate metastatic colonization. Nat Commun 2018;9:3763.
127. Airoldi I, Cocco C, Giuliani N, et al. Constitutive expression of IL-12R beta 2 on human multiple myeloma cells delineates a novel therapeutic target. Blood 2008;112:750-9.
128. Li K, Wu JL, Qin B, et al. ILF3 is a substrate of SPOP for regulating serine biosynthesis in colorectal cancer. Cell Res 2020;30:163-78.
129. Xu HF, Huang TJ, Yang Q, et al. Candidate tumor suppressor gene IRF6 is involved in human breast cancer pathogenesis via modulating PI3K-regulatory subunit PIK3R2 expression. Cancer Manag Res 2019;11:5557-72.
130. Li D, Cheng P, Wang J, et al. IRF6 Is Directly Regulated by ZEB1 and ELF3, and Predicts a Favorable Prognosis in Gastric Cancer. Front Oncol 2019;9:220.
131. Wang Y, Wang G, Tan X, et al. MT1G serves as a tumor suppressor in hepatocellular carcinoma by interacting with p53. Oncogenesis 2019;8:67.
132. Liu Z, Ye Q, Wu L, et al. Metallothionein 1 family profiling identifies MT1X as a tumor suppressor involved in the progression and metastastatic capacity of hepatocellular carcinoma. Mol Carcinog 2018;57:1435-44.
133. Sharma A, Mendonca J, Ying J, et al. The prostate metastasis suppressor gene NDRG1 differentially regulates cell motility and invasion. Mol Oncol 2017;11:655-69.
134. Ye J, Liang R, Bai T, et al. RBM38 plays a tumor-suppressor role via stabilizing the p53-mdm2 loop function in hepatocellular carcinoma. J Exp Clin Cancer Res 2018;37:212.
135. Jiaze Y, Sinan H, Minjie Y, et al. Rcl1 suppresses tumor progression of hepatocellular carcinoma: a comprehensive analysis of bioinformatics and in vitro experiments. Cancer Cell Int 2022;22:114.
136. Yu S, Gautam N, Quan M, Gao Y. RSRC1 suppresses gastric cancer cell proliferation and migration by regulating PTEN expression. Mol Med Rep 2019;20:1747-53.
137. Jian H, Zhao Y, Liu B, Lu S. SEMA4B inhibits growth of non-small cell lung cancer in vitro and in vivo. Cell Signal 2015;27:1208-13.

138. Ding F, Gao F, Zhang S, Lv X, Chen Y, Liu Q. A review of the mechanism of DDIT4 serve as a mitochondrial related protein in tumor regulation. Sci Prog 2021;104:36850421997273.
139. Ho KH, Chen PH, Chou CM, et al. A Key Role of DNA Damage-Inducible Transcript 4 (DDIT4) Connects Autophagy and GLUT3-Mediated Stemness To Desensitize Temozolomide Efficacy in Glioblastomas. Neurotherapeutics 2020;17:1212-27.
140. Hao Y, Li G. Role of EFNA1 in tumorigenesis and prospects for cancer therapy. Biomed Pharmacother 2020;130:110567.
141. Cadranel J, Liu SV, Duruisseaux M, et al. Therapeutic Potential of Afatinib in NRG1 Fusion-Driven Solid Tumors: A Case Series. Oncologist 2021;26:7-16.
142. Durinikova E, Kozovska Z, Poturnajova M, et al. ALDH1A3 upregulation and spontaneous metastasis formation is associated with acquired chemoresistance in colorectal cancer cells. BMC Cancer 2018;18:848.
143. Gelardi ELM, Colombo G, Picarazzi F, et al. A Selective Competitive Inhibitor of Aldehyde Dehydrogenase 1A3 Hinders Cancer Cell Growth, Invasiveness and Stemness In Vitro. Cancers (Basel) 2021;13.
144. Hubert P, Roncarati P, Demoulin S, et al. Extracellular HMGB1 blockade inhibits tumor growth through profoundly remodeling immune microenvironment and enhances checkpoint inhibitor-based immunotherapy. J Immunother Cancer 2021;9.
145. Liu B, Huang G, Zhu H, et al. Analysis of gene coexpression network reveals prognostic significance of CNFN in patients with head and neck cancer. Oncol Rep 2019;41:2168-80.
146. Zhang X, Feng H, Li Z, et al. Application of weighted gene co-expression network analysis to identify key modules and hub genes in oral squamous cell carcinoma tumorigenesis. Onco Targets Ther 2018;11:6001-21.
147. Bard JE, Nowak NJ, Buck MJ, Sinha S. Multimodal Dimension Reduction and Subtype Classification of Head and Neck Squamous Cell Tumors. Front Oncol 2022;12:892207.
148. Schmidt M, Edlund K, Hengstler JG, et al. Prognostic Impact of Immunoglobulin Kappa C (IGKC) in Early Breast Cancer. Cancers (Basel) 2021;13.
149. Onieva JL, Xiao Q, Berciano-Guerrero MA, et al. High IGKC-Expressing Intratumoral Plasma Cells Predict Response to Immune Checkpoint Blockade. Int J Mol Sci 2022;23.
150. Liu K, Hoover AR, Krawic JR, et al. Antigen presentation and interferon signatures in B cells driven by localized ablative cancer immunotherapy correlate with extended survival. Theranostics 2022;12:639-56.
151. Slizhikova D, Zinovyeva M, Kuzmin D, et al. Decreased expression of the human immunoglobulin J-chain gene in squamous cell cancer and adenocarcinoma of the lungs. Molecular Biology 2007;41:594-600.
152. Isaeva OI, Sharonov GV, Serebrovskaya EO, et al. Intratumoral immunoglobulin isotypes predict survival in lung adenocarcinoma subtypes. J Immunother Cancer 2019;7:279.
153. Huang Z, Fan G, Wang D. Downregulation of calbindin 1, a calcium-binding protein, reduces the proliferation of osteosarcoma cells. Oncol Lett 2017;13:3727-33.
154. Chiang KC, Yeh TS, Wu RC, et al. Lipocalin 2 (LCN2) is a promising target for cholangiocarcinoma treatment and bile LCN2 level is a potential cholangiocarcinoma diagnostic marker. Sci Rep 2016;6:36138.
155. Sun Y, Jiang F, Pan Y, et al. XBP1 promotes tumor invasion and is associated with poor prognosis in oral squamous cell carcinoma. Oncol Rep 2018;40:988-98.
156. Liu J, Shen JX, Wu HT, et al. Collagen 1A1 (COL1A1) promotes metastasis of breast cancer and is a potential therapeutic target. Discov Med 2018;25:211-23.

157. Lin P, Tian P, Pang J, et al. Clinical significance of COL1A1 and COL1A2 expression levels in hypopharyngeal squamous cell carcinoma. Oncol Lett 2020;20:803-9.
158. Yuan L, Shu B, Chen L, et al. Overexpression of COL3A1 confers a poor prognosis in human bladder cancer identified by co-expression analysis. Oncotarget 2017;8:70508-20.
159. Kaur R, Chauhan A, Bhat SA, Chatterjee D, Ghoshal S, Pal A. Gene of the month: Cornulin. J Clin Pathol 2022;75:289-91.
160. Lee KM, Nam K, Oh S, et al. ECM1 regulates tumor metastasis and CSC-like property through stabilization of beta-catenin. Oncogene 2015;34:6055-65.
161. Wang Z, Zhou Q, Li A, Huang W, Cai Z, Chen W. Extracellular matrix protein 1 (ECM1) is associated with carcinogenesis potential of human bladder cancer. Onco Targets Ther 2019;12:1423-32.
162. Lin B, Zhang T, Ye X, Yang H. High expression of EMP1 predicts a poor prognosis and correlates with immune infiltrates in bladder urothelial carcinoma. Oncol Lett 2020;20:2840-54.
163. Johnson BD, Geldenhuys WJ, Hazlehurst LA. The Role of ERO1alpha in Modulating Cancer Progression and Immune Escape. J Cancer Immunol (Wilmington) 2020;2:103-15.
164. Zhang J, Pavlova NN, Thompson CB. Cancer cell metabolism: the essential role of the nonessential amino acid, glutamine. EMBO J 2017;36:1302-15.
165. Wang Z, Liu F, Fan N, et al. Targeting Glutaminolysis: New Perspectives to Understand Cancer Development and Novel Strategies for Potential Target Therapies. Front Oncol 2020;10:589508.
166. Filippou PS, Karagiannis GS, Musrap N, Diamandis EP. Kallikrein-related peptidases (KLKs) and the hallmarks of cancer. Critical reviews in clinical laboratory sciences 2016;53:277-91.
167. Zhou D, He Y, Li H, Huang W. KLK6 mediates stemness and metabolism of gastric carcinoma cells via the PI3K/AKT/mTOR signaling pathway. Oncol Lett 2021;22:824.
168. Filippou PS, Karagiannis GS, Musrap N, Diamandis EP. Kallikrein-related peptidases (KLKs) and the hallmarks of cancer. Crit Rev Clin Lab Sci 2016;53:277-91.
169. Yin L, Li Q, Mrdenovic S, et al. KRT13 promotes stemness and drives metastasis in breast cancer through a plakoglobin/c-Myc signaling pathway. Breast Cancer Res 2022;24:7.
170. Li Q, Yin L, Jones LW, et al. Keratin 13 expression reprograms bone and brain metastases of human prostate cancer cells. Oncotarget 2016;7:84645-57.
171. Li C, Liu X, Liu Y, et al. Keratin 80 promotes migration and invasion of colorectal carcinoma by interacting with PRKDC via activating the AKT pathway. Cell Death Dis 2018;9:1009.
172. Deng M, Xiong C, He ZK, et al. MCTS1 as a Novel Prognostic Biomarker and Its Correlation With Immune Infiltrates in Breast Cancer. Front Genet 2022;13:825901.
173. Reddy SP, Raslan WF, Gooneratne S, Kathuria S, Marks JE. Prognostic significance of keratinization in nasopharyngeal carcinoma. American journal of otolaryngology 1995;16:103-8.
174. Li A, Zhu X, Wang C, et al. Upregulation of NDRG1 predicts poor outcome and facilitates disease progression by influencing the EMT process in bladder cancer. Sci Rep 2019;9:5166.
175. Lim SC, Geleta B, Maleki S, Richardson DR, Kovacevic Z. The metastasis suppressor NDRG1 directly regulates androgen receptor signaling in prostate cancer. J Biol Chem 2021;297:101414.
176. Kataoka A, Yamada K, Hagiwara T, et al. Expression Status of Serine Protease 27: A Prognostic Marker for Esophageal Squamous Cell Carcinoma Treated with Preoperative Chemotherapy/Chemoradiotherapy. Ann Surg Oncol 2021;28:5373-81.

177. Xu X, Wei S, Chen Y, Yu D, Wang X, Dong X. Serum Small Proline-Rich Protein 2A (SPRR2A) Is a Noninvasive Biomarker in Gastric Cancer. Dis Markers 2020;2020:8493796.
178. Pandey R, Zhou M, Chen Y, et al. Molecular Pathways Associated with Kallikrein 6 Overexpression in Colorectal Cancer. Genes (Basel) 2021;12.
179. Pandey R, Zhou M, Islam S, et al. Carcinoembryonic antigen cell adhesion molecule 6 (CEACAM6) in Pancreatic Ductal Adenocarcinoma (PDA): An integrative analysis of a novel therapeutic target. Sci Rep 2019;9:18347.
180. Yap LF, Lai SL, Patmanathan SN, et al. HOPX functions as a tumor suppressor in head and neck cancer. Sci Rep 2016;6:38758.
181. Ren X, Yang X, Cheng B, et al. HOPX hypermethylation promotes metastasis via activating SNAIL transcription in nasopharyngeal carcinoma. Nat Commun 2017;8:14053.
182. Geng Z, Li J, Li S, et al. MAL protein suppresses the metastasis and invasion of GC cells by interfering with the phosphorylation of STAT3. J Transl Med 2022;20:50.
183. Lyukmanova EN, Bychkov ML, Sharonov GV, et al. Human secreted proteins SLURP-1 and SLURP-2 control the growth of epithelial cancer cells via interactions with nicotinic acetylcholine receptors. Br J Pharmacol 2018;175:1973-86.
184. Throm VM, Mannle D, Giese T, et al. Endogenous CHRNA7-ligand SLURP1 as a potential tumor suppressor and anti-nicotinic factor in pancreatic cancer. Oncotarget 2018;9:11734-51.
185. Wang Q, Lv Q, Bian H, et al. A novel tumor suppressor SPINK5 targets Wnt/beta-catenin signaling pathway in esophageal cancer. Cancer Med 2019;8:2360-71.
186. Azouz NP, Ynga-Durand MA, Caldwell JM, et al. The antiprotease SPINK7 serves as an inhibitory checkpoint for esophageal epithelial inflammatory responses. Sci Transl Med 2018;10.
187. Wu X, Cao W, Wang X, et al. TGM3, a candidate tumor suppressor gene, contributes to human head and neck cancer. Mol Cancer 2013;12:151.
188. Feng Y, Ji D, Huang Y, et al. TGM3 functions as a tumor suppressor by repressing epithelialtomesenchymal transition and the PI3K/AKT signaling pathway in colorectal cancer. Oncol Rep 2020;43:864-76.
189. Peng XC, Zeng Z, Huang YN, Deng YC, Fu GH. Clinical significance of TM4SF1 as a tumor suppressor gene in gastric cancer. Cancer Med 2018;7:2592-600.
190. Tang Q, Chen J, Di Z, et al. TM4SF1 promotes EMT and cancer stemness via the Wnt/beta-catenin/SOX2 pathway in colorectal cancer. J Exp Clin Cancer Res 2020;39:232.
191. Pastorekova S, Gillies RJ. The role of carbonic anhydrase IX in cancer development: links to hypoxia, acidosis, and beyond. Cancer Metastasis Rev 2019;38:65-77.
192. Doi T, Yoh K, Shitara K, et al. First-in-human phase 1 study of novel dUTPase inhibitor TAS-114 in combination with S-1 in Japanese patients with advanced solid tumors. Invest New Drugs 2019;37:507-18.
193. Arteaga CL. The epidermal growth factor receptor: from mutant oncogene in nonhuman cancers to therapeutic target in human neoplasia. J Clin Oncol 2001;19:32s-40s.
194. Ha H, Debnath B, Neamati N. Role of the CXCL8-CXCR1/2 Axis in Cancer and Inflammatory Diseases. Theranostics 2017;7:1543-88.
195. Guo P, Moses-Gardner A, Huang J, Smith ER, Moses MA. ITGA2 as a potential nanotherapeutic target for glioblastoma. Sci Rep 2019;9:6195.
196. Zheng M, Wu C, Yang K, et al. Novel selective hexokinase 2 inhibitor Benitrobenrazide blocks cancer cells growth by targeting glycolysis. Pharmacol Res 2021;164:105367.
197. Tripathi A, Shrinet K, Kumar A. HMGB1 protein as a novel target for cancer. Toxicol Rep 2019;6:253-61.

198. Cherradi S, Ayrolles-Torro A, Vezzo-Vie N, et al. Antibody targeting of claudin-1 as a potential colorectal cancer therapy. J Exp Clin Cancer Res 2017;36:89.
199. Ferrara N, Adamis AP. Ten years of anti-vascular endothelial growth factor therapy. Nat Rev Drug Discov 2016;15:385-403.
200. Mondal S, Roy D, Sarkar Bhattacharya S, et al. Therapeutic targeting of PFKFB3 with a novel glycolytic inhibitor PFK158 promotes lipophagy and chemosensitivity in gynecologic cancers. Int J Cancer 2019;144:178-89.
201. Liu Y, Zhou Y, Zhang P, Li X, Duan C, Zhang C. CHIP-mediated CIB1 ubiquitination regulated epithelial-mesenchymal transition and tumor metastasis in lung adenocarcinoma. Cell Death Differ 2021;28:1026-40.
202. Haberman VA, Fleming SR, Leisner TM, et al. Discovery and Development of Cyclic Peptide Inhibitors of CIB1. ACS Med Chem Lett 2021;12:1832-9.
203. Zhu W, Gliddon BL, Jarman KE, et al. CIB1 contributes to oncogenic signalling by Ras via modulating the subcellular localisation of sphingosine kinase 1. Oncogene 2017;36:2619-27.
204. Song KH, Oh SJ, Kim S, et al. HSP90A inhibition promotes anti-tumor immunity by reversing multi-modal resistance and stem-like property of immune-refractory tumors. Nat Commun 2020;11:562.
