# Supplementary material for: Deep spatial-omics analysis of Head & Neck carcinomas provides alternative therapeutic targets and rationale for treatment failure
Source: NPJ Precis Oncol. 2023 Sep 13;7:89. doi: 10.1038/s41698-023-00444-2 (PMC10499928; doi:10.1038/s41698-023-00444-2)
Supplement: Supplementary file 2 — Reporting Summary [file 41698_2023_444_MOESM2_ESM.pdf]

nature portfolio

Corresponding author(s): Jazmina L. Gonzalez Cruz; Quan Nguyen

Last updated by author(s): 2023_JUN_5

Reporting Summary

Nature Portfolio wishes to improve the reproducibility of the work that we publish. This form provides structure for consistency and transparency in reporting. For further information on Nature Portfolio policies, see our Editorial Policies and the Editorial Policy Checklist.

Statistics

For all statistical analyses, confirm that the following items are present in the figure legend, table legend, main text, or Methods section.

n/a	Confirmed	
☒	☐	The exact sample size (*n*) for each experimental group/condition, given as a discrete number and unit of measurement
☒	☐	A statement on whether measurements were taken from distinct samples or whether the same sample was measured repeatedly
☒	☐	The statistical test(s) used AND whether they are one- or two-sided<br>*Only common tests should be described solely by name; describe more complex techniques in the Methods section.*
☒	☐	A description of all covariates tested
☒	☐	A description of any assumptions or corrections, such as tests of normality and adjustment for multiple comparisons
☒	☐	A full description of the statistical parameters including central tendency (e.g. means) or other basic estimates (e.g. regression coefficient) AND variation (e.g. standard deviation) or associated estimates of uncertainty (e.g. confidence intervals)
☒	☐	For null hypothesis testing, the test statistic (e.g. *F*, *t*, *r*) with confidence intervals, effect sizes, degrees of freedom and *P* value noted<br>*Give P values as exact values whenever suitable.*
☒	☐	For Bayesian analysis, information on the choice of priors and Markov chain Monte Carlo settings
☒	☐	For hierarchical and complex designs, identification of the appropriate level for tests and full reporting of outcomes
☒	☐	Estimates of effect sizes (e.g. Cohen's *d*, Pearson's *r*), indicating how they were calculated

Our web collection on statistics for biologists contains articles on many of the points above.

Software and code

Policy information about availability of computer code

Data collection	Custom codes have been uploaded to: https://github.com/BiomedicalMachineLearning/HeadAndNeck_DeepCaseStudy.
Data analysis	Custom codes have been uploaded to: https://github.com/BiomedicalMachineLearning/HeadAndNeck_DeepCaseStudy.

For manuscripts utilizing custom algorithms or software that are central to the research but not yet described in published literature, software must be made available to editors and reviewers. We strongly encourage code deposition in a community repository (e.g. GitHub). See the Nature Portfolio guidelines for submitting code & software for further information.

Data

Policy information about availability of data

All manuscripts must include a data availability statement. This statement should provide the following information, where applicable:

- Accession codes, unique identifiers, or web links for publicly available datasets
- A description of any restrictions on data availability
- For clinical datasets or third party data, please ensure that the statement adheres to our policy

This study's raw sequencing data (fastq and BAM files) is now under controlled access via GEO (https://www.ncbi.nlm.nih.gov/geo/). PhenoCycler images and processed Visium gene expression matrices are publicly available and can be freely downloaded from UQ e-space, at https://doi.org/10.48610/698bb9e. All scripts used in this study are open-source and stored on our GitHub (https://github.com/BiomedicalMachineLearning/HeadAndNeck_DeepCaseStudy).

Research involving human participants, their data, or biological material

Policy information about studies with human participants or human data. See also policy information about sex, gender (identity/presentation), and sexual orientation and race, ethnicity and racism.

Reporting on sex and gender	Only samples from two male individuals are included in our work.
Reporting on race, ethnicity, or other socially relevant groupings	Our work does not need the inclusion of race, ethnicity, or any other social grouping.
Population characteristics	Two individuals were included in our study with the following characteristics:<br>Case presentation (extended)<br>A 60-year-old male ex-smoker with a 10 pack-year history presented with de novo oligometastatic disease, having been diagnosed with a p16 positive SCC primary tumor of the right tonsil and biopsy-proven left upper lobe lung metastasis (Figure 1A-C). He received concurrent chemoradiotherapy comprising weekly cisplatin and 70Gy in 35 fractions of radiation targeting the primary tumor and bilateral level II and III neck nodes (Figure 1A). Additionally, he received stereotactic radiation to the left lung nodule of 50Gy in 5 fractions, completed in June 2019.<br>The patient underwent disease re-assessment three months later, with PET/CT demonstrating multiple new bilateral pulmonary metastases and no locoregional disease (Figure 1B-C). He commenced treatment of 480mg nivolumab every 4 weeks and a CT scan after three cycles revealed resolution of two nodules and a significant reduction in a third (Figure 1B-C, yellow line), but minor growth of a left lower lobe nodule (Figure 1B-C, pink line). This single nodule was then treated with stereotactic radiation of 48Gy in four fractions and nivolumab was continued. Regular imaging confirmed stable intrathoracic disease for a further 13 months but a PET/CT scan in February 2021 demonstrated local recurrence of disease with a 22mm lesion of the left soft palate (biopsy MAR21) and progression of metastatic lesions in the lungs bilaterally.<br>The patient was then enrolled in a clinical trial (LEAP-009) and randomized to the treatment of pembrolizumab 200mg every 3 weeks and 20mg Lenvatinib orally daily. After demonstrating an early partial response radiologically, including autoamputation of the oropharyngeal recurrence with absence of measurable disease and a reduction in lung metastases, a treatment delay was required due to oropharyngeal pain and severe non-healing ulceration of the oropharynx. This subsequently settled and the patient recommenced pembrolizumab and dose-reduced Lenvatinib (14mg orally daily) in July 2021. The measurable disease remained stable radiologically until January 2022, although mucosal changes over the tonsillar fossa and soft palate were suspicious clinically from September (biopsy SEP21). Pembrolizumab and Lenvatinib were ceased, and the patient came off trial in early February 2022 when MRI scan confirmed definite disease progression involving the oropharynx.<br>Case presentation of additional patient<br>A 63-year-old male non-smoker presented with de novo keratinizing p16 positive SCC primary tumor of the left tonsil invading into the skeletal muscle with no nodal disease. He underwent transoral robotic-assisted resection (TORS) of tonsil and selective neck dissection of cervical lymph nodes in November 2020. No adjuvant radiotherapy was given. A two-year MRI follow-up indicates no tumor masses or recurrent disease.
Recruitment	Cancer patients<br><br>OPSCC cancer patients will be selected from the Head & Neck databases from Princess Alexandra Hospital (PAH. Brisbane, Australia), and will be asked to participate if inclusion and exclusion criteria are satisfied. Patients will be consented by a member of the study team with no pre-existing relationship with the patient, such as independent nurse or a senior laboratory research assistant, and who has been trained in the consent process and who has been delegated to obtain informed consent. The voluntary nature of participation will be made clear, and necessary documentation supplied by trained study team members holding a valid Good Clinical Practice certificate (GCP). Consent will be required prior to participation in the study. Volunteer participants will have the patient's information and consent process explained initially by trained members of the study team and allowed reasonable time (minimum 30 minutes) to consider the consent to tumour bio-specimens. Prior the day of collection at PAH the volunteer will have the form discussed and will sign consent if happy to proceed. The volunteer will sign in the presence of the nurse as well as the Project Investigator. Consent forms will be stored in a locked cabinet in a locked office within the Translations Research Institute and according to The University of Queensland data storage specifications as detailed in ethics application.
Ethics oversight	This study was approved by the Metro South Human Research Ethics Committee (approval #HREC/2019/QMS/49990) and The University of Queensland (approval #2019001021) and conducted in accordance with the Declaration of Helsinki. Participants provided written consent after receiving a Participant Information and Consent Form. Written informed consent was obtained from the individuals for the publication of any potentially identifiable images or data included in this article.

Note that full information on the approval of the study protocol must also be provided in the manuscript.

Field-specific reporting

Please select the one below that is the best fit for your research. If you are not sure, read the appropriate sections before making your selection.

☒ Life sciences ☐ Behavioural & social sciences ☐ Ecological, evolutionary & environmental sciences

For a reference copy of the document with all sections, see nature.com/documents/nr-reporting-summary-flat.pdf

Life sciences study design

All studies must disclose on these points even when the disclosure is negative.

Sample size	2
Data exclusions	None.
Replication	None.
Randomization	None.
Blinding	None.

Reporting for specific materials, systems and methods

We require information from authors about some types of materials, experimental systems and methods used in many studies. Here, indicate whether each material, system or method listed is relevant to your study. If you are not sure if a list item applies to your research, read the appropriate section before selecting a response.

Materials & experimental systems

n/a	Involved in the study	
☐	☒	Antibodies
☒	☐	Eukaryotic cell lines
☒	☐	Palaeontology and archaeology
☒	☐	Animals and other organisms
☒	☐	Clinical data
☒	☐	Dual use research of concern
☒	☐	Plants

Methods

n/a	Involved in the study	
☒	☐	ChIP-seq
☒	☐	Flow cytometry
☒	☐	MRI-based neuroimaging

Antibodies

Antibodies used

AKOYA Antibody, AKOYA Tag number, AKOYA PIN, Target cell

CD20, BX007, 4450018, B and T cell subsets
PanCK, BX019, 4450020, Epithelial cells
CD8a, BX026, 4250012, T cell subsets, NK subset
Ki67, BX047, 4250019, Proliferating cells
CD45Ro, BX017, 4250023, Activated and memory T cells, B cell subsets
CD3e, BX045, 4450030 ,T cells, NK-T cells, thymocytes
CD107a, BX006, 4350001, Degranulation marker
HLA-DR, BX033, 4450029, Activated B cells, antigen presenting cells
CD4, BX003, 4350018, T cell subsets, monocytes, macrophages
CD68, BX015, 4350019, Monocytes, macrophages
CD45, CX021, 4450042, Hematopoietic cells
PD-1, RX046, 4550038, Activation/exhaustion
PD-L1, BX043, 4550072, Inhibitory signal

Validation

Antibody specificity has been tested by the manufacturer AKOYA, based on one or more of the following processes:

Binary Strategy. To test an antibody in biologically relevant positive and negative expression systems. Binary models may include endogenous cells or tissues in which expression of the target protein is known or predicted to be positive or negative, as well as genetic knockouts, or use of treatments to induce or inhibit expression.

Ranged Strategy. By utilizing both endogenous and heterologous models that express high, moderate, and low levels of the target of interest, a ranged approach helps reveal optimal working conditions for a given antibody. Ranged models rely on differences in target expression or modification that are not black and white –and are typically more reflective of real biology. Typical methods include siRNA and heterozygous knockout assays.

Orthogonal Strategy. Cross-referencing antibody results using data from non-antibody-based methods and resources. Orthogonal approaches include evaluating previously published results, studying expression analysis using various 'omics techniques (e.g., genomics, transcriptomics, or proteomics), performing in situ hybridization, RNA sequencing, and mass spectrometry.

Multiple Antibody Strategy. This approach compares the signal of the antibody of interest to that of antibodies targeting non-overlapping epitopes of the desired target. A common method is to first immunoprecipitate (IP) the target with one antibody, then

subsequently detect it by western blot, using another antibody against the same target. This helps verify that both antibodies bind to the correct molecule. In addition to IP, ChIP and ChIP-seq are commonly used methods for the multiple antibody strategy.

Heterologous Strategy. This approach involves evaluating the antibody signal in cell lines following heterologous expression of a native or mutated target protein. Useful for verifying cross-reactivity of an antibody with protein isoforms or conserved family members, it can also reveal potential for off-target binding due to antigen homology. Recombinant proteins may be used to identify specificity of an antibody for one or more members of a protein family. Alternatively, DNA plasmids designed for exogenous expression of antigenic targets of interest can be analyzed by western blot or immunocytochemical staining techniques.

Complementary Strategies. To use complementary assays, such as competitive ELISA, peptide dot blots, peptide blocking, or protein arrays. These techniques can provide important information about antibody specificity and functionality.
